# Clinical and Economic Burden of Diseases with Accelerated Approval Therapies: Evidence of Unmet Patient Need

**DOI:** 10.1007/s43441-026-01030-x

**Published:** 2026-08-08

**Authors:** Hanke Zheng, Shanshan Wang, Jason Shafrin, Shravani Bheema, Kathryn Spurrier, Jonathan D. Campbell

**Affiliations:** 1 National Pharmaceutical Council, 1717 Pennsylvania Avenue, NW, Suite 800, Washington, D.C., 20006 USA; 2 FTI Consulting, Washington, D.C., USA; 3 FTI Consulting, Los Angeles, USA; 4 University of Southern California, Los Angeles, USA

**Keywords:** Accelerated approval, Economic evaluation, Productivity loss, Economic burden, Rare disease

## Abstract

**Background:**

Some US payers delay coverage for non-oncology treatments approved via the Food and Drug Administration (FDA)’s accelerated approval (AA) pathway.

**Objectives:**

To estimate the economic burden and characteristics of diseases with therapies approved via the AA pathway compared to diseases with no AA therapies.

**Methods:**

The study identified the initial FDA-approved AA indications for all non-oncology therapies from January 2015 to February 2025. For comparison, 100 non-oncology diseases with the most recent FDA-approved non-AA therapies were identified. Annual economic burden and disease characteristics were extracted from peer-reviewed literature, with costs inflated to 2025 USD. Economic burden studies that used a societal perspective were prioritized. Comparative analyses were conducted using the Mann–Whitney U Test.

**Results:**

Thirty-two non-oncology AA treatments were identified representing 16 unique diseases. Average annual incremental societal costs for AA diseases were 1.4 times higher than for non-AA diseases ($91,857 vs. $65,468, *p* = 0.012). Additionally, the annual average productivity loss and caregiver burden were 1.8 times ($17,635 vs. $9,620, *p* = 0.092) and 2.4 times ($20,268 vs. $8,294, *p* = 0.055) greater in the AA diseases, respectively. Diseases with newly-approved AA treatments were characterized by shorter life expectancy (59.3 vs 65.1 years), lower average quality of life (0.64 vs 0.69), and more likely to be rare diseases (68.8% vs 53.1%) compared to non-AA diseases.

**Conclusion:**

Non-oncology diseases with treatments approved via the AA pathway imposed a higher economic burden and had more severe disease characteristics compared to non-AA diseases. These findings should be considered alongside AA coverage policy decision-making.

**Supplementary Information:**

The online version contains supplementary material available at https://doi.org/10.1007/s43441-026-01030-x.

## Introduction

The Accelerated Approval (AA) Program was established by the US Food and Drug Administration (FDA) in 1992 to expedite the availability of treatments for serious or life-threatening diseases. The program allows therapies to be approved based on surrogate endpoints, which are markers or laboratory measurements that are reasonably likely to predict clinical benefit, rather than direct evidence of patient improvement [1]. This pathway is most often used for rare diseases, oncology, and genetic diseases, where traditional trials may be too long or difficult [2]. While the AA program allows earlier patient access, manufacturers are required to conduct post-marketing confirmatory trials to verify the clinical benefit [3]. If these trials fail to verify clinical benefit, the FDA can withdraw the therapy. The program aims to balance patient unmet needs and regulatory oversight, especially where there are no existing effective therapies [2].

Coverage of AA-approved therapies by US payers, however, is not guaranteed. The Centers for Medicaid and Medicare (CMS), for example, may delay coverage decisions or impose restrictions, leading to gaps between approval and patient access [4]. In February 2023, CMS, through the Accelerating Clinical Evidence Model, announced that it would pay less for therapies approved via FDA’s AA pathway until a clinical benefit has been confirmed by the required confirmatory studies, however this program was ultimately never implemented. Similarly, commercial insurers, particularly those in Medicare Advantage and employer-sponsored plans, have increasingly begun to deny or delay reimbursement for therapies approved via the AA pathway until sufficient evidence emerges to merit full FDA approval [5]. For example, in 2025, Independence Blue Cross in Pennsylvania excluded non-oncology therapies from reimbursement for 18 months after AA [6].

As payers consider whether or not to delay coverage for treatments approved under the AA pathway, populations with high unmet medical needs and economic burden may be disproportionately affected. While evaluating the impact of delayed coverage for accelerated approval therapies on patient outcomes would be highly informative, such analyses are currently limited by the recent implementation of coverage restrictions and the multifactorial nature of reimbursement decisions (e.g., clinical uncertainty, pricing, and payer policies). In the absence of such direct evidence, characterizing the economic burden and disease profile of conditions with AA therapies serves as a meaningful proxy for informing AA coverage decisions. This study aims to characterize the economic burden and disease profile of non-oncology conditions with therapies approved via the AA pathway, and compared to diseases without an AA-approved therapy approved over the same time period.

## Materials and Methods

This study employed a cross-sectional, disease-level comparative design to estimate and compare unmet need between diseases with AA therapies and those treated by non-AA therapies in the non-oncology setting from a U.S. societal perspective. Unmet need is characterized based on the economic burden and disease characteristics. The economic burden was assessed prior to availability of AA therapies, capturing the baseline burden they were intended to address and informing evaluation of coverage delays on patient access.

To implement this estimation in practice, a four step approach was used. First, FDA-approved AA therapies and non-AA therapies in non-oncology space were identified. Second, data were then collected and analyzed on the economic burden and disease characteristics associated with the indications for which these therapies were approved. Third, these data were then compared between the AA and non-AA groups. Finally, the caregiving and productivity losses of AA-treated and non-AA-treated diseases were estimated based on both the literature and the Jiao–Basu approach as a secondary analysis, and sensitivity analyses were conducted [7].

### Identification of Therapies and Diseases

Using the FDA’s Center for Drug Evaluation and Research (CDER) novel drug approval website, all FDA-approved non-oncology AA therapies from January 2015 to February 2025 and their initial indications were identified. For comparison, this study identified 100 unique non-oncology diseases treated by recent therapies approved through the traditional FDA pathway. Eligible non-AA approvals were reviewed in reverse chronological order and mapped to their corresponding diseases based on the drug’s first FDA-approved indication. Because multiple therapies may be approved for the same disease, more than 100 approvals were reviewed to identify 100 unique non-AA diseases. Diseases were classified hierarchically and mutually exclusively based on whether they had any AA-approved therapy during the study period (January 2015 to February 2025). Diseases with at least one AA-approved therapy were classified as AA-treated diseases, including diseases that also had therapies approved through the traditional pathway. Diseases without any AA-approved therapy during the study period were classified as non-AA diseases. Using this approach, the 100 non-AA diseases were identified from approvals between June 21, 2018 and February 2025 (See S14 from Supplemental Appendix). Comparison was made to the most recent approvals to ensure temporal comparability with AA approvals and also to reflect the latest therapeutic landscape [8]. The “AA date” was defined as the date on which the therapy received its first indicated approval (i.e., New Drug Application (NDA) for small molecules or Biologics License Application (BLA) for biologics) and entered the market through the AA pathway. Based on the indications specified in FDA labels, a high-level disease and therapeutic area were assigned to each therapy according to FDA classifications. Excluded therapies were those indicated for (1) non-specific diseases (e.g., pain or symptom management or imaging/diagnostic tools), (2) cosmetic or nonmedical diseases (e.g., birth control, acne treatment, etc.), (3) diseases without recent cases in the United States (e.g., SmallPox/MPox) and (4) oncology indications. Oncology indications were excluded as many payer policies prohibiting coverage of AA therapies make an exception for oncology products [6].

### Identification of Economic Burden and Disease Characteristics

Data on economic burden and key characteristics of each identified disease were extracted through a targeted literature search primarily using PubMed and Google Scholar. Peer-reviewed, US-based, and more recent studies were prioritized when multiple burden studies were available (S15 from Supplemental Appendix). When peer-reviewed publications were not available, news articles, white papers, and patient advocacy websites were used. Following the approach used in a previous study by Shafrin et al. [9], the annual economic burden was estimated from the US societal perspective, consisting of the following components: (1) direct medical costs (disaggregated as inpatient, outpatient, pharmacy, emergency, and other costs, where available), (2) direct nonmedical costs, (3) indirect costs from patient productivity losses, and (4) indirect costs from informal caregiving specific to the disease. Direct medical costs excluding prescription medication were also reported. In the case that studies did not directly report productivity losses, annual hours lost were multiplied with the median US hourly wage, sourced from the Bureau of Labor Statistics [10], to estimate productivity loss. All costs were inflated to 2025 USD using the Medical Care Component of the Consumer Price Index (CPI) [11]. When specific cost components were not reported, values were treated as missing and handled according to the aggregation approaches described in “Data analysis” section. Next, eight characteristics were collected for each disease, including average age of onset, percentage of patients aged < 18 years, percentage of patients aged ≥ 65 years, life expectancy (LE), life years (LY) after diagnosis, disease duration, average patient quality of life (QoL), and disease prevalence per 100,000 [9]. Life-year loss for patients was calculated as the difference between the LY after diagnosis for the patient population and the average remaining life expectancy for the general population of the same age (based on the average age at diagnosis). General population life expectancy was sourced from US Life Tables [12]. The LY after diagnosis, when not available from the literature, was calculated by subtracting the age of onset of the disease from LE. Conversely, LE, when not available from the literature, was calculated using life-years after diagnosis and age of onset. QoL values ranged from 0 to 1, where 0 means death and 1 means perfect health [13]. If proportion of a disease population aged < 18 and/or ≥ 65 was not available and the disease does not disproportionally affect a specific age group, the U.S. Census 2020 proportions for population < 18 or ≥ 65 were assumed [14].

### Data Analysis

To compare the economic burden between AA and non-AA therapies, costs were aggregated costs across categories using a simple average, dividing the sum of costs for all diseases in the AA (or non-AA) cost categories by the number of AA (or non-AA) diseases in the study. To compare disease characteristics, the average value of each of the 8 identified disease characteristics were compared between the AA and non-AA groups. Additionally, threshold-based descriptive summaries were used to characterize the proportion of diseases meeting markers of disease severity. These thresholds were selected a priori to identify diseases with limited expected survival after diagnosis, moderate-to-severe impairment in health-related quality of life, and rare disease status which was defined using the standard FDA-based US threshold of fewer than 200,000 affected individuals [15]. Specifically, the proportion of AA and non-AA diseases with LY after diagnosis less than or equal to 10 years, and QoL scores below 0.5 and 0.6, and the proportion with rare disease indications were also calculated. Statistical significance of group differences in both economic burden and disease characteristics between AA and non-AA groups was tested using the Mann–Whitney U test, selected due to the non-normal distribution of burden data and the relatively small sample size of AA-related diseases.

### Scenario Analyses for Economic Burden

Three key issues when estimating the economic burden of diseases included the consideration of missing values, potential publication bias, and heterogeneity in economic burden across geographies. In this study, the baseline methodology using simple averages across cost categories had two key limitations: due to missing values, the number of diseases in each cost category varied, resulting in the cost categories not always adding up to the average societal costs. Additionally, the results may be biased if published studies disproportionately focus on diseases with higher economic burden, or heterogeneity in disease cost across countries impacts the burden estimates.

To verify the robustness of the results, five scenario analyses were conducted: (1) a “conservative” cost aggregation approach, (2) a “less conservative” cost aggregation approach, (3) an alternative methodology for calculating productivity and caregiving cost, (4) expanded analysis to include additional diseases that received AA approvals within the study period, not limiting to the first-approved indication, and (5) an analysis with the economic burden re-estimated using only U.S. studies. The first “conservative” approach assumed that all missing non-medical costs were equal to zero. While this approach ensured that the disaggregated cost items added up to the total average societal costs, it underestimated the non-medical costs as all missing data was set to zero. The second “less conservative” scenario assumed that missing non-medical costs were equal to the average percentage of indirect medical costs as a share of total societal costs for available diseases. Although this approach ensured that the disaggregated cost items added up to the total average societal costs, it may overestimate non-medical costs if diseases without published non-medical costs tend to have lower non-medical costs than diseases that have published studies on these broader cost outcomes. To account for the absence of published literature and standardize the estimating approach across diseases, a third data-driven approach was used to extrapolate productivity and caregiving based on the Jiao-Basu algorithm and applied to 2021 Medical Expenditure Panel Survey data [7]. The Jiao-Basu algorithm estimates a disease’s impact on patients’ productivity and caregiving based on patient’s age, QoL, and disability days (S16 from Supplemental Appendix). Fourth, as the baseline analysis limited to AA diseases approved as the initial indication, the sample was expanded to include all AA indications approved during the study period, regardless of whether they represented the first indication. Finally, the average economic burden was re-estimated after excluding non-US studies.

## Results

Based on the selection criteria, the study identified 32 indications with AA-approved therapies, representing 141 indications with non-AA-approved therapies. The base case sample included 16 AA-treated diseases and 100 diseases treated by the most recently approved non-AA therapies. Examples of diseases in the AA samples include primary immunoglobulin A nephropathy (IgAN), light chain amyloidosis, sickle cell disease, and Duchenne muscular dystrophy (DMD).

### Economic Burden

**Fig. 1:**
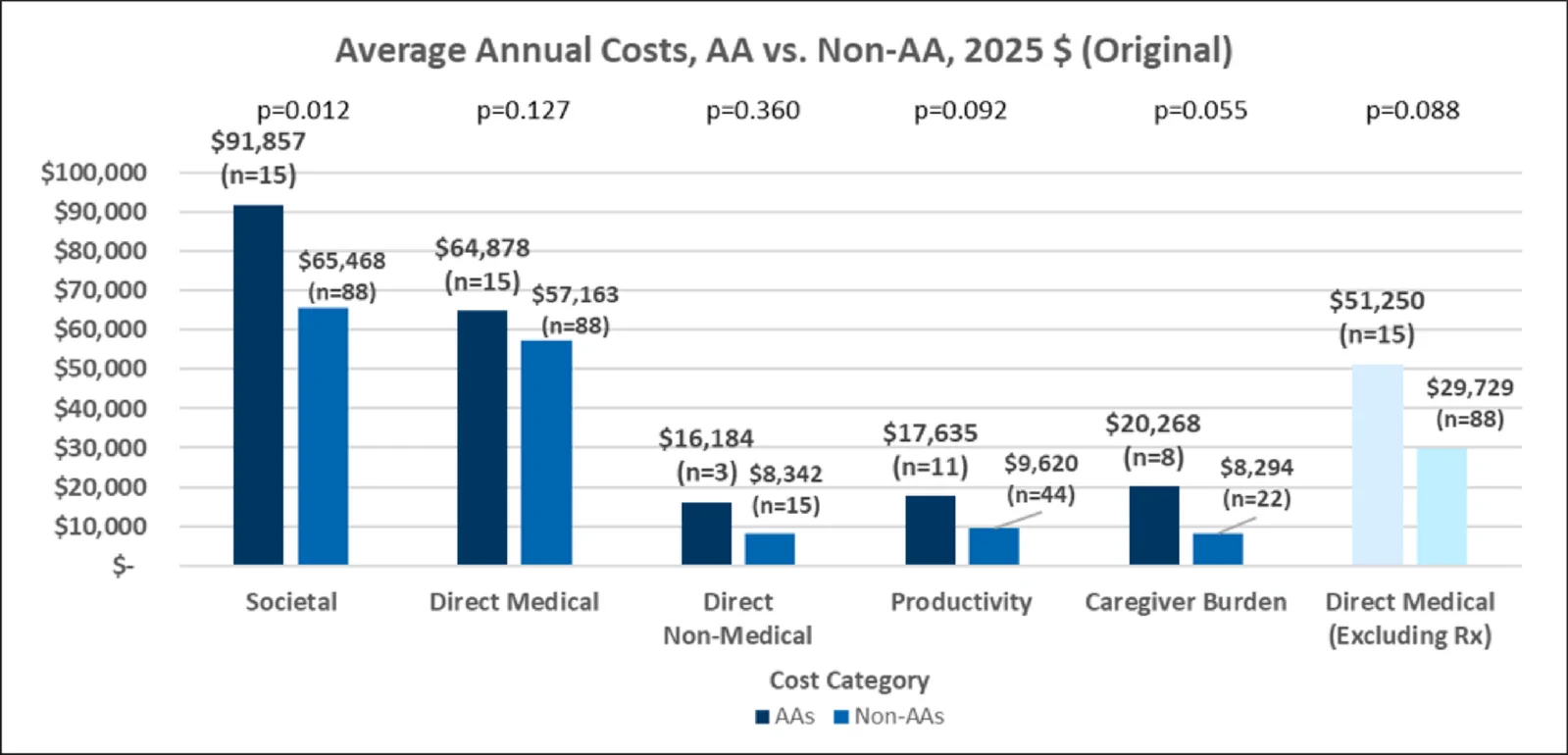
Annual US Societal Economic Burden in AA vs non-AA diseases. *Notes*: n denotes the number of conditions with available data included in each estimate. The last two bars (shown in a distinct color) represent direct medical costs excluding Rx costs and are a subset of total direct medical costs, not a separate cost category

AA-treated diseases were found to have higher annual societal costs on average, with $91,857 (SD: $81,327) compared to $65,468 (SD: $129,379) for non-AA diseases, representing a 40.3% (*p* = 0.012) greater societal costs. Specifically, AA-treated diseases incurred higher direct medical costs ($64,878 vs $57,163, Δ = 13.5%, *p* = 0.127), direct non-medical costs ($16,184 vs $8,342, Δ = 94.0%, *p* = 0.360), productivity costs ($17,635 vs $9,620, Δ = 83.3%, *p* = 0.092), caregiver burden ($20,268 vs $8,294, Δ = 144.4%, *p* = 0.055), and direct medical costs excluding prescription costs ($51,250 vs $29,729, Δ = 72.4%, *p* = 0.088) as illustrated in Fig. 1, however these sub-category estimates were not statistically significant. Additional details on these estimates are available in Supplementary Table S2.

### Disease Characteristics

**Fig. 2:**
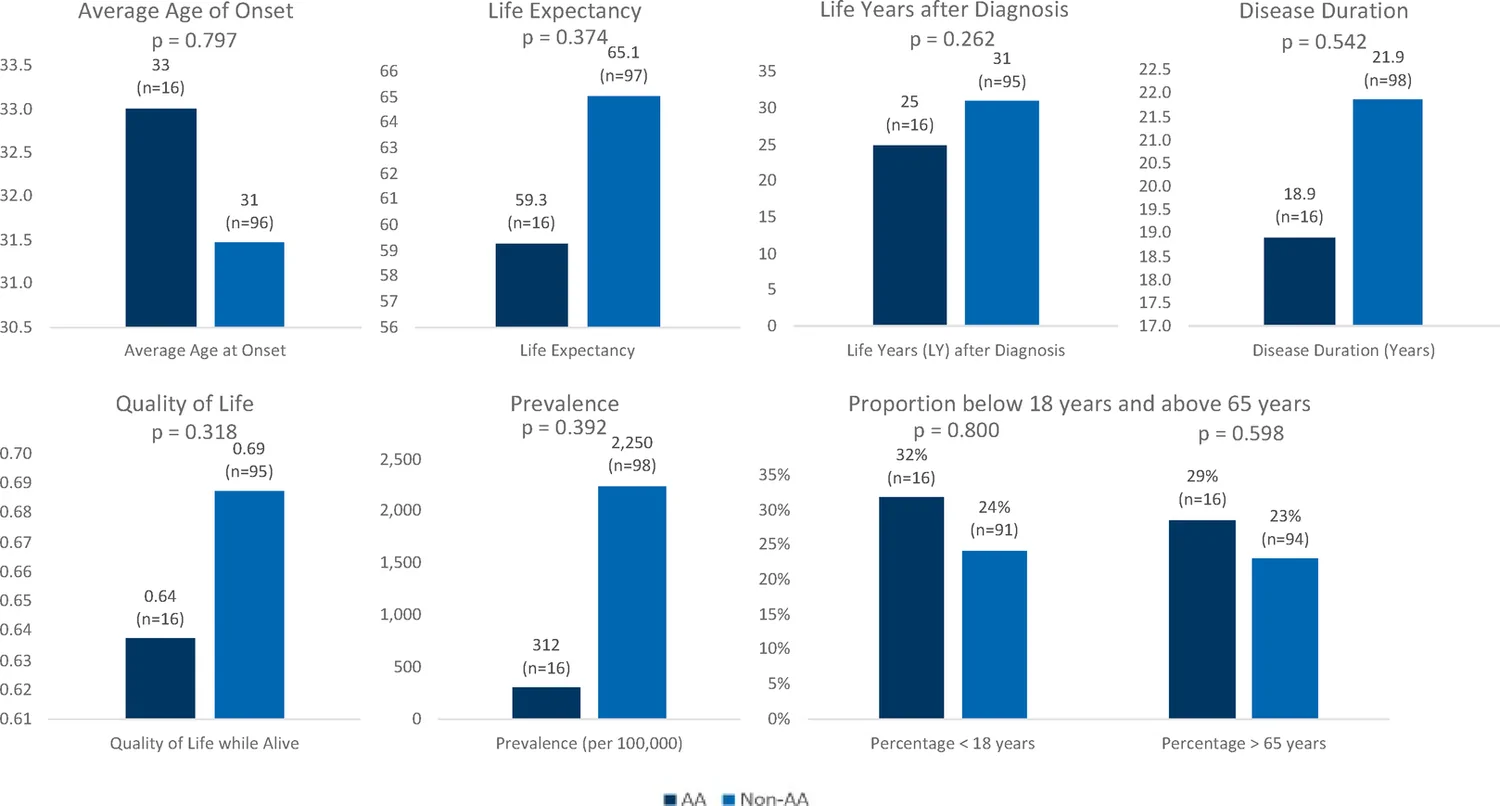
Disease characteristics of AA and non-AA diseases. *Notes*: n denotes the number of conditions with available data included in each estimate

Similar to the economic burden estimates, differences in disease characteristics were not statistically significant between AAs and non-AAs. Nonetheless, AA-treated diseases were estimated to affect a greater proportion of children and elderly than non-AA-treated diseases. Specifically, 31.9% of patients were under the age of 18 for AA-treated diseases, compared to 24.2% for non-AA-treated diseases. Similarly, 28.7% of patients with AA-treated diseases were above the age of 65, compared to 23.1% for non-AA-treated diseases. On average, the prevalence of AA-treated diseases was significantly lower, with 312 cases per 100,000, compared to 2,250 cases per 100,000 for non-AA-treated diseases, as illustrated in Fig. 2.

Additionally, AA-treated diseases were more likely to be rare diseases, have lower QoL, and have a shorter life expectancy on average. A greater proportion of AA-treated diseases had a rare disease designation compared with non-AA-treated diseases (68.8% vs 53.1%). Further, 37.5% of AA-treated diseases had an average QoL score ≤ 0.6, compared to 23.2% of non-AA-treated diseases. This difference was also large for QoL scores ≤ 0.5, with 18.8% of AA-treated diseases meeting this criterion compared to 9.5% of non-AA-treated diseases. Furthermore, patients with AA-treated diseases were more likely to have shorter LY after diagnosis, with 37.5% of AA-treated diseases having a LY of ≤ 10 years (mean: 25.0, SD: 19.6), compared to 21.1% of non-AA-treated diseases (mean: 31.0, SD: 20.6), as summarized in Table 1. These threshold-based comparisons were descriptive and were not statistically significant. Further details on these estimates can be found in Supplementary Tables and Figs. S3–S6.

**Table 1:** Proportion of diseases by disease characteristics threshold (AAs vs. Non-AAs)

Group	Measure	Life years after diagnosis < 10	QoL ≤ 0.5	QoL ≤ 0.6	Rare disease designation (< 200 k individuals in the US)
AA	Count	6	3	6	11
	Proportion	37.5%	18.8%	37.5%	68.8%
Non-AA	Count	20	9	22	52
	Proportion	21.1%	9.5%	23.2%	53.1%

*AA* Accelerated approval, *QoL* Quality of life. *Count* denotes the number of conditions with available data included in each estimate

### Scenario Analysis

Across all scenario analyses, diseases with AA-approved therapies had numerically higher societal burden compared to diseases without an AA-approved therapy. The finding held across both our “conservative” and “less conservative” scenarios. In the conservative scenario, societal costs were 40.3% higher for AA-treated diseases, driven by numerically greater non-medical (127.6%, *p* = 0.505), and statistically significant differences in productivity (168.9%, *p* = 0.023), and caregiver costs (421.3%, *p* = 0.012). The less conservative scenario analysis yielded smaller but still substantial differences (societal: 31.3%, *p* = 0.022; non-medical: 55.9%, *p* = 0.103; productivity: 54.4%, *p* = 0.103; caregiver burden: 129.0%, *p* = 0.004). Direct medical costs excluding prescription costs remained approximately 72% higher in both scenarios. Results are presented in S1 and S10.

**Fig. 3:**
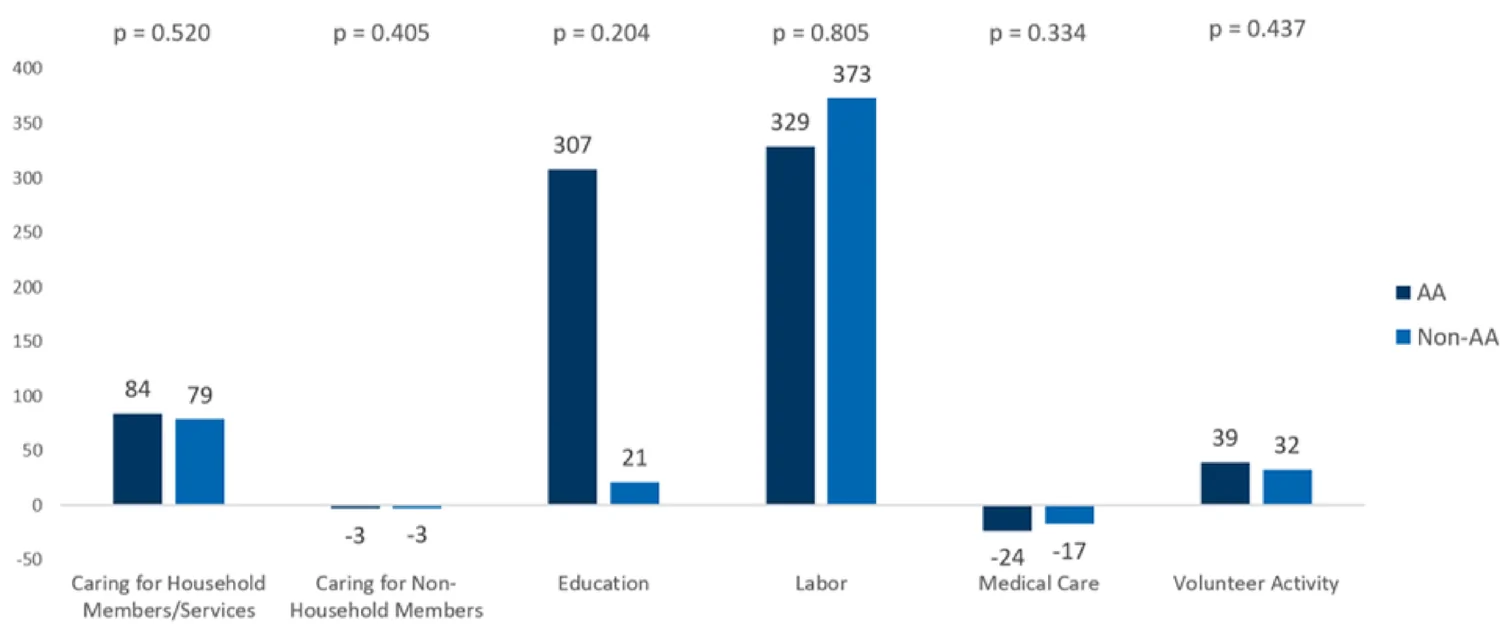
Annual hours of productivity lost among AA and non-AA diseases. *Notes*: AA estimates were based on n = 16 diseases, as each AA disease was matched to a proxy disease. Non-AA estimates were based on n = 38 diseases

In the data-driven approach to estimating productivity and caregiving costs analysis (i.e., based on Jiao-Basu), results confirmed that AA-treated diseases incurred greater annual caregiving and productivity losses ($17,845 vs. $11,814; Δ = $6,031), with particularly large differences in education- and care-related hours, although the differences were not statistically significant (Fig. 3 and Supplementary Tables S7–S9). Alternative scenarios utilizing different proxies for AA-treated diseases suggested disparities could reach $12,426.

Expanding the AA-definition to include all AA-indications increased the AA sample size from n = 16 to n = 18 diseases. This produced results that were similar to the base case (Supplementary Table S11), with societal costs $30,216 ($95,684 vs. $65,468, *p* = 0.003) higher for AA-treated versus non-AA-treated diseases (Supplementary Table S12–S13, S17 from Supplemental Appendix).

Finally, after removing diseases which only had evidence from non-US economic burden studies (4% of diseases), the results were also robust to the baseline findings (S18 from Supplemental Appendix).

## Discussion

The economic burden prior to AA therapy approval within non-oncology diseases was numerically higher than that of non-AA-treated diseases, with a large amount of uncertainty due to the small sample size of diseases with approved AA therapies. For policy relevance and interpretation clarity, the study focused on the unmet needs associated with those diseases that existed before the availability of the AA or non-AA treatment of interest. On average, the societal costs associated with AA-treated diseases were 40.3% greater than those of non-AA diseases. This burden includes not only higher medical costs but also higher caregiver burden and higher productivity losses. Moreover, therapies approved via the AA pathway are more likely to treat more severe, rare diseases, and diseases disproportionately impacting children and the elderly. In the case the AA therapy provides improved clinical efficacy, delays in patient access to these therapies may worsen existing health inequities [16]. For children, treatment interruptions during critical developmental periods may have lifelong consequences, such as delays in access to gene therapy for spinal muscular atrophy (SMA), where earlier initiation is associated with improved motor function. For older adults, delays in therapies for diseases like early Alzheimer’s disease can accelerate functional decline, potentially resulting in increased reliance on informal caregivers and higher long-term care [17, 18].

Payers struggle to balance priorities between having certainty of clinical benefit and ensuring patients’ prompt access to novel therapies when making coverage decisions. In general, payers are also more likely to cover oncology therapies (approximately 99%) than non-oncology therapies (80%) [19]. This study, however, shows that the decision of delaying or denying access to non-oncology AA therapies would disproportionately impact patients with rare, serious diseases as well as the children and elderly. This should not be a surprise as the FDA uses the AA program for therapies that treat “serious conditions, and fill an unmet medical need” [3]. This regulatory framework reflects recent studies that have shown that individuals place greater value on health gains in more severe disease states [20]. Additionally, there are potential legal challenges for CMS and other payers failing to cover FDA-approved medications [21].

Payer policy must balance considerations of value, patient access, and innovation, and evaluating AA treatments deserves this same balance. Tension in payer policy related to AA therapies includes balancing rapid patient access in areas of high unmet need against the added costs and uncertainty in patient impact. Given additional evidence uncertainty at the time of the AA decision, commercial plan formulary decisions may include risk management strategies such as delayed or denied coverage, prior authorization, or step therapy. Yet the patients most affected by such restrictions, marked by high rates of rare disease designation, low QoL, and shortened life expectancy, tend to have the greatest clinical need and fewest alternatives. Payer decisions should consider that delayed or denied coverage of AA treatments disproportionately impacts patients with high unmet need and may result in long-term negative impacts for patients and their healthcare. While some payers claim that coverage with evidence development (CED) can help bridge the evidence gap from AA-approvals’ more limited evidence, some researchers have called CED “a never-ending coverage purgatory for innovators, with more than 30 complex steps required simply to establish a program.” Therefore, these findings should be considered alongside coverage decisions for AA therapies, which may disproportionately affect patients with severe disease and high unmet need.

This study has several limitations. First, the limited amount of data on indirect costs, particularly caregiver burden, may lead to an underestimation of the true societal value of these treatments. This study aimed to approximate this burden using algorithms developed in the literature Jiao and Basu, but disease specific estimates from survey data for all diseases would have been preferred. Second, while our analysis focused on unmet needs prior to the availability of AA treatments and did not account for the impact of AA, a small number of studies may have included timeframes both before and after the initial approval. All economic burden studies for AA diseases, however, were measured prior to the introduction of the AA therapy; thus any impact of the cost of the therapy itself would only serve to bias the findings to find no difference between AA and non-AA disease burden. Third, the sample size of this study for AA therapies was relatively small. Fourth, oncology indications were excluded from this analysis due to the current US payer policy application, yet the societal burden of AA oncology therapies could be an area for future investigation. Fifth, there were four diseases (i.e., Alzheimer's disease, amyotrophic lateral sclerosis (ALS), Duchenne muscular dystrophy, and sickle cell disease) which had both AA and non-AA therapies. Based on our classification rules, these diseases were defined as diseases with AA therapies. Finally, while insurance coverage for AA drugs is intended to improve patient access and outcomes, the extent to which this is achieved depends on the clinical effectiveness of these therapies, which this study does not consider. Therefore, the findings of this study are limited to illustrating the burden experienced by patients living with these conditions and the potential value of early access through accelerated approvals, rather than serving as definitive evidence of the benefit of extending coverage for any individual AA therapy.

## Conclusion

Before the availability of the treatment of interest, identified non-oncology AA-treated diseases imposed a numerically greater economic burden than diseases with treatments approved through traditional FDA pathways. Further, AA-treated diseases were also characterized by more severe disease characteristics, including lower QoL, shorter life expectancy, and disproportionately impacted children and older adults. Collectively, these findings suggest that coverage decisions for AA therapies warrant careful consideration, as they may disproportionately impact patients with severe diseases and high unmet needs.

## Supplementary Information

Below is the link to the electronic supplementary material.

Supplementary Material 1

## Data Availability

No datasets were generated or analysed during the current study.
