# Supplementary material for: Clinical and Economic Burden of Diseases with Accelerated Approval Therapies: Evidence of Unmet Patient Need
Source: Ther Innov Regul Sci. 2026 Aug 8;60(6):1840–7. doi: 10.1007/s43441-026-01030-x (PMC13722635; doi:10.1007/s43441-026-01030-x)
Supplement: Supplementary file 1 [file 43441_2026_1030_MOESM1_ESM.docx]

**Supplementary Information**

**Clinical and Economic Burden of Diseases Targeted by Accelerated Approval Therapies: Evidence of Unmet Patient Need**

Therapeutic Innovation & Regulatory Science

Hanke Zheng PhD ^a*^, Shanshan Wang MS ^b^, Jason Shafrin PhD ^b,c^, Shravani Bheema MS ^b^, Kathryn Spurrier BA ^b^, Jonathan D. Campbell PhD ^a^

^a^ National Pharmaceutical Council, ^b^ FTI Consulting, ^c^ University of Southern California

**Corresponding Author*

Hanke Zheng, PhD

[hzheng@npcnow.org](mailto:hzheng@npcnow.org)

**Supplementary Tables & Figures**

S1: Economic Burden of AA and non-AA diseases per year

| **Scenario** | **Category** | **Average Societal Costs** | **Average Direct Medical Costs** | **Average Direct Non-Medical Costs** | **Productivity Costs** | **Caregiver Burden** | **Average Direct Medical Costs (Excl Rx)** |
| --- | --- | --- | --- | --- | --- | --- | --- |
| **Original** | AAs | $91,857 | $64,878 | $16,184 | $17,635 | $20,268 | $51,250 |
|  | Non-AAs | $65,468 | $57,163 | $8,342 | $9,620 | $8,294 | $29,729 |
|  | Difference | $26,389 | $7,715 | $7,842 | $8,015 | $11,974 | $21,521 |
|  | % Difference | 40.30% | 13.50% | 94.00% | 83.30% | 144.40% | 72.40% |
| **Conservative** | % Difference | 40.30% | 13.50% | 127.60% | 168.90% | 421.30% | 72.40% |
| **Less Conservative** | % Difference | 31.30% | 13.50% | 55.90% | 54.40% | 129.00% | 72.40% |

S2: Economic burden by disease and cost type

|  |  |  | **Annual Costs (2025 USD)** | | | | | **Source: Direct - Medical** | **Source: Direct - Non-medical** | **Source: Indirect - Productivity** | **Source: Indirect - Caregiver** |
| --- | --- | --- | --- | --- | --- | --- | --- | --- | --- | --- | --- |
| **FDA Therapy Area** | **High Level Disease** | **AA** | **Total Societal** | **Direct - Medical** | **Direct- Medical (excluding Rx)** | **Indirect - Productivity** | **Indirect - Caregiver** |  |  |  |  |
| Endocrinology/Metabolism/Bone | Achondroplasia | Yes | $47,367 | $27,781 | $27,781 | $2,561 | $17,025 | [Broder et al. (2022)](https://pubmed.ncbi.nlm.nih.gov/36039778/) | N/A | [Maghnie et al (2023)](https://pmc.ncbi.nlm.nih.gov/articles/PMC10015810/) | [Due et al](https://www.ispor.org/docs/default-source/euro2024/isporeurope24duepcr79poster144279-pdf.pdf?sfvrsn=9ec25087_0) |
| Endocrinology/Metabolism/Bone | Acid Sphingomyelinase Deficiency | No | N/A | N/A | N/A | N/A | N/A | N/A | N/A | N/A | N/A |
| Hematology/Coagulation | Acquired thrombotic thrombocytopenic purpura (aTTP) | No | $92,609 | $92,609 | $92,609 | N/A | N/A | [Pollisard et al. (2021)](https://pubmed.ncbi.nlm.nih.gov/33904347/) | N/A | N/A | N/A |
| Dermatology | Actinic Keratosis | No | $232 | $232 | $232 | N/A | N/A | [Kirby et al. (2017)](https://www.ncbi.nlm.nih.gov/pmc/articles/PMC5470414/) | N/A | N/A | N/A |
| Immunomodulators | Activated phosphoinositide 3-kinase delta syndrome (APDS) | No | N/A | N/A | N/A | N/A | N/A | N/A | N/A | N/A | N/A |
| Hematology/Coagulation | Active anti-neutrophil cytoplasmic autoantibody-associated vasculitis (Granulomatosis with Polyangiitis) | No | $32,716 | $32,716 | $31,859 | N/A | N/A | [Raimundo et al. (2024)](https://www.jrheum.org/content/42/12/2383.long) | N/A | N/A | N/A |
| Hematology/Coagulation | Active anti-neutrophil cytoplasmic autoantibody-associated vasculitis (Microscopic Polyangiitis) | No | $152,392 | $152,392 | $144,565 | N/A | N/A | [Raimundo et al. (2015)](https://www.jrheum.org/content/42/12/2383.long) | N/A | N/A | N/A |
| Neurology | Acute Hepatic Porphyria (AHP) | No | $695,364 | $695,364 | $272,566 | N/A | N/A | [Ko et al. (2020)](https://www.valueinhealthjournal.com/article/S1098-3015(20)31447-9/fulltext) | N/A | N/A | N/A |
| Endocrinology/Metabolism/Bone | Alagille Syndrome (ALGS) | No | $62,568 | $62,568 | $55,138 | N/A | N/A | [Ebel et al. (2023)](https://www.sciencedirect.com/science/article/pii/S0022347622008551#:~:text=Utilizing%20insurance%20claims%20data%20to,stays%2C%20regardless%20of%20insurance%20type.) | N/A | N/A | N/A |
| Endocrinology/metabolism/Bone | Alpha-mannosidosis | No | N/A | N/A | N/A | N/A | N/A | [N/A](https://www.sciencedirect.com/science/article/pii/S0022347622008551#:~:text=Utilizing%20insurance%20claims%20data%20to,stays%2C%20regardless%20of%20insurance%20type.) | N/A | N/A | N/A |
| Neurology | Alzheimer's disease | Yes | $129,092 | $30,407 | $28,768 | $67,794 | $30,891 | [Alzheimer's Association (2023)](https://www.alz.org/media/documents/alzheimers-facts-and-figures.pdf) | N/A | [Fox et al (2024)](https://www.alz.org/media/documents/alzheimers-facts-and-figures.pdf) | [Alzheimer's Association (2023)](https://www.alz.org/media/documents/alzheimers-facts-and-figures.pdf) |
| Neurology | Amyotrophic Lateral Sclerosis (ALS) | Yes | $118,810 | $38,670 | $35,611 | $32,186 | $21,616 | [Larkindale et al. (2014)](https://onlinelibrary.wiley.com/doi/10.1002/mus.23942) | | [Thakore et al. (2020)](https://www.valueinhealthjournal.com/action/showPdf?pii=S1098-3015%2820%2934347-3) | [Larkindale et al. (2014)](https://onlinelibrary.wiley.com/doi/10.1002/mus.23942) |
| Dermatology | Atopic Dermatitis | No | $3,312 | $2,809 | $453 | $503 | N/A | [Manjelievskaia et al. (2021)](https://www.ncbi.nlm.nih.gov/pmc/articles/PMC10391209/) | N/A | [Manjelievskaia et al. (2021)](https://www.ncbi.nlm.nih.gov/pmc/articles/PMC10391209/) | N/A |
| Psychiatry | Attention Deficit Hyperactivity Disorder (ADHD)-- adults | No | $16,071 | $1,937 | $1,937 | $12,932 | $900 | [Schein et al (2021)](https://www.jmcp.org/doi/full/10.18553/jmcp.2021.21290) | | | |
| Psychiatry | Attention Deficit Hyperactivity Disorder (ADHD)-- children/adolescents | No | $9,238 | $2,468 | $2,468 | $368 | $1,133 | [Schein et al (2022)](https://www.tandfonline.com/doi/full/10.1080/13696998.2022.2032097) | | | |
| Hematology/Coagulation | Beta Thalassemia | No | $151,315 | $151,315 | $73,381 | N/A | N/A | [Weiss, Jun, and Sheth (2019)](https://onlinelibrary.wiley.com/doi/10.1002/ajh.25429) | N/A | N/A | N/A |
| Psychiatry | Bipolar I Disorder | No | $55,642 | $12,717 | $9,096 | $17,323 | $22,089 | [Cloutier et al. (2018)](https://www.sciencedirect.com/science/article/pii/S0165032717315100) | | | |
| Immunomodulators | CD55-deficient protein-losing enteropathy (PLE) | No | N/A | N/A | N/A | N/A | N/A | N/A | N/A | N/A | N/A |
| Infectious Disease (viral) | COVID-19 | No | $20,110 | $14,511 | $14,477 | $4,299 | $1,018 | [DeMartino et al. (2022)](https://www.jmcp.org/doi/full/10.18553/jmcp.2022.22050#F4) | [Gholipour et al. (2023)](https://www.ncbi.nlm.nih.gov/pmc/articles/PMC10511135/pdf/pone.0283651.pdf) | | |
| Infectious Disease (non viral) | Candidemia/Invasive Candidiasis | No | $65,659 | $61,881 | $61,881 | $3,778 | N/A | [Benedict, Whitman, and Jackson (2022)](https://www.ncbi.nlm.nih.gov/pmc/articles/PMC8946773/) | N/A | [Benedict, Whitman, and Jackson (2022)](https://www.ncbi.nlm.nih.gov/pmc/articles/PMC8946773/) | N/A |
| Infectious Disease (non viral) | Chagas disease | Yes | $23,463 | $4,880 | $4,880 | $18,583 | N/A | [Lee et al. (2013)](https://www.ncbi.nlm.nih.gov/pmc/articles/PMC3763184/) | N/A | [Lee et al. (2013)](https://www.ncbi.nlm.nih.gov/pmc/articles/PMC3763184/) | N/A |
| Gastroenterology | Chronic Idiopathic Constipation (CIC) | No | $6,850 | $5,165 | $4,184 | $1,685 | N/A | [Cai et al. (2013)](https://www.tandfonline.com/doi/full/10.3111/13696998.2013.860375) | N/A | [Heidelbaugh et al. (2015)](https://www.ncbi.nlm.nih.gov/pmc/articles/PMC4424385/pdf/ajg201567a.pdf) | N/A |
| Hematology/Coagulation | Cold Agglutinin Disease | No | N/A | N/A | N/A | N/A | N/A | N/A | N/A | N/A | N/A |
| Infectious Disease (non viral) | Community-acquired bacterial pneumonia (CABP) | No | $26,688 | $25,211 | $25,019 | $1,477 | N/A | [Divino et al. (2019)](https://www.tandfonline.com/doi/full/10.1080/03007995.2019.1675149) | N/A | [Broulette et al. (2013)](https://www.ncbi.nlm.nih.gov/pmc/articles/PMC4031734/#:~:text=Compared%20with%20individuals%20without%20CAP,depending%20on%20the%20risk%20level.) | N/A |
| Infectious Disease (non viral) | Complicated Urinary Tract Infections (cUTI) | No | $17,221 | $17,221 | $17,115 | N/A | N/A | [Lodise et al. (2022)](https://academic.oup.com/ofid/article/9/7/ofac307/6611843) | N/A | N/A | N/A |
| Infectious Disease (non viral) | Complicated intra-abdominal infections (cIAI) | No | $3,804 | $3,804 | $3,761 | N/A | N/A | [Prabhu et al. (2017)](https://aricjournal.biomedcentral.com/articles/10.1186/s13756-017-0264-2) | N/A | N/A | N/A |
| Endocrinology/Metabolism/Bone | Cushing's disease | No | $70,248 | $47,048 | $41,425 | N/A | N/A | [Burton et al. (2016)](https://www.ncbi.nlm.nih.gov/pmc/articles/PMC4799236/) | [Broder et al. (2015)](https://link.springer.com/article/10.1007/s11102-015-0654-5) | | |
| Pulmonary | Cystic Fibrosis (CF) | No | $102,768 | $98,474 | $53,312 | $1,854 | $2,439 | [Thorat et al. (2021)](https://www.ncbi.nlm.nih.gov/pmc/articles/PMC8456795/) | N/A | [Cheema et al. (2023)](https://www.cysticfibrosisjournal.com/article/S1569-1993(23)01722-8/fulltext#:~:text=39%20studies%20between%201990%20and,per%20year%20(2022%20US%24).) | [Daly et al. (2022)](https://www.ncbi.nlm.nih.gov/pmc/articles/PMC8958690/#:~:text=Suthoff%20et%20al.%209%20reported,study%20by%20Krivchenia%20et%20al.&text=cited%20inadequate%20income%20as%20a,Wojtaszcyzk%20et%20al.) |
| Ophthalmology | Demodex Blepharitis | No | N/A | N/A | N/A | N/A | N/A | N/A | N/A | N/A | N/A |
| Ophthalmology | Diabetic Macular Edema | No | $4,738 | $2,540 | $2,540 | $2,198 | N/A | [Mulligan et al. (2023)](https://diabetesjournals.org/care/article-abstract/46/6/1196/148674/The-Broader-Economic-Value-of-Treatment-for?redirectedFrom=fulltext) | N/A | [Mulligan et al. (2023)](https://diabetesjournals.org/care/article-abstract/46/6/1196/148674/The-Broader-Economic-Value-of-Treatment-for?redirectedFrom=fulltext) | N/A |
| Ophthalmology | Dry Eye Disease | No | $18,984 | $1,230 | $1,230 | $17,754 | N/A | [Yu et al. (2011)](https://www.sciencedirect.com/science/article/pii/S1542012415002049?via%3Dihub) | N/A | N/A | N/A |
| Neurology | Duchenne Muscular Dystrophy (DMD) | Yes, verified | $104,504 | $34,954 | $32,101 | $29,703 | $18,428 | [Landfeldt et al. (2014)](https://www.ncbi.nlm.nih.gov/pmc/articles/PMC4141999/) | | | |
| Neurology | Fabry Disease | Yes | $186,499 | $185,425 | $11,837 | $1,074 | N/A | [Santamaria et al](https://academic.oup.com/ndt/article/34/Supplement_1/gfz106.FP136/5514919) | | | |
| Infectious Disease (non viral) | Fascioliasis | No | N/A | N/A | N/A | N/A | N/A | N/A | N/A | N/A | N/A |
| Neurology | Friedrich's Ataxia | No | $30,440 | $26,921 | $25,449 | N/A | N/A | [Polek et al. (2013)](https://pubmed.ncbi.nlm.nih.gov/23734128/) | | N/A | N/A |
| Endocrinology/Metabolism/Bone | Growth Hormone Deficiency | No | $29,686 | $29,686 | $5,213 | N/A | N/A | [Kaplowitz et al. (2021)](https://pubmed.ncbi.nlm.nih.gov/33896224/) | N/A | N/A | N/A |
| Infectious Disease (viral) | HIV-1 infection | No | $35,889 | $35,889 | $10,098 | N/A | N/A | [Kangethe et al. (2019)](https://www.ncbi.nlm.nih.gov/pmc/articles/PMC10398135/pdf/jmcp-025-01-088.pdf) | N/A | N/A | N/A |
| Cardiovascular Disease | Heart Failure | No | $33,621 | $30,468 | $30,468 | $1,522 | $1,631 | [Urbich et al. (2020)](https://www.ncbi.nlm.nih.gov/pmc/articles/PMC7546989/) | N/A | [Heindenreich et al. (2013)](https://www.ahajournals.org/doi/epub/10.1161/HHF.0b013e318291329a) | [Dunbar et al. (2018)](https://www.ahajournals.org/doi/full/10.1161/CIR.0000000000000570) |
| Infectious Disease (non viral) | Helicobacter Pylori Infection | No | $26,100 | $22,531 | $19,365 | $3,569 | N/A | [Shah et al. (2023)](https://www.tandfonline.com/doi/full/10.1080/13696998.2023.2263252) | N/A | [Gurusamy et al. (2016)](https://pubmed.ncbi.nlm.nih.gov/27025289/) | N/A |
| Hematology/Coagulation | Hemophilia A | No | $770,304 | $739,842 | $20,242 | $30,461 | N/A | [Chen et al. (2023)](https://www.ncbi.nlm.nih.gov/pmc/articles/PMC10387983/pdf/jmcp.2023.29.6.647.pdf) | N/A | [Chen et al. (2023)](https://www.ncbi.nlm.nih.gov/pmc/articles/PMC10387983/pdf/jmcp.2023.29.6.647.pdf) | |
| Hematology/Coagulation | Hemophilia B | No | $241,491 | $231,363 | $55,861 | $7,546 | $674 | [Buckner et al. (2021)](https://www.ncbi.nlm.nih.gov/pmc/articles/PMC8045501/pdf/advancesADV2020003424.pdf) | [Burke et al. (2021)](https://www.ncbi.nlm.nih.gov/pmc/articles/PMC7981988/pdf/13023_2021_Article_1774.pdf) | | |
| Neurology | Hereditary Transthyretin-Mediated Amyloidosis | No | $77,083 | $77,083 | $70,162 | N/A | N/A | [Reddy et al. (2020)](https://link.springer.com/article/10.1007/s40120-020-00194-4) | N/A | N/A | N/A |
| Allergy | Hereditary angioedema (HAE) | No | $80,557 | $42,961 | $35,313 | $22,049 | $9,004 | [Lumry (2018)](https://www.frontiersin.org/articles/10.3389/fmed.2018.00022/full) | | | [Castaldo et al. (2021)](https://www.ncbi.nlm.nih.gov/pmc/articles/PMC8133018/) |
| Infectious Disease (non viral) | Hospital-Acquired Bacterial Pneumonia | No | $74,916 | $74,916 | $74,916 | N/A | N/A | [Tabak et al. (2019)](https://www.sciencedirect.com/science/article/abs/pii/S0195670119302592) | N/A | N/A | N/A |
| Infectious Disease (non viral) | Human African trypanosomiasis (HAT) | No | N/A | N/A | N/A | N/A | N/A | N/A | N/A | N/A | N/A |
| Endocrinology/Metabolism/Bone | Hutchinson-Gilford Progeria Syndrome and premature aging | No | N/A | N/A | N/A | N/A | N/A | N/A | N/A | N/A | N/A |
| Endocrinology/Metabolism/Bone | Hypercholesterolemia | No | $59,035 | $57,804 | $52,719 | N/A | $1,231 | [Henk et al. (2015)](https://www.ncbi.nlm.nih.gov/pmc/articles/PMC4662727/pdf/12325_2015_Article_264.pdf) | N/A | N/A | [Joo et al. (2015)](https://www.ncbi.nlm.nih.gov/pmc/articles/PMC4392718/pdf/nihms676728.pdf) |
| Renal Disease | Hyperoxaluria type 1 | No | $23,404 | $23,404 | $21,498 | N/A | N/A | [Mucha et al. (2022)](https://pubmed.ncbi.nlm.nih.gov/35199581/) | N/A | N/A | N/A |
| Psychiatry | Hypoactive sexual desire disorder (HSDD) | No | $1,662 | $1,662 | $1,367 | N/A | N/A | [Thurston et al. (2022)](https://www.ncbi.nlm.nih.gov/pmc/articles/PMC9525110/) | N/A | N/A | N/A |
| Neurology | Insomnia | No | $10,359 | $7,002 | $5,927 | $3,357 | N/A | [Anderson et al. (2014)](https://www.ajmc.com/view/healthcare-utilization-and-costs-in-persons-with-insomnia-in-a-managed-care-population) | N/A | [Taddei-Allen (2020)](https://www.ajmc.com/view/economic-burden-and-managed-care-considerations-for-the-treatment-of-insomnia) | N/A |
| Hematology/Coagulation | Iron deficiency anemia | No | $11,760 | $11,760 | $5,182 | N/A | N/A | [Blaney et al. (2020)](https://link.springer.com/article/10.1007/s10620-020-06590-y) | N/A | N/A | N/A |
| Gastroenterology | Irritable bowel syndrome with constipation (IBS-C) | No | $11,105 | $5,677 | $4,435 | $5,428 | N/A | [Doshi et al. (2014)](https://www.ncbi.nlm.nih.gov/pmc/articles/PMC10437385/pdf/jmcp.2014.20.4.382.pdf) | N/A | [Buono et al. (2014)](https://www.ncbi.nlm.nih.gov/pmc/articles/PMC4163780/pdf/ahdb-07-289.pdf) | N/A |
| Endocrinology/Metabolism/Bone | Long-chain fatty acid oxidation disorders (LC-FAOD) | No | N/A | N/A | N/A | N/A | N/A | N/A | N/A | N/A | N/A |
| Immunomodulators | Lupus nephritis (LN) | No | $51,399 | $28,358 | $24,956 | $23,041 | N/A | [Bell et al. (2023)](https://www.ncbi.nlm.nih.gov/pmc/articles/PMC10198302/pdf/cureus-0015-00000037839.pdf) | N/A | [Nee et al. (2015)](https://pubmed.ncbi.nlm.nih.gov/26600951/) | |
| Psychiatry | Major Depressive Disorder (MDD) | No | $17,554 | $6,696 | $5,291 | $6,642 | $4,216 | [Greenberg et al. (2023); Greenberg et al. (2021)](https://link.springer.com/article/10.1007/s12325-023-02622-x#:~:text=The%20incremental%20costs%20of%20all,MDD%3B%20Supplementary%20Table%20S7).) | N/A | [Greenberg et al. (2023); Greenberg et al. (2021)](https://link.springer.com/article/10.1007/s12325-023-02622-x#:~:text=The%20incremental%20costs%20of%20all,MDD%3B%20Supplementary%20Table%20S7).) | |
| Neurology | Migraine | No | $11,726 | $8,638 | $6,365 | $3,087 | N/A | [Bonafede et al. (2018)](https://pubmed.ncbi.nlm.nih.gov/29446063/) | N/A | [Bonafede et al. (2018)](https://pubmed.ncbi.nlm.nih.gov/29446063/) | N/A |
| Neurology | Multiple Sclerosis (MS) | No | $101,535 | $75,287 | $30,196 | $19,973 | $4,972 | [Bebo et al. (2022)](https://www.ncbi.nlm.nih.gov/pmc/articles/PMC9109149/pdf/WNL-2022-200403.pdf) | | | |
| Neurology | Myasthenia Gravis | No | $30,753 | $22,076 | $20,223 | $470 | $8,207 | [Phillips et al. (2022)](https://www.ncbi.nlm.nih.gov/pmc/articles/PMC8805609/pdf/fneur-12-809999.pdf) | N/A | [Dewilde et al. (2023)](https://www.ncbi.nlm.nih.gov/pmc/articles/PMC10499690/) | |
| Neurology | Narcolepsy | No | $44,854 | $8,665 | $5,649 | $9,731 | $26,458 | [Black et al. (2014)](https://www.sciencedirect.com/science/article/abs/pii/S1389945714000665) | N/A | [Flores et al. (2016)](https://www.ncbi.nlm.nih.gov/pmc/articles/PMC4773621/pdf/jcsm.12.3.401.pdf) | [Hussain et al. (2019)](https://www.pharllc.com/wp-content/uploads/2020/03/Hussain-Epilepsia-2020.pdf) |
| Neurology | Neurofibromatosis type 1 (NF1) | No | $33,509 | $30,520 | $26,249 | N/A | $2,989 | [Yang et al. (2020)](https://www.ncbi.nlm.nih.gov/pmc/articles/PMC7548319/pdf/phmt-11-421.pdf) | N/A | N/A | [Yang et al. (2022)](https://link.springer.com/article/10.1007/s40120-022-00365-5) |
| Neurology | Neuromyelitis Optica Spectrum Disorder | No | $62,437 | $62,437 | $56,965 | N/A | N/A | [Exuzides et al. (2021)](https://www.jns-journal.com/article/S0022-510X(21)00247-1/fulltext) | N/A | N/A | N/A |
| Hematology/Coagulation | Neutropenia | No | $27,797 | $27,797 | $21,728 | N/A | N/A | [Dulisse et al. (2013)](https://www.tandfonline.com/doi/epdf/10.3111/13696998.2013.782034?needAccess=true) | N/A | N/A | N/A |
| Cardiovascular Disease | Obstructive Hypertrophic Cardiomyopathy | No | $23,088 | $23,088 | $21,395 | N/A | N/A | [Jain et al. (2021)](https://www.tandfonline.com/doi/full/10.1080/13696998.2021.1978242) | N/A | N/A | N/A |
| Neurology | Obstructive Sleep Apnea (OSA) | No | $7,428 | $2,695 | $2,695 | $4,734 | N/A | [Frost & Sullivan (2015)](https://aasm.org/wp-content/uploads/2017/10/sleep-apnea-economic-crisis.pdf) | N/A | [Frost & Sullivan (2015)](https://aasm.org/wp-content/uploads/2017/10/sleep-apnea-economic-crisis.pdf) | N/A |
| Endocrinology/Metabolism/Bone | Osteoporosis | No | $32,771 | $23,426 | $23,258 | $6,562 | $2,783 | [Williams et al. (2020)](https://www.ajmc.com/view/economic-burden-of-osteoporotic-fractures-in-us-managed-care-enrollees) | N/A | [Lewiecki et al. (2019)](https://www.ncbi.nlm.nih.gov/pmc/articles/PMC6808223/pdf/JBM4-3-na.pdf) | [Tran et al. (2022)](https://link.springer.com/article/10.1007/s00198-020-05769-3#change-history) |
| Urologic | Overactive bladder (OAB) | No | $27,472 | $13,632 | $10,498 | $13,733 | N/A | [Powell et al. (2018)](https://onlinelibrary.wiley.com/doi/abs/10.1002/nau.23477) | [Coyne et al. (2014)](https://www.ncbi.nlm.nih.gov/pmc/articles/PMC10437639/pdf/jmcp.2014.20.2.130.pdf) | [Powell et al. (2018)](https://onlinelibrary.wiley.com/doi/abs/10.1002/nau.23477) | [Powell et al. (2018)](https://onlinelibrary.wiley.com/doi/abs/10.1002/nau.23477) |
| Neurology | Parkinson's disease (PD) | No | $60,153 | $29,403 | $24,605 | $13,121 | $8,888 | [Yang et al. (2020)](https://www.ncbi.nlm.nih.gov/pmc/articles/PMC7347582/pdf/41531_2020_Article_117.pdf) | [Yang et al. (2020)](https://www.ncbi.nlm.nih.gov/pmc/articles/PMC7347582/pdf/41531_2020_Article_117.pdf) | [Yang et al. (2020)](https://www.ncbi.nlm.nih.gov/pmc/articles/PMC7347582/pdf/41531_2020_Article_117.pdf) | [Yang et al. (2020)](https://www.ncbi.nlm.nih.gov/pmc/articles/PMC7347582/pdf/41531_2020_Article_117.pdf) |
| Hematology/Coagulation | Paroxysmal Nocturnal Hemoglobinuria (PNH) | No | $634,443 | $625,998 | $52,753 | $8,444 | N/A | [Clayton et al. (2024)](https://journals.sagepub.com/doi/10.1177/10760296231213073) | N/A | [Clayton et al. (2024)](https://journals.sagepub.com/doi/10.1177/10760296231213073) | N/A |
| Neurology | Partial onset seizures (epilepsy) | No | $32,318 | $22,567 | $15,044 | $2,181 | $7,570 | [Prescott et al. (2020)](https://cdn.sanity.io/files/0vv8moc6/ajmc/e733afad09f872a8c10470c6fa19feb24edb6cf4.pdf/A948_ClinicalBrief_pdf.pdf) | N/A | [Brook et al. (2018)](https://pubmed.ncbi.nlm.nih.gov/30647827/) | [Brook et al. (2018)](https://pubmed.ncbi.nlm.nih.gov/30647827/) |
| Dermatology | Plaque psoriasis | No | $6,300 | $3,806 | $1,110 | $548 | N/A | [Pilon et al. (2019)](https://www.tandfonline.com/doi/epdf/10.1080/13696998.2018.1557201?needAccess=true) | [Schaefer et al. (2015)](https://www.jaad.org/action/showPdf?pii=S0190-9622%2815%2901855-1) | [Pilon et al. (2019)](https://www.tandfonline.com/doi/epdf/10.1080/13696998.2018.1557201?needAccess=true) | N/A |
| Endocrinology/metabolism/Bone | Pompe disease | No | $66,024 | $62,435 | $62,435 | N/A | $3,590 | [Lewin Group (2023)](https://everylifefoundation.org/wp-content/uploads/2023/09/EveryLife-Cost-of-Delayed-Diagnosis-in-Rare-Disease_Final-Full-Study-Report_0914223.pdf) | N/A | N/A | [Lewin Group (2023)](https://everylifefoundation.org/wp-content/uploads/2023/09/EveryLife-Cost-of-Delayed-Diagnosis-in-Rare-Disease_Final-Full-Study-Report_0914223.pdf) |
| Infectious Disease (viral) | Post-transplant cytomegalovirus (CMV) | No | $68,013 | $68,013 | $51,294 | N/A | N/A | [Cheng et al. (2022)](https://www.tandfonline.com/doi/epdf/10.1080/13696998.2022.2046388?needAccess=true) | N/A | N/A | N/A |
| Psychiatry | Postpartum Depression (PPD) | No | $11,719 | $7,196 | $7,196 | $4,523 | N/A | [Eldar-Lissai et al. (2020)](https://www.jmcp.org/doi/full/10.18553/jmcp.2020.19306) | N/A | [Luca et al. (2020)](https://www.ncbi.nlm.nih.gov/pmc/articles/PMC7204436/#:~:text=The%20average%20cost%20per%20affected,in%20other%20maternal%20health%20expenditures.) | N/A |
| Dermatology | Pustular Psoriasis | No | $37,755 | $36,613 | $13,618 | $1,143 | N/A | [Hanna et al. (2021)](https://www.tandfonline.com/doi/full/10.1080/03007995.2021.1894108) | N/A | [Hanna et al. (2020)](https://www.valueinhealthjournal.com/action/showPdf?pii=S1098-3015%2820%2934033-X) | N/A |
| Neurology | Rett Syndrome | No | $19,674 | $19,674 | $15,942 | N/A | N/A | [May et al. (2023)](https://pubmed.ncbi.nlm.nih.gov/37016355/) | N/A | N/A | N/A |
| Rheumatology | Rheumatoid arthritis | No | $39,542 | $38,769 | $8,855 | $773 | N/A | [Baser et al. (2015)](https://www.clinicaltherapeutics.com/article/S0149-2918(15)00253-2/fulltext) | N/A | [Kleinman et al. (2013)](https://pubmed.ncbi.nlm.nih.gov/23471034/) | N/A |
| Psychiatry | Schizophrenia | No | $79,697 | $18,331 | $14,776 | $18,096 | $32,989 | [Kadakia et al. (2022)](https://www.psychiatrist.com/read-pdf/43278/) | [Kadakia et al. (2022)](https://www.psychiatrist.com/read-pdf/43278/) | [Kadakia et al. (2022)](https://www.psychiatrist.com/read-pdf/43278/) | [Kadakia et al. (2022)](https://www.psychiatrist.com/read-pdf/43278/) |
| Endocrinology/metabolism/Bone | Hypoglycemia | No | $118 | $38 | $38 | $80 | N/A | [Foos et al. (2015)](https://www.tandfonline.com/doi/epdf/10.3111/13696998.2015.1006730?needAccess=true) | N/A | [Foos et al. (2015)](https://www.tandfonline.com/doi/epdf/10.3111/13696998.2015.1006730?needAccess=true) | N/A |
| Infectious Disease (non viral) | Malaria | No | $58,090 | $58,090 | $57,988 | N/A | N/A | [Park et al. (2023)](https://academic.oup.com/jtm/article/30/3/taad013/7015603) | N/A | N/A | N/A |
| Immunomodulators | Severe systemic lupus erythematosus (SLE) | No | $75,130 | $66,389 | $53,218 | $8,740 | N/A | [Lin et al. (2022)](https://journals.sagepub.com/doi/epub/10.1177/09612033221088209) | N/A | [Lin et al. (2022)](https://journals.sagepub.com/doi/epub/10.1177/09612033221088209) | N/A |
| Hematology/Coagulation | Sickle Cell Disease | Yes | $70,359 | $30,306 | $30,306 | $18,646 | $21,407 | [Huo et al. (2018)](https://www.valueinhealthjournal.com/article/S1098-3015(18)33183-8/fulltext#%20) | N/A | [Holdford et al. (2021)](https://pubmed.ncbi.nlm.nih.gov/34372974/) | [Holdford et al. (2021)](https://pubmed.ncbi.nlm.nih.gov/34372974/) |
| Neurology | Spinal muscular atrophy (SMA) | No | $193,977 | $156,319 | $154,831 | N/A | $37,659 | [Droege et al. (2020)](https://www.tandfonline.com/doi/full/10.1080/13696998.2019.1646263) | N/A | N/A | [Jimenez et al. (2022)](https://www.neurology.org/doi/10.1212/WNL.98.18_supplement.1160) |
| Endocrinology/metabolism/Bone | Tenosynovial giant cell tumor (TGCT) | No | $14,063 | $10,868 | $9,021 | $3,195 | N/A | [Lin et al. (2021)](https://pubmed.ncbi.nlm.nih.gov/33560066/) | N/A | [Lin et al. (2021)](https://pubmed.ncbi.nlm.nih.gov/33560066/) | N/A |
| Dermatology | Thermal Burns | No | $80,788 | $80,788 | $80,788 | N/A | N/A | [Yu et al. (2020)](https://www.sciencedirect.com/science/article/pii/S0305417919304899?via%3Dihub) | N/A | N/A | N/A |
| Ophthalmology | Thyroid eye disease | No | $11,356 | $11,356 | $11,356 | N/A | N/A | [Singh et al. (2021)](https://www.ncbi.nlm.nih.gov/pmc/articles/PMC8090281/pdf/bvab048.1720.pdf) | N/A | N/A | N/A |
| Endocrinology/metabolism/Bone | Type 1 Diabetes | No | $26,393 | $10,238 | $4,946 | $14,362 | $1,793 | [Simeone et al. (2020)](https://www.ncbi.nlm.nih.gov/pmc/articles/PMC10390920/pdf/jmcp.2020.26.11.1399.pdf) | N/A | [Health Advances (2020)](https://t1dfund.org/wp-content/uploads/2020/02/Health-Advances-T1D-Concept-Value-White-Paper-2020.pdf?_ga=2.65942698.587281256.1704754286-1922756637.1704754286) | [Health Advances (2020)](https://t1dfund.org/wp-content/uploads/2020/02/Health-Advances-T1D-Concept-Value-White-Paper-2020.pdf?_ga=2.65942698.587281256.1704754286-1922756637.1704754286) |
| Gastroenterology | Ulcerative Colitis | No | $17,414 | $14,465 | $10,372 | $2,949 | N/A | [Cohen et al. (2015)](https://www.tandfonline.com/doi/epdf/10.3111/13696998.2015.1021353?needAccess=true) | N/A | [Cohen et al. (2015)](https://www.tandfonline.com/doi/epdf/10.3111/13696998.2015.1021353?needAccess=true) | N/A |
| Ophthalmology | Wet Age-Related Macular Degeneration (AMD) | No | $54,235 | $8,068 | $8,068 | $46,167 | N/A | [Almony et al. (2021)](https://pubmed.ncbi.nlm.nih.gov/34464210/) | N/A | [Retina International (2022)](https://retina-international.org/wp-content/uploads/2022/10/AMD-Economic-assessment-Final-06102022.pdf) | N/A |
| Hematology/Coagulation | Niemann-Pick Disease | No | $241,599 | $111,569 | $111,569 | $71,791 | N/A | [#N/A](https://www.tandfonline.com/doi/full/10.3111/13696990903245863) | [Imrie et al (2009)](https://www.tandfonline.com/doi/full/10.3111/13696990903245863) | [Imrie et al (2009)](https://www.tandfonline.com/doi/full/10.3111/13696990903245863) | N/A |
| Immunomodulators | WHIM Syndrome | No | $28,196 | $28,196 | $28,196 | N/A | N/A | [#N/A](https://www.reuters.com/business/healthcare-pharmaceuticals/us-fda-approves-x4-pharmaceuticals-drug-immunodeficiency-disease-2024-04-29/) | N/A | N/A | N/A |
| Cardiovascular Disease | Supraventricular Tachycardia | No | $8,378 | $8,378 | $8,378 | N/A | N/A | [#N/A](https://www.sciencedirect.com/science/article/abs/pii/S0002870320304142) | N/A | N/A | N/A |
| Cardiovascular Disease | Pulmonary Arterial Hypertension | No | $99,021 | $99,021 | $16,298 | N/A | N/A | [#N/A](https://link.springer.com/article/10.1007/s40273-024-01427-2) | N/A | N/A | N/A |
| Dermatology | Prurigo Nodularis | No | $8,527 | $8,527 | $6,231 | N/A | N/A | [#N/A](https://link.springer.com/article/10.1007/s13555-025-01349-7) | N/A | N/A | N/A |
| Gastroenterology | Primary Biliary Cholangitis (PBC) | Yes | $41,207 | $41,207 | $39,656 | N/A | N/A | #N/A | N/A | N/A | N/A |
| Endocrinology/Metabolism/Bone | Familial Chylomicronemia Syndrome (FCS) | No | $1,203 | $1,231 | $1,068 | N/A | N/A | [#N/A](https://www.tandfonline.com/doi/pdf/10.3111/13696998.2013.779277) | N/A | N/A | N/A |
| Cardiovascular Disease | Transthyretin Mediated Amyloidosis - Cardiomyopathy (ATTR-CM) | No | $53,068 | $53,068 | $53,068 | N/A | N/A | [#N/A](https://ojrd.biomedcentral.com/articles/10.1186/s13023-022-02425-3) | N/A | N/A | N/A |
| Dermatology | Alopecia Areata (AA) | No | $5,488 | $5,488 | $4,970 | N/A | N/A | [#N/A](https://www.jmcp.org/doi/10.18553/jmcp.2022.28.4.426) | N/A | N/A | N/A |
| Endocrinology/Metabolism/Bone | Noncirrhotic Non-Alcoholic Steatohepatitis (NASH) | Yes | $25,499 | $17,649 | $16,208 | $6,324 | $733 | [#N/A](https://becarispublishing.com/doi/10.57264/cer-2024-0096) | [O'Hara et al](https://www.sciencedirect.com/science/article/pii/S2589555920300768#appsec1) | [O'Hara et al](https://www.sciencedirect.com/science/article/pii/S2589555920300768#appsec1) | [O'Hara et al](https://www.sciencedirect.com/science/article/pii/S2589555920300768#appsec1) |
| Cardiovascular Disease | Hypertension | No | $3,775 | $3,166 | $2,696 | $381 | $227 | [#N/A](https://pmc.ncbi.nlm.nih.gov/articles/PMC11585433/) | N/A | [Asay et al](https://pmc.ncbi.nlm.nih.gov/articles/PMC5055401/#T1FN1) | [Dunbar et al](https://www.ahajournals.org/doi/10.1161/CIR.0000000000000570) |
| Endocrinology/Metabolism/Bone | Classic Congenital Adrenal Hyperplasia (CAH) | No | $4,447 | $4,447 | $4,447 | N/A | N/A | #N/A | N/A | N/A | N/A |
| Pulmonary | Chronic Obstructive Pulmonary Disease (COPD) | No | $5,810 | $5,099 | $2,873 | $711 | N/A | [#N/A](https://link.springer.com/article/10.1007/s40258-023-00814-8) | N/A | [Patel et al](https://www.dovepress.com/article/download/39557) | N/A |
| Dermatology | Molluscum Contagiosum | No | N/A | N/A | N/A | N/A | N/A | #N/A | N/A | N/A | N/A |
| Urologic | Uncomplicated Urinary Tract Infections (uUTI) | No | $313 | $313 | $301 | N/A | N/A | [#N/A](https://pmc.ncbi.nlm.nih.gov/articles/PMC10677141/) | N/A | N/A | N/A |
| Hematology/Coagulation | Chronic graft-versus-host disease (cGVHD) | No | $44,507 | $44,507 | $44,507 | N/A | N/A | [#N/A](https://www.sciencedirect.com/science/article/pii/S2666636724006973) | N/A | N/A | N/A |
| Endocrinology/Metabolism/Bone | Hypoparathyroidism | No | $6,929 | $6,929 | $6,929 | N/A | N/A | [#N/A](https://pmc.ncbi.nlm.nih.gov/articles/PMC10157763/) | N/A | N/A | N/A |
| Hematology | Methemoglobinemia | Yes, verified | N/A | N/A | N/A | N/A | N/A | #N/A | N/A | N/A | N/A |
| Endocrinology/Metabolism/Bone | Aromatic L-amino acid decarboxylase (AADC) deficiency | Yes | $74,488 | $27,415 | $19,148 | N/A | $47,073 | [#N/A](https://onlinelibrary.wiley.com/doi/full/10.1002/cns3.20010) | N/A | N/A | [####](https://onlinelibrary.wiley.com/doi/full/10.1002/cns3.20010) |
| Pulmonary | Mycobacterium Avium Complex (MAC) Lung Disease | Yes | $29,267 | $25,705 | $24,764 | $3,562 | N/A | [#N/A](https://wwwnc.cdc.gov/eid/article/26/9/19-0524_article) | N/A | [Diel et al](https://publications.ersnet.org/content/erj/49/4/1602109) | N/A |
| Neurology | Cerebral adrenoleukodystrophy (CALD) | Yes | $150,306 | $150,306 | $150,306 | N/A | N/A | [#N/A](https://www.valueinhealthjournal.com/article/S1098-3015(20)31129-3/pdf) | N/A | N/A | N/A |
| Renal Disease | Primary Immunoglobulin A nephropathy (IgAN) | Yes, verified | $50,430 | $36,572 | $25,496 | $8,887 | $4,971 | [#N/A](https://www.sciencedirect.com/science/article/pii/S2590059523001115) | N/A | [Bensink et al](https://academic.oup.com/ndt/article/39/Supplement_1/gfae069-1343-366/7677607?login=false) | [Bensink et al](https://academic.oup.com/ndt/article/39/Supplement_1/gfae069-1343-366/7677607?login=false) |
| Hematology/Coagulation | Uncontrolled Bleeding | Yes | $8,801 | $8,801 | $8,801 | N/A | N/A | [#N/A](https://pmc.ncbi.nlm.nih.gov/articles/PMC10327677/) | N/A | N/A | N/A |
| Hematology/Coagulation | Primary Hemophagocytic Lymphohistiocytosis (pHLH) | No | $62,994 | $62,994 | $62,994 | N/A | N/A | [#N/A](https://www.thelancet.com/journals/eclinm/article/PIIS2589-5370(23)00320-6/fulltext) | N/A | N/A | N/A |
| Infectious Disease (non viral) | Travelers' diarrhea | No | $181 | $181 | $150 | N/A | N/A | [#N/A](https://pmc.ncbi.nlm.nih.gov/articles/PMC10941978/) | $ - | 0 | $- |
| Neurology | Lambert-Eaton myasthenic syndrome (LEMS) | No | N/A | N/A | N/A | N/A | N/A | #N/A | N/A | N/A | N/A |
| Dermatology | Primary axillary hyperhidrosis | No | $1,289 | $1,289 | $336 | N/A | N/A | [#N/A](https://www.tandfonline.com/doi/full/10.1080/13696998.2020.1855879#d1e309) | N/A | N/A | N/A |
| Hematology/Coagulation | Light Chain Amyloidosis | Yes | $317,757 | $313,090 | $313,090 | $4,667 | N/A | #N/A | N/A | [Rizio et al](https://pmc.ncbi.nlm.nih.gov/articles/PMC8685766/) | N/A |

S3: Disease characteristics of AA vs non-AA diseases

| **Category** | **Average Age at Onset** | **Percentage < 18 years** | **Percentage > 65 years** | **Life Expectancy** | **Life Years (LY) after Diagnosis** | **QoL while Alive** | **Prevalence (per 100,000)** | **Disease Duration (Years)** | **Orphan Designation** |
| --- | --- | --- | --- | --- | --- | --- | --- | --- | --- |
| AA | 33 | 32% | 29% | 59.3 | 25 | 0.64 | 312 | 18.9 | 75% |
| Non-AA | 31.5 | 24% | 23% | 65.1 | 31 | 0.69 | 2,250 | 21.9 | 50% |
| Difference | 1.5 | 8% | 6% | -5.7 | -6 | -0.05 | -1,939 | -3 | 25% |
| % Difference | 5% | 32% | 24% | -9% | -19% | -7% | -86% | -14% | 50% |

S4: Histogram of distribution disease characteristics (Average Age of Onset and Life Expectancy)

S5: Histogram of distribution disease characteristics (Life Years After Diagnosis and QoL)

S6: Characteristics for individual diseases

| **Therapeutic Area** | **Disease Name** | **AA** | **Average Age at Onset** | **% < 18 years** | | **% > 65 years** | **LE** | **(LY) after Diagnosis** | **QoL** | **Prevalence (per 100,000)** | **Orphan Designation** | | **Ref: Onset** | **Ref: % < 18** | | | **Ref:**  **% > 65** | | **Ref: LE** | | **Ref: LY** | **Ref: QoL** | | **Ref: Prevalence** | | **Ref: Orphan Designation** |
| --- | --- | --- | --- | --- | --- | --- | --- | --- | --- | --- | --- | --- | --- | --- | --- | --- | --- | --- | --- | --- | --- | --- | --- | --- | --- | --- |
| Immunomodulators | Activated Phosphoinositide 3-kinase Delta Syndrome (APDS) | No | 3.7 | 67.97% | 0% | | 57.0 | 53.3 | N/A | 0.2 | Yes | [Oh et al. (2021)](https://www.ncbi.nlm.nih.gov/pmc/articles/PMC8578310/pdf/nihms-1733803.pdf) | | | [Hanson et al. (2022)](https://www.medrxiv.org/content/medrxiv/early/2022/12/06/2022.12.05.22283110.full.pdf) | [Hanson et al. (2022)](https://www.medrxiv.org/content/medrxiv/early/2022/12/06/2022.12.05.22283110.full.pdf) | | [Hanson et al. (2022)](https://www.medrxiv.org/content/medrxiv/early/2022/12/06/2022.12.05.22283110.full.pdf) | | Assumption | | N/A | [Vanselow, Wahn, Schuetz (2023)](https://www.ncbi.nlm.nih.gov/pmc/articles/PMC10432830/#:~:text=APDS%20is%20a%20very%20rare%20disease%20with%20an%20estimated%20prevalence,2%20affected%20people%20per%20million.) | | [FDA (2023)](https://www.accessdata.fda.gov/scripts/cder/daf/index.cfm?event=overview.process&varApplNo=217759) | |
| Immunomodulators | CD55-deficient Protein-losing Enteropathy (PLE) | No | 0.5 | 100% | 0% | | N/A | N/A | N/A | 0.0 | Yes | [Ernst (2023)](https://www.empr.com/home/news/fda-greenlights-veopoz-the-first-treatment-for-chaple-disease/#:~:text=CHAPLE%20disease%20is%20a%20rare,recurrent%20infections%2C%20and%20blood%20clots.) | | | [CHAPLE](https://www.whatischaple.com/) | [CHAPLE](https://www.whatischaple.com/) | | N/A | | N/A | | N/A | [Healio (2023)](https://www.healio.com/news/gastroenterology/20230818/fda-approves-veopoz-for-children-and-adults-with-chaple-disease#:~:text=It%20is%20the%20first%2Dever,mutations%20in%20their%20CD55%20gene.) | | [FDA (2023)](https://www.accessdata.fda.gov/scripts/cder/daf/index.cfm?event=overview.process&varApplNo=761339) | |
| Infectious Disease (non viral) | Fascioliasis | No | N/A | N/A | N/A | | N/A | N/A | N/A | N/A | N/A | N/A | | | N/A | N/A | | N/A | | N/A | | N/A | N/A | | N/A | |
| Infectious Disease (non viral) | Human African Trypanosomiasis (HAT) | No | N/A | N/A | N/A | | N/A | N/A | N/A | N/A | N/A | N/A | | | N/A | N/A | | N/A | | N/A | | N/A | N/A | | N/A | |
| Endocrinology/Metabolism/Bone | Hutchinson-Gilford Progeria Syndrome and premature aging | No | 0 | 100% | 0% | | 14.0 | 14.0 | N/A | 0.0 | Yes | [Pachajoa et al. (2020)](https://www.ncbi.nlm.nih.gov/pmc/articles/PMC7481268/) | | | [Pachajoa et al. (2020)](https://www.ncbi.nlm.nih.gov/pmc/articles/PMC7481268/) | [Pachajoa et al. (2020)](https://www.ncbi.nlm.nih.gov/pmc/articles/PMC7481268/) | | [Mount Sinai](https://www.mountsinai.org/health-library/diseases-conditions/progeria#:~:text=Progeria%20causes%20early%20death.,the%20heart%20or%20a%20stroke.) | | Author's Assumption | | N/A | [Pachajoa et al. (2020)](https://www.ncbi.nlm.nih.gov/pmc/articles/PMC7481268/) | | [FDA (2020)](https://www.accessdata.fda.gov/scripts/cder/daf/index.cfm?event=overview.process&varApplNo=213969) | |
| Endocrinology/Metabolism/Bone | Growth Hormone Deficiency | No | 9 | 30% | N/A | | 78.0 | 69.0 | 0.95 | 2.4 | Yes | [NORD (2016)](https://rarediseases.org/rare-diseases/growth-hormone-deficiency/#affected) | | | [OHSU](https://www.ohsu.edu/brain-institute/growth-hormone-deficiency#:~:text=Incidence%3A%20Growth%20hormone%20deficiency%20is,affects%20about%2050%2C000%20U.S.%20adults.) | N/A | | Aguiar-Oliveira et al. (2010) | | Calculation | | [Maghnie et al. (2023)](https://link.springer.com/article/10.1007/s40618-023-02106-3) | [Kaplowitz et al. (2021)](https://www.jmcp.org/doi/full/10.18553/jmcp.2021.21030) | | [FDA (2023)](https://www.accessdata.fda.gov/scripts/cder/daf/index.cfm?event=overview.process&varApplNo=761184) | |
| Dermatology | Actinic Keratosis | No | 40 | 11% | 23% | | 79.8 | 39.8 | 0.94 | 18750.0 | No | [Mayo Clinic (2022)](https://www.mayoclinic.org/diseases-conditions/actinic-keratosis/symptoms-causes/syc-20354969) | | | [Shaw et al. (2012)](https://www.ncbi.nlm.nih.gov/pmc/articles/PMC3130508/) | [Reinehr and Bakos (2019)](https://www.ncbi.nlm.nih.gov/pmc/articles/PMC6939186/#:~:text=A%20population%20study%20conducted%20in,and%20a%20prevalence%20of%2023%25.) | | [Cleveland Clinic (2020)](https://my.clevelandclinic.org/health/diseases/14148-actinic-keratosis) | |  | | [So et al. (2021)](https://onlinelibrary.wiley.com/doi/epdf/10.1002/ski2.51) | [Reinehr and Bakos (2019)](https://www.ncbi.nlm.nih.gov/pmc/articles/PMC6939186/) | | [FDA (2020)](https://www.accessdata.fda.gov/scripts/cder/daf/index.cfm?event=overview.process&varApplNo=213189) | |
| Neurology | Migraine | No | 25 | 11% | 2% | | 78.5 | 37.6 | 0.94 | 10394.9 | No | [Evans et al. (2014)](https://practicalneurology.com/articles/2014-apr/the-clinical-features-of-migraine-with-and-without-aura) | | | [Lateef et al. (2009)](https://www.ncbi.nlm.nih.gov/pmc/articles/PMC2794247/) | [CDC (2023)](https://www.cdc.gov/mmwr/volumes/72/wr/pdfs/mm7222a6-H.pdf) | | [NVSS (2025)](https://www.cdc.gov/nchs/data/nvsr/nvsr74/nvsr74-02.pdf) | | [Schoenbrunner et al. (2020)](https://www.drjeffreyjanis.com/wp-content/uploads/2020/04/application-pdf.pdf) | | [ICER et al. (2020)](https://icer.org/wp-content/uploads/2020/11/ICER_Acute-Migraine_Final-Evidence-Report_092221.pdf) | [Sico et al. (2022)](https://www.neurology.org/doi/pdf/10.1212/WNL.0000000000200905) | | [FDA (2023)](https://www.accessdata.fda.gov/scripts/cder/daf/index.cfm?event=overview.process&varApplNo=216386) | |
| Hematology/Coagulation | Iron Deficiency Anemia | No | 38 | 13% | 17% | | 79.6 | 41.6 | 0.93 | 66.6 | No | [Lee et al. (2023)](https://jkms.org/DOIx.php?id=10.3346/jkms.2023.38.e299) | | | [Moscheo et al. (2022)](https://www.ncbi.nlm.nih.gov/pmc/articles/PMC9029079/) | [Stauder et al. (2018)](https://pubmed.ncbi.nlm.nih.gov/29141943/) | | [Miller et al. (2013)](https://www.ncbi.nlm.nih.gov/pmc/articles/PMC3685880/) | | Calculation | | [Kim et al. (2019)](https://www.ncbi.nlm.nih.gov/pmc/articles/PMC6567528/) | [Frazao et al. (2005)](https://www.ers.usda.gov/webdocs/publications/42604/30102_aib796-5_002.pdf?v=0) | | [FDA (2019)](https://www.accessdata.fda.gov/scripts/cder/daf/index.cfm?event=overview.process&varApplNo=212320) | |
| Infectious Disease (non viral) | Helicobacter Pylori Infection | No | 58 | 2% | 13% | | 66.2 | 7.7 | 0.90 | 1185.5 | No | [Shah et al. (2024)](https://www.cghjournal.org/action/showPdf?pii=S1542-3565%2823%2900388-9) | | | [Khan et al. (1998)](https://www.annsaudimed.net/doi/10.5144/0256-4947.1998.6?url_ver=Z39.88-2003&rfr_id=ori%3Arid%3Acrossref.org&rfr_dat=cr_pub++0pubmed) | [Khan et al. (1998)](https://www.annsaudimed.net/doi/10.5144/0256-4947.1998.6?url_ver=Z39.88-2003&rfr_id=ori%3Arid%3Acrossref.org&rfr_dat=cr_pub++0pubmed) | | [Jia et al. (2022)](https://bmccancer.biomedcentral.com/articles/10.1186/s12885-022-09222-y) | | Calculation | | [Kowada et al. (2021)](https://www.ncbi.nlm.nih.gov/pmc/articles/PMC9286640/pdf/HEL-26-0.pdf) | [Hooi et al. (2017)](https://www.sciencedirect.com/science/article/pii/S0016508517355312) | | [FDA (2022)](https://www.accessdata.fda.gov/scripts/cder/daf/index.cfm?event=overview.process&ApplNo=215152) | |
| Cardiovascular Disease | Supraventricular Tachycardia | No | 33 | 10% | 85% | | 79.1 | 46.1 | 0.88 | 225.0 | No | [NHS UK](https://www.nhs.uk/conditions/supraventricular-tachycardia-svt/#:~:text=Symptoms%20of%20supraventricular%20tachycardia%20(SVT,25%20and%2040%20years%20old) | | | [Sacks et al (2018)](https://www.researchgate.net/profile/Abhishek-Sharma-39/publication/326679409_PREVALENCE_OF_PAROXYSMAL_SUPRAVENTRICULAR_TACHYCARDIA_PSVT_IN_THE_UNITED_STATES_IN_PATIENTS_UNDER_65_YEARS_OF_AGE/links/5b5e4411458515c4b25222dd/PREVALENCE-OF-PAROXYSMAL-SUPRAVENTRICULAR-TACHYCARDIA-PSVT-IN-THE-UNITED-STATES-IN-PATIENTS-UNDER-65-YEARS-OF-AGE.pdf) | [Sacks et al (2018)](https://www.researchgate.net/profile/Abhishek-Sharma-39/publication/326679409_PREVALENCE_OF_PAROXYSMAL_SUPRAVENTRICULAR_TACHYCARDIA_PSVT_IN_THE_UNITED_STATES_IN_PATIENTS_UNDER_65_YEARS_OF_AGE/links/5b5e4411458515c4b25222dd/PREVALENCE-OF-PAROXYSMAL-SUPRAVENTRICULAR-TACHYCARDIA-PSVT-IN-THE-UNITED-STATES-IN-PATIENTS-UNDER-65-YEARS-OF-AGE.pdf) | | Assumption | | Calculation | | [Farkowski et al (2014)](https://academic.oup.com/europace/article/16/12/1821/489942) | [Sohinki et al (2014)](https://pmc.ncbi.nlm.nih.gov/articles/PMC4295736/#:~:text=EPIDEMIOLOGY%20OF%20SVT&text=The%20incidence%20of%20SVT%20is,1%2C000%20in%20the%20general%20population.&text=Atrial%20fibrillation%20and%20atrial%20flutter,patients%20in%20the%20United%20States.) | | [FDA OPD Listing](https://www.accessdata.fda.gov/scripts/opdlisting/oopd/) | |
| Dermatology | Molluscum Contagiosum | No | 5 | 89% | 0% | | 78.0 | 73.0 | 0.88 | 5000.00 | No | [CDC](https://www.cdc.gov/molluscum-contagiosum/about/index.html) | | | [Reynolds et al (2009)](https://journals.plos.org/plosone/article?id=10.1371/journal.pone.0005255) | [Reynolds et al (2009)](https://journals.plos.org/plosone/article?id=10.1371/journal.pone.0005255) | | Assumption | | Calculation | | [Olsen et al (2016)](https://pubmed.ncbi.nlm.nih.gov/26686685/) | [Hebert et al (2023)](https://pmc.ncbi.nlm.nih.gov/articles/PMC10453394/#:~:text=INCIDENCE%20AND%20PREVALENCE,in%20Table%201.6%E2%80%9311) | | FDA OPD Listing | |
| Renal Disease | Primary Immunoglobulin A nephropathy (IgAN) | Yes, verified | 26 | 8% | 12% | | 65.7 | 40.2 | 0.87 | 32.9 | Yes | [AKF (2024)](https://www.kidneyfund.org/all-about-kidneys/other-kidney-diseases/iga-nephropathy#:~:text=With%20IgAN%2C%20a%20kidney%20biopsy,between%20ages%2016%20and%2035.) | | | [Lerma et al. (2023)](https://pmc.ncbi.nlm.nih.gov/articles/PMC10457441/) | [Lerma et al. (2023)](https://pmc.ncbi.nlm.nih.gov/articles/PMC10457441/) | | [Hastings et al. (2017)](https://pmc.ncbi.nlm.nih.gov/articles/PMC5762959/) | | Calculation | | [Ramjee et al. (2022)](https://www.tandfonline.com/doi/full/10.2147/CEOR.S389456#d1e803) | [Lerma et al. (2023)](https://pmc.ncbi.nlm.nih.gov/articles/PMC10457441/) | | [FDA](https://www.accessdata.fda.gov/scripts/cder/daf/index.cfm?event=BasicSearch.process) | |
| Gastroenterology | Chronic Idiopathic Constipation (CIC) | No | 44 | 2% | 30% | | 80.2 | 36.2 | 0.87 | 14500.0 | No | [McCormick (2019)](https://www.ajmc.com/view/managing-costs-and-care-for-chronic-idiopathic-constipation) | | | [Felt et al. (1999)](https://jamanetwork.com/journals/jamapediatrics/fullarticle/346584#:~:text=Chronic%20constipation%20and%20soiling%20are,often%20precede%20diagnosis%20for%20years.) | [Wald (2024)](https://www.uptodate.com/contents/etiology-and-evaluation-of-chronic-constipation-in-adults#:~:text=The%20prevalence%20of%20chronic%20constipation%20increases%20with%20age%2C%20most%20dramatically,constipation%20%5B10%2C11%5D.) | | [Calculated](https://www.thelancet.com/pdfs/journals/langlo/PIIS2214-109X(17)30073-6.pdf) | | [McCormick (2019)](https://www.ajmc.com/view/managing-costs-and-care-for-chronic-idiopathic-constipation) | | [Pennington et al. (2018)](https://link.springer.com/article/10.1007/s41669-017-0065-9) | [Oh et al. (2020)](https://journals.lww.com/ajg/fulltext/2020/06000/chronic_constipation_in_the_united_states__results.19.aspx#:~:text=Chronic%20idiopathic%20constipation%20(CIC)%20is,medication%20use%20in%20the%20community.) | | [FDA (2021)](https://www.accessdata.fda.gov/scripts/cder/daf/index.cfm?event=overview.process&varApplNo=211281) | |
| Endocrinology/Metabolism/Bone | Noncirrhotic Non-Alcoholic Steatohepatitis (NASH) | Yes | 50 | 14.2% | 30% | | 80.9 | 30.9 | 0.85 | 2209.0 | No | [American Liver Foundation](https://liverfoundation.org/liver-diseases/fatty-liver-disease/nonalcoholic-steatohepatitis-nash/#:~:text=NAFLD%20Tendencies&text=However%2C%20some%20people%20have%20NAFLD,in%20women%20than%20in%20men.) | | | [Yu et al (2021)](https://pmc.ncbi.nlm.nih.gov/articles/PMC8043694/#:~:text=Conclusion,%25%20and%2020%25%2C%20respectively.) | [Hamid et al (2022)](https://www.sciencedirect.com/science/article/pii/S1665268122000692) | | Calculation | |  | | [Fishman et al (2025)](https://hqlo.biomedcentral.com/articles/10.1186/s12955-023-02195-x/tables/2) | [NIDDK](https://www.niddk.nih.gov/health-information/liver-disease/nafld-nash/definition-facts#:~:text=Only%20a%20small%20number%20of,of%20U.S.%20adults%20have%20NASH) | | FDA OPD Listing | |
| Endocrinology/Metabolism/Bone | Type 1 Diabetes | No | 9 | 32% | 8% | | 68.5 | 59.8 | 0.85 | 601.8 | Yes | [Mayo Clinic (2023)](https://www.mayoclinic.org/diseases-conditions/type-1-diabetes/symptoms-causes/syc-20353011#:~:text=Type%201%20diabetes%20can%20appear%20at%20any%20age%2C%20but%20it,10%20and%2014%20years%20old.) | | | [Menke et al. (2015)](https://www.ncbi.nlm.nih.gov/pmc/articles/PMC4562437/) | [Menke et al. (2015)](https://www.ncbi.nlm.nih.gov/pmc/articles/PMC4562437/) | | [Burke (2022)](https://www.healthcentral.com/condition/type-1-diabetes/type-1-diabetes-life-expectancy) | | Calculation | | [Lee et al. (2011)](https://www.ncbi.nlm.nih.gov/pmc/articles/PMC3210197/pdf/nihms282822.pdf) | [CDC (2023)](https://www.cdc.gov/diabetes/data/statistics-report/index.html#:~:text=352%2C000%20children%20and%20adolescents%20younger,1%20diabetes%20and%20using%20insulin.) | | [FDA (2023)](https://www.fda.gov/media/169921/download?attachment) | |
| Endocrinology/Metabolism/Bone | Hypercholesterolemia | No | 47 | 7% | 15% | | 60.0 | 11.5 | 0.85 | 400.0 | No | [Kindt et al. (2016)](https://www.ahajournals.org/doi/epdf/10.1161/CIRCGENETICS.116.001381) | | | [CDC (2023)](https://www.cdc.gov/cholesterol/facts.htm) | [Antza et al. (2024)](https://www.ncbi.nlm.nih.gov/pmc/articles/PMC10813545/) | | [Krogh et al. (2016)](https://watermark.silverchair.com/ehv602.pdf?token=AQECAHi208BE49Ooan9kkhW_Ercy7Dm3ZL_9Cf3qfKAc485ysgAAA18wggNbBgkqhkiG9w0BBwagggNMMIIDSAIBADCCA0EGCSqGSIb3DQEHATAeBglghkgBZQMEAS4wEQQMZGBSMLa8nF9ciGFDAgEQgIIDEuTa1aAhSfb_0xHG2eJWKe09hV4d53DHA42L168ZQ3aQ8-lZs3Rwcd6MDBasg2TXRvwIPHCZoNE_aW4g7UN5su0GezFtKJKmZqMgvlGDjHDUHuWzfbFGVDlg2P8kUstRssm2URsnD-3qldTH0wcN9s_jpwTzVi2vLzr1bgDkK_iNLyuBAMsYKyEv-H2zO_lzchRZaFaS_6atL29iCSmHJ_o5OnO5SadIoT7ehHJgU5hVZ8PtDtfA1M0yrqdPN8u6y6Rm34-wxJlOogPZ0WYNtPGNUc_eaX4wuwoSNUe2x4wYBKBxS9qOXMTUisV3fHp0tjh2K7uJ33QhNOrSvUioH5UH1PHef-2dwzwuyks2BwzEG9Y2g39hbjUUNG8i4EVCsQ-jRrYBQd-FKvMgbVG36I-jML0Kob-EXmVJEocTxZHhphS2EtjFRszUz6Hp8QuEroy4JzKS5JRXOAfDlsO-FJ6a3oLdR73ZZIxwG332fL9jrrLqheHjAj1_yU9XaXixBX2A_OSmZsZTzv2FFlDLngASRRcOX45xx550t2zPcPkmX4nzGXLFmQBJ_wvRegQMR1XmH5NkPIeYe9-4Gri2CeyjMQuxe-seEPpevwR3BpWgWbbjgH81LHxcwF2K2aKeipqkUVtyhgkvm5QoMcIe757KCutY9FzcJmvlKaC4dMfko2C-bSkDqbml49PYXdbdjmFcT4i6kGESl2IWGlqYQRgxG8W0ntGW5bAMsZvx-DOQoeEJ1Kwfjzz_YCManELEkqsFpE5kIhNXJ-M7ZakK35tIVA6Uq5bm47ukPqmHvT_GTnUFHSpCPH7rqsr-9lGjXCVAyYTo8tgJlzscR2stEFzcoZegJ1oYxqSR1qQ0S08GGmCLbLJw46ZuI4fLXetJiSoyAUrY5V7-N--xX7RhRMK3bgM7zow3fzckEJjTavnbGBXdjBLhNAJs9JeqfFeVi_dferrLiJ4WIIXoWEj2D9rBs4MLkd9Io2-VyoG8lHnJKmLnzRN8Wt05y8ZgpvLno-8oAsKC1AxyvsevMhAvnVVrCw) | | [ICER et al. (2021)](https://icer.org/wp-content/uploads/2022/04/High-Cholesterol_Final-Report_12-Month-Update_041222.pdf) | | [ICER et al. (2021)](https://icer.org/wp-content/uploads/2022/04/High-Cholesterol_Final-Report_12-Month-Update_041222.pdf) | [Akioyamen et al. (2017)](https://www.ncbi.nlm.nih.gov/pmc/articles/PMC5588988/pdf/bmjopen-2017-016461.pdf) | | [FDA (2021)](https://www.accessdata.fda.gov/scripts/cder/daf/index.cfm?event=overview.process&varApplNo=214012) | |
| Infectious Disease (non viral) | Complicated Urinary Tract Infections (cUTI) | No | 48 | 1% | 70% | | 80.7 | 32.7 | 0.85 | 900.9 | No | [Carreno et al. (2019)](https://academic.oup.com/ofid/article/6/11/ofz446/5601270) | | | [Zilberberg et al. (2022)](https://www.ncbi.nlm.nih.gov/pmc/articles/PMC8754377/) | [Zilberberg et al. (2022)](https://www.ncbi.nlm.nih.gov/pmc/articles/PMC8754377/) | | [Kauf et al. (2017)](https://www.ncbi.nlm.nih.gov/pmc/articles/PMC5410095/) | | Calculation | | [Kauf et al. (2017)](https://bmcinfectdis.biomedcentral.com/articles/10.1186/s12879-017-2408-7) | [Lodise et al. (2022)](https://www.ncbi.nlm.nih.gov/pmc/articles/PMC9310258/) | | [FDA (2021)](https://www.accessdata.fda.gov/scripts/cder/daf/index.cfm?event=overview.process&varApplNo=209445) | |
| Infectious Disease (non viral) | Complicated Intra-abdominal Infections (cIAI) | No | 52 | 0% | 18% | | 62.0 | 14.9 | 0.85 | 35.0 | No | [Sartelli et al. (2014)](https://www.ncbi.nlm.nih.gov/pmc/articles/PMC4039043/) | | | [Chen et al. (2020)](https://www.sciencedirect.com/science/article/pii/S168411821830286X) | [Podnos et al. (2002)](https://academic.oup.com/cid/article/35/1/62/282276) | | [Pan et al. (2019)](https://www.dovepress.com/getfile.php?fileID=72934) | | [Prabhu et al. (2017)](https://aricjournal.biomedcentral.com/articles/10.1186/s13756-017-0264-2#:~:text=Results,12.70%2Fpatient%2C%20respectively).) | | [Prabhu et al. (2017)](https://aricjournal.biomedcentral.com/articles/10.1186/s13756-017-0264-2#:~:text=Results,12.70%2Fpatient%2C%20respectively).) | [Bhisey (2022)](https://www.biospace.com/article/intra-abdominal-infections-market-growing-incidence-of-infectious-and-chronic-diseases-to-drive-the-market/#:~:text=A%20wide%20range%20of%20antibiotics,treatment%20of%20intra%2Dabdominal%20infection.&text=According%20to%20recent%20studies%20conducted,3.5%20million%20cases%20per%20year.) | | [FDA (2019)](https://www.accessdata.fda.gov/scripts/cder/daf/index.cfm?event=overview.process&varApplNo=212819) | |
| Neurology | Narcolepsy | No | 16 | 12% | 0% | | 78.1 | 62.1 | 0.85 | 37.7 | Yes | [Morse (2019)](https://www.mdpi.com/2076-3271/7/12/106) | | | [Scheer et al. (2019)](https://academic.oup.com/sleep/article/42/7/zsz091/5475508?login=false#137509551) | [Billiard (2010)](https://www.cambridge.org/core/books/abs/principles-and-practice-of-geriatric-sleep-medicine/narcolepsy-in-the-elderly/E0BCD3455E29AB2163EE67267A6A7F2F) | | [Schwab (2022)](https://www.merckmanuals.com/home/brain,-spinal-cord,-and-nerve-disorders/sleep-disorders/narcolepsy) | | Calculated | | [Tadrous et al. (2021)](https://onlinelibrary.wiley.com/doi/full/10.1111/jsr.13383) | [Ohayon et al. (2023)](https://www.sciencedirect.com/science/article/pii/S2590142723000356) | | [FDA (2022)](https://www.accessdata.fda.gov/scripts/cder/daf/index.cfm?event=BasicSearch.process) | |
| Pulmonary | Chronic Obstructive Pulmonary Disease (COPD) | No | 65 | 0% | 55% | | 71.9 | 6.79 | 0.83 | 5057.0 | No | [Wacker et al (2016)](https://bmcpulmmed.biomedcentral.com/articles/10.1186/s12890-016-0238-9) | | | [Roberts et al (2024)](https://pmc.ncbi.nlm.nih.gov/articles/PMC11100519/) | [Wacker et al (2016)](https://bmcpulmmed.biomedcentral.com/articles/10.1186/s12890-016-0238-9/tables/1) | | [Chen et al (2020)](https://www.resmedjournal.com/article/S0954-6111(20)30272-9/fulltext) | | Calculation | | [Wacker et al (2016)](https://bmcpulmmed.biomedcentral.com/articles/10.1186/s12890-016-0238-9) | [CDC](https://www.cdc.gov/mmwr/volumes/72/wr/mm7246a1.htm) | | FDA OPD Listing | |
| Cardiovascular Disease | Obstructive Hypertrophic Cardiomyopathy | No | 39 | 0% | 48% | | 79.7 | 40.7 | 0.83 | 200.0 | Yes | [HCMA (2021)](https://4hcm.org/hcm-development/) | | | [Miyake (2020)](https://emedicine.medscape.com/article/890068-overview#a5) | [Butzner et al. (2022)](https://www.sciencedirect.com/science/article/pii/S0002914922003381) | | [AHA (2022)](https://www.heart.org/en/health-topics/cardiomyopathy/what-is-cardiomyopathy-in-adults/hypertrophic-cardiomyopathy) | | Calculation | | [Wasfy et al. (2021)](https://icer.org/wp-content/uploads/2021/04/ICER_HCM_Final_Evidence_Report_111621.pdf) | [Butzner et al. (2021)](https://www.ncbi.nlm.nih.gov/pmc/articles/PMC8770922/#:~:text=Hypertrophic%20cardiomyopathy%20is%20the%20most,prevalence%20of%201%20in%20500.) | | [FDA (2023)](https://www.accessdata.fda.gov/scripts/cder/daf/index.cfm?event=overview.process&varApplNo=214998) | |
| Gastroenterology | Irritable Bowel Syndrome with Constipation (IBS-C) | No | 19 | 2% | 15% | | 78.2 | 59.2 | 0.83 | 2049.6 | No | [Phillips (2023)](https://www.pennmedicine.org/for-patients-and-visitors/patient-information/conditions-treated-a-to-z/irritable-bowel-syndrome) | | | [Hyams et al. (2016)](https://www.gastrojournal.org/article/S0016-5085(16)00181-5/fulltext?referrer=https%3A%2F%2Fpubmed.ncbi.nlm.nih.gov%2F) | [Smith-Garcia (2022)](https://www.healthline.com/health/irritable-bowel-syndrome/irritable-bowel-syndrome-in-older-adults#:~:text=However%2C%20the%20rate%20of%20diagnosis,consistent%20with%20an%20IBS%20diagnosis.) | | [Staller et al. (2021)](https://www.ncbi.nlm.nih.gov/pmc/articles/PMC7196022/) | | Calculation | | [Huang et al. (2014)](https://www.tandfonline.com/doi/pdf/10.3111/13696998.2014.979291) | [Almario et al. (2023)](https://www.gastrojournal.org/action/showPdf?pii=S0016-5085%2823%2904889-8) | | [FDA (2022)](https://www.accessdata.fda.gov/scripts/cder/daf/index.cfm?event=overview.process&varApplNo=211801) | |
| Ophthalmology | Demodex Blepharitis | No | 60 | 12% | 65% | | 82.7 | 22.7 | 0.82 | 7507.5 | No | [Flores et al. (2023)](https://www.sciencedirect.com/science/article/pii/S032575412300055X) | | | [Zhang et al. (2021)](https://journals.lww.com/claojournal/abstract/2021/01000/prevalence_of_ocular_demodex_infestation_in.11.aspx) | [Flores et al. (2023)](https://www.sciencedirect.com/science/article/pii/S032575412300055X) | | Author's Assumption | | Calculation | | [Rhee et al. (2023)](https://www.ncbi.nlm.nih.gov/pmc/articles/PMC10351901/#:~:text=Dry%20Eye%20Disease%20(DED)&text=There%20is%20significant%20overlap%20in,not%20correlate%20with%20Demodex%20blepharitis.) | [Stanley (2022)](https://www.hmpgloballearningnetwork.com/site/frmc/literature-review/look-impact-prevalence-demodex-blephartis) | | [FDA (2023)](https://www.accessdata.fda.gov/scripts/cder/daf/index.cfm?event=overview.process&varApplNo=217603) | |
| Ophthalmology | Dry Eye Disease | No | 57 | 6% | 15% | | 82.1 | 25.1 | 0.82 | 164.0 | No | [Miller et al. (2021)](https://iovs.arvojournals.org/article.aspx?articleid=2774775) | | | [Stapleton et al. (2024)](https://www.sciencedirect.com/science/article/pii/S1542012423001520#:~:text=A%20literature%20search%2C%20conducted%20from,from%205.5%25%20to%2023.1%20%25.) | [Farrand et al. (2017)](https://www.sciencedirect.com/science/article/pii/S0002939417302908#:~:text=Participants%20on%20government%20insurance%20reported,%25)%20in%20the%20age%20%3C65) | | [NVSS (2025)](https://www.cdc.gov/nchs/data/nvsr/nvsr74/nvsr74-02.pdf) | | Calculation | | [Shigeyasu et al. (2018)](https://www.ncbi.nlm.nih.gov/pmc/articles/PMC6119313/pdf/12955_2018_Article_999.pdf) | [Farrand et al. (2017)](https://www.sciencedirect.com/science/article/pii/S0002939417302908#:~:text=Participants%20on%20government%20insurance%20reported,%25)%20in%20the%20age%20%3C65) | | [FDA (2023)](https://www.accessdata.fda.gov/scripts/cder/daf/index.cfm?event=overview.process&varApplNo=216675) | |
| Urologic | Overactive Bladder (OAB) | No | 52 | 18% | 19% | | 81.2 | 11.6 | 0.82 | 775.9 | No | [Pray et al. (2013)](https://www.uspharmacist.com/article/a-new-nonprescription-product-for-overactive-bladder-in-women-42378) | | | [Ramsay et al. (2017)](https://www.ncbi.nlm.nih.gov/pmc/articles/PMC5332242/) | [Wallace et al. (2015)](https://www.ncbi.nlm.nih.gov/pmc/articles/PMC4754030/#:~:text=The%20prevalence%20of%20overactive%20bladder,urgency%20having%20the%20biggest%20impact.) | | Author's Assumption | | [Murray et al. (2023)](https://becarispublishing.com/doi/10.2217/cer-2022-0089) | | [Desroziers et al. (2013)](https://www.ncbi.nlm.nih.gov/pmc/articles/PMC3842710/) | [Reynolds et al. (2016)](https://www.ncbi.nlm.nih.gov/pmc/articles/PMC4821440/) | | [FDA (2020)](https://www.accessdata.fda.gov/scripts/cder/daf/index.cfm?event=overview.process&varApplNo=213006) | |
| Hematology/Coagulation | Uncontrolled Bleeding | Yes | 65 | 0% | 55% | | 83.2 | 18.5 | 0.82 | 337.8 | No | [Johnston et al. (2023)](https://www.tandfonline.com/doi/full/10.2147/CEOR.S411778#d1e229) | | | [Corral et al. (2015)](https://www.tandfonline.com/doi/full/10.2147/CEOR.S86369?role=tab&tab=permissions&scroll=top#d1e312) | [Afolabi et al. (2023)](https://pmc.ncbi.nlm.nih.gov/articles/PMC10706582/) | | Calculation | | [Johnston et al. (2023)](https://www.tandfonline.com/doi/full/10.2147/CEOR.S411778#d1e229) | | [Res et al. (2019)](https://www.sciencedirect.com/science/article/pii/S1109966618301313#sec3) | [Stokes et al. (2011)](https://pmc.ncbi.nlm.nih.gov/articles/PMC3126703/#:~:text=Results,and%20reproductive%20organ%20($2%2C805) | | [FDA](https://www.accessdata.fda.gov/scripts/cder/daf/index.cfm?event=BasicSearch.process) | |
| Endocrinology/Metabolism/Bone | Classic Congenital Adrenal Hyperplasia (CAH) | No | 0 | 22% | 18% | | 41.20 | 41.2 | 0.82 | 10.0 | Yes |  | | |  |  | | [Falhammar et al (2014)](https://pubmed.ncbi.nlm.nih.gov/25279502/) | | Calculation | | [Zainuddin et al (2020)](https://hqlo.biomedcentral.com/articles/10.1186/s12955-020-01515-9?utm_source=chatgpt.com) | [Cleveland Clinic](https://my.clevelandclinic.org/health/diseases/17817-congenital-adrenal-hyperplasia#overview) | | FDA OPD Listing | |
| Hematology/Coagulation | Paroxysmal Nocturnal Hemoglobinuria (PNH) | No | 36 | 4% | 12% | | 67.3 | 31.8 | 0.81 | 1.3 | Yes | [Clayton et al. (2024)](https://journals.sagepub.com/doi/10.1177/10760296231213073) | | | [Urbano-Ispizua et al. (2017)](https://www.ncbi.nlm.nih.gov/pmc/articles/PMC5394949/) | [Clayton et al. (2024)](https://journals.sagepub.com/doi/10.1177/10760296231213073) | | [Sørensen, Hansen, and Frederiksen (2023)](https://www.ncbi.nlm.nih.gov/pmc/articles/PMC10653694/) | | Calculation | | [O'Connell et al. (2020)](https://link.springer.com/article/10.1007/s40273-020-00929-z) | [Jalbert et al. (2019)](https://ashpublications.org/blood/article/134/Supplement_1/3407/428186/Epidemiology-of-PNH-and-Real-World-Treatment) | | [FDA (2023)](https://www.accessdata.fda.gov/scripts/cder/daf/index.cfm?event=overview.process&varApplNo=215014) | |
| Dermatology | Primary axillary hyperhidrosis | No | 17 | 77% | 2% | | 78.1 | 61.1 | 0.80 | 1400.0 | No | [Glaser et al. (2016)](https://www.sweathelp.org/pdf/IHHS%20-%20Prevalence.pdf#:~:text=According%20to%20the%20research%20published%20in%202004%2C,patients%20than%20previously%20thought%20(see%20Figure%203).) | | | [Glaser et al. (2016)](https://www.sweathelp.org/pdf/IHHS%20-%20Prevalence.pdf#:~:text=According%20to%20the%20research%20published%20in%202004%2C,patients%20than%20previously%20thought%20(see%20Figure%203).) | [Glaser et al. (2016)](https://www.sweathelp.org/pdf/IHHS%20-%20Prevalence.pdf#:~:text=According%20to%20the%20research%20published%20in%202004%2C,patients%20than%20previously%20thought%20(see%20Figure%203).) | | [NVSS (2025)](https://www.cdc.gov/nchs/data/nvsr/nvsr74/nvsr74-02.pdf) | | Calculation | | [Bloudek et al. (2021)](https://www.tandfonline.com/doi/full/10.1080/13696998.2020.1855879?rfr_dat=cr_pub++0pubmed&url_ver=Z39.88-2003&rfr_id=ori%3Arid%3Acrossref.org#d1e309) | [Oshima et al. (2023)](https://onlinelibrary.wiley.com/doi/10.1111/1346-8138.16908) | | FDA | |
| Cardiovascular Disease | Transthyretin Mediated Amyloidosis - Cardiomyopathy (ATTR-CM) | No | 74 | 0% | 100% | | 86.6 | 3.5 | 0.80 | 0.2 | Yes | [Ruberg et al (2019)](https://www.sciencedirect.com/science/article/pii/S0735109719347291) | | | [Ruberg et al (2019)](https://www.sciencedirect.com/science/article/pii/S0735109719347291) | [Ruberg et al (2019)](https://www.sciencedirect.com/science/article/pii/S0735109719347291) | |  | | Ruberg et al (2019) | | [Eldhangen et al (2023)](https://pmc.ncbi.nlm.nih.gov/articles/PMC10192258/) | [Delgado et al (2025)](https://ojrd.biomedcentral.com/articles/10.1186/s13023-025-03547-0#:~:text=In%20the%20US%2C%20the%20prevalence,higher%20%5B26%2C%2027%5D.) | | [FDA OPD Listing](https://www.accessdata.fda.gov/scripts/opdlisting/oopd/) | |
| Endocrinology/Metabolism/Bone | Long-chain Fatty Acid Oxidation Disorders (LC-FAOD) | No | 4.5 | 61% | 0% | | 23.0 | 18.5 | 0.80 | 0.9 | Yes | [Ambrose et al. (2022)](https://ojrd.biomedcentral.com/articles/10.1186/s13023-022-02512-5#:~:text=Six%20patients%20identified%20by%20positive,1%20month%2D8%20years).) | | | [Kruger et al. (2022)](https://www.sciencedirect.com/science/article/pii/S2214426922000635) | N/A | | Calculation | | [Ultragenyx Canada Inc. (2022)](https://canjhealthtechnol.ca/index.php/cjht/article/view/sr0684r/623) | | [Kruger et al. (2022)](https://www.sciencedirect.com/science/article/pii/S2214426922000635) | [Kruger, McNiven, and Marsden (2022)](https://www.ncbi.nlm.nih.gov/pmc/articles/PMC9239941/) | | [FDA (2023)](https://www.accessdata.fda.gov/scripts/cder/daf/index.cfm?event=overview.process&varApplNo=213687) | |
| Immunomodulators | WHIM Syndrome | No | 2 | 100% | 0% | | 55.0 | 52.8 | 0.79 | 0.025 | Yes | [Walter et al (2019)](https://www.jaci-inpractice.org/article/S2213-2198(19)30250-8/fulltext#:~:text=With%20the%20clinical%20onset%20at,a%20patient%20presenting%20with%20neutropenia.) | | | [Walter et al (2019)](https://www.jaci-inpractice.org/article/S2213-2198(19)30250-8/fulltext#:~:text=With%20the%20clinical%20onset%20at,a%20patient%20presenting%20with%20neutropenia.) | [Walter et al (2019)](https://www.jaci-inpractice.org/article/S2213-2198(19)30250-8/fulltext#:~:text=With%20the%20clinical%20onset%20at,a%20patient%20presenting%20with%20neutropenia.) | | [Badolato et al (2024)](https://ashpublications.org/blood/article/144/1/35/515799/A-phase-3-randomized-trial-of-mavorixafor-a-CXCR4) | | Calculation | | [Badolato et al (2024)](https://ashpublications.org/blood/article/144/1/35/515799/A-phase-3-randomized-trial-of-mavorixafor-a-CXCR4) | [Geier et al (2022)](https://pmc.ncbi.nlm.nih.gov/articles/PMC9700649/) | | [FDA OPD Listing](https://www.accessdata.fda.gov/scripts/opdlisting/oopd/) | |
| Ophthalmology | Diabetic Macular Edema | No | 22 | 0% | 3% | | 78.4 | 56.4 | 0.79 | 224.0 | No | [Romero-Aroca (2011)](https://www.ncbi.nlm.nih.gov/pmc/articles/PMC3158878/) | | | [Chang et al. (2021)](https://iovs.arvojournals.org/article.aspx?articleid=2773563#:~:text=The%20prevalence%20in%20children%20with,were%20rarely%20observed%20in%20children.) | [Lundeen et al. (2022)](https://pubmed.ncbi.nlm.nih.gov/35238912/) | | Author's Assumption | | Calculation | | [Vujosevic et al. (2024)](https://www.ncbi.nlm.nih.gov/pmc/articles/PMC10585645/) | [Varma et al. (2014)](https://pubmed.ncbi.nlm.nih.gov/25125075/) | | [FDA (2023)](https://www.accessdata.fda.gov/scripts/cder/daf/index.cfm?event=overview.process&varApplNo=761235) | |
| Dermatology | Alopecia Areata (AA) | No | 34 | 16% | 25% | | 79.2 | 45.2 | 0.79 | 210.0 | No | [Mirzoyev et al (2014)](https://www.jidonline.org/article/S0022-202X(15)36736-1/fulltext) | | | [Sy et al (2023)](https://jamanetwork.com/journals/jamadermatology/fullarticle/2801704) | [Sy et al (2023)](https://jamanetwork.com/journals/jamadermatology/fullarticle/2801704) | |  | |  | | [Liu et al (2018)](https://www.jaad.org/article/S0190-9622(18)30183-X/fulltext) | [Benigno et al (2020)](https://pmc.ncbi.nlm.nih.gov/articles/PMC7131990/#:~:text=self%2Dreported%20AA.-,Conclusion,approved%20treatments%20will%20be%20critical.) | | [FDA OPD Listing](https://www.accessdata.fda.gov/scripts/opdlisting/oopd/) | |
| Dermatology | Thermal Burns | No | N/A | 11% | 16% | | 77.5 | N/A | 0.79 | 145.9 | Yes | N/A | | | [Atwell et al. (2021)](https://www.ncbi.nlm.nih.gov/pmc/articles/PMC7036333/) | [Goei et al. (2020)](https://academic.oup.com/burnstrauma/article/doi/10.1093/burnst/tkaa027/5934908) | | [Maani (2023)](https://www.ncbi.nlm.nih.gov/books/NBK539773/) | | N/A | | [Rashaan et al. (2020)](https://onlinelibrary.wiley.com/doi/full/10.1111/wrr.12799) | [ABA (2016)](https://ameriburn.org/who-we-are/media/burn-incidence-fact-sheet/) | | [FDA (2022)](https://www.accessdata.fda.gov/scripts/cder/daf/index.cfm?event=overview.process&varApplNo=761192) | |
| Neurology | Acute Hepatic Porphyria (AHP) | No | 24 | N/A | N/A | | 53.0 | 11.5 | 0.78 | 1.0 | Yes | [Dickey et al. (2022)](https://www.ncbi.nlm.nih.gov/pmc/articles/PMC9830021/) | | | N/A | N/A | | [Baumann and Kauppinen (2022)](https://www.sciencedirect.com/science/article/pii/S2214426922000027) | | [NICE (2021)](https://www.nice.org.uk/guidance/hst16/documents/1-2) | | [Gouya et al. (2020)](https://www.ncbi.nlm.nih.gov/pmc/articles/PMC7255459/) | [Porphyria Diagnosis](https://www.porphyria.com/hcp/about-ahp#:~:text=Epidemiology%20of%20AHP,ages%20of%2015%20and%2045.) | | [FDA (2023)](https://www.accessdata.fda.gov/scripts/cder/daf/index.cfm?event=overview.process&varApplNo=212194) | |
| Psychiatry | Attention Deficit Hyperactivity Disorder (ADHD) -- children/adolescents | No | 7 | 100% | 0% | | 68.4 | 61.4 | 0.78 | 1791.3 | No | [APA (2013)](https://www.psychiatry.org/File%20Library/Psychiatrists/Practice/DSM/APA_DSM-5-ADHD.pdf) | | | Author's Assumption | Author's Assumption | | [Barkley and Fischer (2018)](https://journals.sagepub.com/doi/10.1177/1087054718816164?url_ver=Z39.88-2003&rfr_id=ori:rid:crossref.org&rfr_dat=cr_pub%20%200pubmed) | | Calculation | | [Thai et al. (2023)](https://link.springer.com/article/10.1007/s11136-023-03441-x) | [CDC (2023)](https://www.cdc.gov/ncbddd/adhd/data.html) | | [FDA (2023)](https://www.accessdata.fda.gov/scripts/cder/daf/index.cfm?event=BasicSearch.process) | |
| Allergy | Hereditary Angioedema (HAE) | No | 13 | 50% | 7% | | 71.9 | 59.4 | 0.77 | 2.0 | Yes | [Banerji et al. (2020)](https://www.sciencedirect.com/science/article/pii/S1081120620301459) | | | [Farkas (2010)](https://aacijournal.biomedcentral.com/articles/10.1186/1710-1492-6-18) | [Banerji et al. (2020)](https://www.sciencedirect.com/science/article/pii/S1081120620301459) | | [Perego et al. (2020)](https://www.jaci-inpractice.org/article/S2213-2198(20)30046-5/fulltext) | | Calculation | | [Bloudek et al. (2021)](https://icer.org/wp-content/uploads/2020/10/ICER_Aetion_RWE_HAE_Report_082421.pdf) | [Ghazi and Grant (2013)](https://www.ncbi.nlm.nih.gov/pmc/articles/PMC3647445/) | | [FDA (2023)](https://www.accessdata.fda.gov/scripts/cder/daf/index.cfm?event=overview.process&varApplNo=214094) | |
| Neurology | Fabry Disease | Yes | 11 | 17% | 13% | | 66.6 | 40.5 | 0.77 | 2.5 | No | [Brady et al. (2015)](https://academic.oup.com/qjmed/article/108/7/585/1567321) | | | [Beck et al. (2006)](https://www.ncbi.nlm.nih.gov/books/NBK11581/) | [Morand et al. (2019)](https://link.springer.com/article/10.1007/s12325-019-01061-x/tables/1) | | [Bionews](https://fabrydiseasenews.com/fabry-disease-life-expectancy/#:~:text=Published%20data%20from%20the%20Fabry,women%20in%20the%20U.S.%20general) | | [Rombach et al. (2013)](https://www.ncbi.nlm.nih.gov/pmc/articles/PMC3598841/) | | [Rombach et al. (2013)](https://www.ncbi.nlm.nih.gov/pmc/articles/PMC3598841/) | [Ahsan (2023)](https://emedicine.medscape.com/article/1952086-overview#a3) | | [FDA (2023)](https://www.accessdata.fda.gov/scripts/cder/daf/index.cfm?event=overview.process&varApplNo=761161) | |
| Cardiovascular Disease | Hypertension | No | 46 | 2% | 35% | | 72.5 | 26.5 | 0.77 | 35321.2 | No | [Huang et al (2022)](https://jamanetwork.com/journals/jamacardiology/fullarticle/2795050) | | | [Hardy et al (2021)](https://jamanetwork.com/journals/jamanetworkopen/fullarticle/2778082) | [Leszczak et al (2024)](https://pmc.ncbi.nlm.nih.gov/articles/PMC10792009/#:~:text=US%20National%20Health%20and%20Nutrition,and%2034%25%20men%20globally1.) | | [Franco et al (2005)](https://www.ahajournals.org/doi/10.1161/01.hyp.0000173433.67426.9b?) | | Calculation | | [Alzarea et al (2024)](https://pmc.ncbi.nlm.nih.gov/articles/PMC11162240/table/t0002/) | [CDC](https://www.cdc.gov/nchs/products/databriefs/db364.htm) | | FDA OPD Listing | |
| Pulmonary | Cystic Fibrosis | No | 11 | 42% | 0% | | 37.0 | 20.7 | 0.77 | 0.4 | Yes | [CFF; Padoan et al. (2021)](https://www.ncbi.nlm.nih.gov/pmc/articles/PMC7920411/pdf/diagnostics-11-00321.pdf) | | | [CFF (2021)](https://www.cff.org/sites/default/files/2021-11/Patient-Registry-Annual-Data-Report.pdf) | Assumption given median survival is below 65 | | [Singh et al. (2023)](https://www.ncbi.nlm.nih.gov/pmc/articles/PMC10497589/pdf/41598_2023_Article_41868.pdf) | | [ICER et al. (2020)](https://icer.org/wp-content/uploads/2020/08/ICER_CF_Evidence_Report_042720.pdf) | | [ICER et al. (2020)](https://icer.org/wp-content/uploads/2020/08/ICER_CF_Evidence_Report_042720.pdf) | [CFF](https://www.cff.org/intro-cf/about-cystic-fibrosis) | | [FDA (2019)](https://www.accessdata.fda.gov/scripts/cder/daf/index.cfm?event=overview.process&ApplNo=212273) | |
| Neurology | Insomnia | No | 22 | 20% | 17% | | 78.4 | 56.4 | 0.77 | 30000.0 | No | [Ricketts et al. (2023)](https://academic.oup.com/sleep/article/46/Supplement_1/A169/7181669) | | | [Owens (2024)](https://www.uptodate.com/contents/behavioral-sleep-problems-in-children#:~:text=By%20contrast%2C%20according%20to%20standards,term%20the%20insomnia%20as%20chronic.&text=Prevalence%20%E2%80%94%20Chronic%20insomnia%20disorder%20is,exact%20definition%20used%20and%20age.) | [Martin (2022)](https://www.usatoday.com/story/news/health/2022/06/23/survey-insomnia-among-us-adults/7687949001/) | |  | | Calculation | | [Chalet et al. (2023)](https://www.mdpi.com/2077-0383/12/6/2438) | [Roth (2019)](https://jcsm.aasm.org/doi/10.5664/jcsm.26929#:~:text=Chronic%20insomnia%20is%20highly%20prevalent,%2C%20social%2C%20and%20physical%20domains.) | | [FDA (2023)](https://www.accessdata.fda.gov/scripts/cder/daf/index.cfm?event=overview.process&varApplNo=214985) | |
| Endocrinology/Metabolism/Bone | Familial Chylomicronemia Syndrome (FCS) | No | 15 | 90% | 0% | | 31.5 | 16.5 | 0.76 | 1.3 | Yes | [Spagnuolo et al (2024)](https://academic.oup.com/jcem/article/110/4/1158/7750066) | | | [Davidson et al (2018)](https://www.sciencedirect.com/science/article/pii/S1933287418302071) | [Davidson et al (2018)](https://www.sciencedirect.com/science/article/pii/S1933287418302071) | | Calculation | | [Lin et al (2014)](https://www.valueinhealthjournal.com/article/S1098-3015(14)02838-1/fulltext) | | [Williams et al (2023)](https://pmc.ncbi.nlm.nih.gov/articles/PMC10565991/) | [Pallazola et al (2019)](https://www.ahajournals.org/doi/10.1161/circ.140.suppl_1.14986) | | [FDA OPD Listing](https://www.accessdata.fda.gov/scripts/opdlisting/oopd/) | |
| Psychiatry | Hypoactive Sexual Desire Disorder (HSDD) | No | 25 | N/A | 25% | | 78.5 | 53.5 | 0.76 | 5050.0 | No | [Travers (2021)](https://www.verywellhealth.com/hypoactive-sexual-desire-disorder-symptoms-causes-diagnosis-treatment-and-coping-4767525) | | | [Parish and Hahn (2016)](https://pubmed.ncbi.nlm.nih.gov/27872021/) | [Biddle et al. (2009)](https://pubmed.ncbi.nlm.nih.gov/19192259/) | | Assumption | | Calculation | | [Biddle et al. (2009)](https://pubmed.ncbi.nlm.nih.gov/19192259/) | [Kingsberg et al. (2018)](https://www.valueinhealthjournal.com/article/S1098-3015(18)31513-4/fulltext) | | [FDA (2020)](https://www.accessdata.fda.gov/scripts/cder/daf/index.cfm?event=overview.process&varApplNo=210557) | |
| Hematology/Coagulation | Light Chain Amyloidosis | Yes | 63 | 0% | 43% | | 69.3 | 6.5 | 0.75 | 6.9 | Yes | [Cleveland Clinic (2022)](https://my.clevelandclinic.org/health/diseases/15718-amyloidosis-al-amyloid-light-chain) | | | Assumption | [Quock et al. (2018)](https://pmc.ncbi.nlm.nih.gov/articles/PMC5965052/) | | [My Amyloidosis Team](https://www.myamyloidosisteam.com/resources/life-expectancy-with-amyloidosis) | | [My Amyloidosis Team](https://www.myamyloidosisteam.com/resources/life-expectancy-with-amyloidosis) | | [McCausland et al. (2017)](https://www.sciencedirect.com/science/article/pii/S0006497119839368#:~:text=Utility%20scores%20have%20been%20derived%20separately%20for,health%20states%2C%20but%20the%20two%20measures%20often) | [Laires et al. (2023)](https://www.sciencedirect.com/science/article/abs/pii/S0006497123133635) | | [FDA](https://www.accessdata.fda.gov/scripts/cder/daf/index.cfm?event=BasicSearch.process) | |
| Neurology | Partial Onset Seizures (Epilepsy) | No | 42 | 14% | 2% | | 66.4 | 24.7 | 0.75 | 34.4 | No | [Kaur et al. (2018)](https://www.ncbi.nlm.nih.gov/pmc/articles/PMC5958567/pdf/JFMPC-7-191.pdf) | | | [Zack et al. (2017)](https://www.cdc.gov/mmwr/volumes/66/wr/mm6631a1.htm) | [Acharya et al. (2014)](https://www.ncbi.nlm.nih.gov/pmc/articles/PMC4001216/#:~:text=The%20prevalence%20rate%20of%20epilepsy,or%20older%20is%20approximately%201.5%25.) | | [Trinka et al. (2023)](https://www.frontiersin.org/articles/10.3389/fepid.2023.1081757/full) | | Calculation | | [Helmers et al. (2012)](https://www.ejpn-journal.com/article/S1090-3798(12)00002-5/fulltext) | [Zack et al. (2017)](https://www.cdc.gov/mmwr/volumes/66/wr/mm6631a1.htm) | | [FDA (2019)](https://www.accessdata.fda.gov/scripts/cder/daf/index.cfm?event=overview.process&varApplNo=212839) | |
| Psychiatry | Attention Deficit Hyperactivity Disorder (ADHD) -- adults | No | 7 | 0% | 4% | | 63.7 | 56.7 | 0.74 | 2617.7 | No | [APA (2013)](https://www.psychiatry.org/File%20Library/Psychiatrists/Practice/DSM/APA_DSM-5-ADHD.pdf) | | | Author's Assumption | [Vos and Hartman (2022)](https://www.ncbi.nlm.nih.gov/pmc/articles/PMC9197091/) | | [Barkley and Fischer (2018)](https://journals.sagepub.com/doi/10.1177/1087054718816164?url_ver=Z39.88-2003&rfr_id=ori:rid:crossref.org&rfr_dat=cr_pub%20%200pubmed) | | Calculation | | [Joseph et al. (2019)](https://www.ncbi.nlm.nih.gov/pmc/articles/PMC6732822/) | [Schein et al. (2021)](https://www.jmcp.org/doi/full/10.18553/jmcp.2021.21290) | | [FDA (2023)](https://www.accessdata.fda.gov/scripts/cder/daf/index.cfm?event=BasicSearch.process) | |
| Neurology | Neuromyelitis Optica Spectrum Disorder | No | 41 | 4% | 5% | | 65.4 | 24.3 | 0.74 | 2.4 | Yes | [Mealy et al. (2012)](https://jamanetwork.com/journals/jamaneurology/fullarticle/1190816) | | | [Ada Noris Ferilli et al. (2022)](https://www.ncbi.nlm.nih.gov/pmc/articles/PMC8779266/) | [Royston et al. (2021)](https://www.ncbi.nlm.nih.gov/pmc/articles/PMC8571448/) | | [Aungsumart and Apiwattanakul (2020)](https://pdfs.semanticscholar.org/008c/e700c152613eba2dbce861b41d56c8a09cdf.pdf) | | [Aungsumart and Apiwattanakul (2020)](https://pdfs.semanticscholar.org/008c/e700c152613eba2dbce861b41d56c8a09cdf.pdf) | | [Mealy et al. (2019)](https://meridian.allenpress.com/ijmsc/article/21/3/129/12039/Assessment-of-Patients-with-Neuromyelitis-Optica) | [Cleveland Clinic (2022)](https://my.clevelandclinic.org/health/diseases/9858-neuromyelitis-optica-nmo) | | [FDA (2022)](https://www.accessdata.fda.gov/scripts/cder/daf/index.cfm?event=overview.process&varApplNo=761149) | |
| Hematology/Coagulation | Acquired Thrombotic Thrombocytopenic Purpura (aTTP) | No | 40 | 9% | 11% | | 44.3 | 4.3 | 0.74 | 0.5 | Yes | [Wun (2023)](https://emedicine.medscape.com/article/206598-overview#a5) | | | [Joly, Coppo, and Veyradier (2017)](https://ashpublications.org/blood/article/129/21/2836/36273/Thrombotic-thrombocytopenic-purpura) | [Agosti et al. (2019)](https://www.thrombosisresearch.com/article/S0049-3848(19)30455-4/fulltext) | | [Goshua et al. (2021)](https://www.ncbi.nlm.nih.gov/pmc/articles/PMC7918179/#:~:text=Using%20data%20from%20the%202,pay%20threshold%20of%20%24195%20300.) | | [Goshua et al. (2021)](https://www.ncbi.nlm.nih.gov/pmc/articles/PMC7918179/#:~:text=Using%20data%20from%20the%202,pay%20threshold%20of%20%24195%20300.) | | [Burns et al. (2018)](https://www.valueinhealthjournal.com/article/S1098-3015(18)36068-6/fulltext) | [Pollissard et al. (2021)](https://www.tandfonline.com/doi/pdf/10.1080/13696998.2021.1922262#:~:text=In%20the%20US%2C%20the%20reported,95%25%20of%20TTP%20cases11.) | | [FDA (2022)](https://www.accessdata.fda.gov/scripts/cder/daf/index.cfm?event=overview.process&varApplNo=761112) | |
| Neurology | Neurofibromatosis Type 1 (NF1) | No | 2 | 52% | 3% | | 77.9 | 75.9 | 0.73 | 33.3 | Yes | [NHS (2021)](https://www.nhs.uk/conditions/neurofibromatosis-type-1/symptoms/#:~:text=The%20spots%20can%20be%20present,the%20severity%20of%20the%20condition.) | | | [Madubata et al. (2015)](https://www.ncbi.nlm.nih.gov/pmc/articles/PMC4257895/pdf/nihms595045.pdf) | [Madubata et al. (2015)](https://www.ncbi.nlm.nih.gov/pmc/articles/PMC4257895/pdf/nihms595045.pdf) | | [NIH (2023)](https://www.ninds.nih.gov/health-information/disorders/neurofibromatosis) | | [Elbeltagy and Abbassy (2023)](https://link.springer.com/article/10.1007/s00381-023-06160-3) | | [Hamoy-Jimenez et al. (2020)](https://www.ncbi.nlm.nih.gov/pmc/articles/PMC7317053/#:~:text=Conclusions,quality%20of%20life%20in%20NF1.) | [JHM](https://www.hopkinsmedicine.org/health/conditions-and-diseases/neurofibromatosis/neurofibromatosis-type-1#:~:text=Neurofibromatosis%20type%201%20(also%20called,some%20people%20than%20in%20others.) | | [FDA (2024)](https://www.accessdata.fda.gov/scripts/cder/daf/index.cfm?event=overview.process&varApplNo=213756) | |
| Psychiatry | Schizophrenia | No | 25 | 0% | 31% | | 68.6 | 26.3 | 0.73 | 445.0 | No | [Solmi et al. (2021)](https://www.nature.com/articles/s41380-021-01161-7) | | | [Gochman, Miller, and Rapoport (2011)](https://www.ncbi.nlm.nih.gov/pmc/articles/PMC3289250/#:~:text=Childhood%2Donset%20schizophrenia%20(COS),many%20alternative%20diagnoses%20(ADs).) | [Lin et al. (2022)](https://link.springer.com/article/10.1186/s12888-022-04022-x/tables/1) | | [Hjorthoj et al. (2017)](https://www.sciencedirect.com/science/article/abs/pii/S2215036617300780) | | [Jin et al. (2020)](https://www.ncbi.nlm.nih.gov/pmc/articles/PMC7254180/) | | [Aceituno et al. (2021)](https://www.valueinhealthjournal.com/article/S1098-3015(20)32126-4/fulltext?_returnURL=https%3A%2F%2Flinkinghub.elsevier.com%2Fretrieve%2Fpii%2FS1098301520321264%3Fshowall%3Dtrue#supplementaryMaterial) | [NIH (2024)](https://www.nimh.nih.gov/health/statistics/schizophrenia#:~:text=Prevalence%20of%20Schizophrenia,-Precise%20prevalence%20estimates&text=A%20summary%20of%20currently%20available,between%200.25%25%20and%200.64%25.) | | [FDA (2023)](https://www.accessdata.fda.gov/scripts/cder/daf/index.cfm?event=overview.process&varApplNo=209500) | |
| Gastroenterology | Primary Biliary Cholangitis (PBC) | Yes | 50 | 0% | 27% | | 60.0 | 10.0 | 0.73 | 39.2 | Yes | [Chew et al (2018)](https://www.sciencedirect.com/science/article/pii/S254256841730017X) | | | [Gungabissoon et al (2022)](https://pmc.ncbi.nlm.nih.gov/articles/instance/9386220/bin/bmjgast-2021-000857supp002.pdf) | [Gungabissoon et al (2022)](https://pmc.ncbi.nlm.nih.gov/articles/instance/9386220/bin/bmjgast-2021-000857supp002.pdf) | | Calculation | | [Cleveland Clinic](https://my.clevelandclinic.org/health/diseases/17715-primary-biliary-cholangitis-pbc) | | [Wunsch et al (2022)](https://pmc.ncbi.nlm.nih.gov/articles/PMC10091761/) | [Lu et al (2018)](https://pubmed.ncbi.nlm.nih.gov/29277621/) | | [FDA OPD Listing](https://www.accessdata.fda.gov/scripts/opdlisting/oopd/) | |
| Infectious Disease (non viral) | Travelers' diarrhea | No | N/A | 9% | 37% | | 77.5 | N/A | 0.73 | 14783.8 | No | Assumption | | | [Shai, Schwartz, and O'Ryan (2016)](https://journals.lww.com/pidj/FullText/2016/06000/Travelers__Diarrhea_in_Children__What_Have_We.22.aspx) | [Molloy (2025)](https://www.theseniorlist.com/travel/statistics/#:~:text=Senior%20Travel%20Statistics%20in%202025,contributor%20to%20the%20travel%20industry.&text=Over%20the%20last%20year%2C%20interest,rates%20in%202023%20and%20beyond.) | | [NVSS (2025)](https://www.cdc.gov/nchs/data/nvsr/nvsr74/nvsr74-02.pdf) | | Assumption | | [Sturkenboom et al. (2021)](https://link.springer.com/article/10.1007/s11136-021-02995-y) | [Cleveland Clinic (2024)](https://my.clevelandclinic.org/health/diseases/7315-travelers-diarrhea) | | [FDA](https://www.accessdata.fda.gov/scripts/cder/daf/index.cfm?event=BasicSearch.process) | |
| Gastroenterology | Ulcerative Colitis | No | 30 | 5% | 13% | | 78.9 | 48.9 | 0.71 | 19.0 | No | [Quezada et al. (2012)](https://www.ncbi.nlm.nih.gov/pmc/articles/PMC3873098/pdf/nihms533891.pdf) | | | [Kappelman et al. (2007)](https://www.cghjournal.org/action/showPdf?pii=S1542-3565%2807%2900715-X) | [Shivashankar et al. (2017)](https://www.cghjournal.org/action/showPdf?pii=S1542-3565%2816%2931055-2) | | [Lynch et al. (2023)](https://www.ncbi.nlm.nih.gov/books/NBK459282/) | | Calculation | | [ICER et al. (2020)](https://icer.org/wp-content/uploads/2020/08/ICER_UC_Final_Evidence_Report_101620.pdf) | [Weisman et al. (2023)](https://pdf.sciencedirectassets.com/280666/1-s2.0-S2211335523X00035/1-s2.0-S2211335523000645/main.pdf?X-Amz-Security-Token=IQoJb3JpZ2luX2VjEIL%2F%2F%2F%2F%2F%2F%2F%2F%2F%2FwEaCXVzLWVhc3QtMSJHMEUCIQCP%2BmVI6Md%2FzEkoBswXMYb4L%2BB4i%2FMY1%2FfJvwR520kW2AIgcIHyHRP3Or5W2C8PgPqHGpc3gvCQ85kuendv8peSELUqswUISxAFGgwwNTkwMDM1NDY4NjUiDDvW21ZxmcyrcO%2FqYSqQBTZGqVBVXArC5VphkCOdpEEEHJamG8%2Byz0aSX8NO4PBDzdvLyJtCZ1ihtAX89tgZuQnkI3Gm3afzBkE5dLcgBwinK%2Fj89sU7fndjtKIMbgZiE9KVxWScT019lLTbGYbm37EYbUPbv2wJttFBONqxbE7x6a4yT12NRe%2BFOzjXb7bUfshOE9UzcVhQgmkbBqv3JtfmxKOU8LQ8GUy9ps9uXEUIgSwiEeIaTcXDdy8hE1Xa9fYcd9JLyfYCzdd%2BGK0sdO1%2FXhD6ueKsbwHON6JPYy%2FSMloL2KiJJ8b9QHJcvHNhhzsVSKsYv3ZlQUpzmFxGL1OJdFFl5lsDxglZBLNx%2FXDbV34o5%2BBA809bGcnzGa%2FJ7DUa%2B65VslEs6sH%2BAVDxhEru8MfenX1mZuEdhVbD4wPXNXnqK2xw9QnPzfLRjNZnYNDKZXjKbSTJgPfn%2BVlGmcjc4iKVusowwPQbpeATZOXRIGGQbl2m20tT1fyEtXYCeqnAPz0zlVXOpHh23UO2iZTkOZ%2FVpCwWhXne7P8bIKhxwX8tGIoFw9XP9H02RN6M8kHuarHf8sA47ONK7JXFk7jUaGekR8NzrwXRKLNeEleB%2FWZ1RWYeyTm%2FNGjCLKoNMrSXHogbTlDdaKP07NffQRB8dvA5onJK4zkytlntQIrq84mWSoR%2BevoWqgrje%2BQ3kD7%2B%2FuH2NTx0N9tRTeUx%2FbpHu0RTXSv2EgntZkzXCG0wG81UpM5lZ6MRbGqRyH5zwHRfa523%2BLj7SN6yT8jQdky6IkBC%2FgOkujgQtR8VCZRhb2kUPb8QAaPeqsz5HCoa1C7j1pjSqYv5RMVH9OflPS%2Fv5bsS0TlFqNuAjGAtEUJymabrqROWEhs9qSETAWGxMLKn760GOrEBGKdZRDuJ9xUFDzTZQtQmvg0UNteCxGt%2BwV5t0W9WDrA0IZnKdNqXRLjtRH%2BXDTApRTfLJbVl1mUvEDMITWFB%2Ftpo%2FhzsTNonq60Wf%2BoSa6L9mDXVHlnaOgY0CN8BIqlUJx7YMZio3XUznWPJZBVEGzqwv7psR%2B36fftPOYcr77rwLdYT4Ch7S9i10KItxNf6XZCNjYWWrjkYS2vvwkmL4Jx2NOXmq8LpVLCnbEM01lsO&X-Amz-Algorithm=AWS4-HMAC-SHA256&X-Amz-Date=20240201T181704Z&X-Amz-SignedHeaders=host&X-Amz-Expires=300&X-Amz-Credential=ASIAQ3PHCVTYURUD5VLP%2F20240201%2Fus-east-1%2Fs3%2Faws4_request&X-Amz-Signature=0334ca7d544bcae8ef7c1647cabc6a733d88725e8f629faeab9a64e1d00abfcc&hash=05c1a3cfb7daf4842d6fa1b075dd939ebfc1906c7fba38c1e790925ce4dc1b8f&host=68042c943591013ac2b2430a89b270f6af2c76d8dfd086a07176afe7c76c2c61&pii=S2211335523000645&tid=spdf-fae9f610-9d16-417e-83b9-6b130c12c278&sid=be92f8c13e5cb445fe8af2a-e218d94ca600gxrqa&type=client&tsoh=d3d3LnNjaWVuY2Vka) | | [FDA (2023)](https://www.accessdata.fda.gov/scripts/cder/daf/index.cfm?event=overview.process&varApplNo=216956) | |
| Rheumatology | Rheumatoid Arthritis | No | 60 | N/A | 50% | | 71.4 | 14.1 | 0.70 | 13.0 | No | [Gabriel et al. (1999)](https://onlinelibrary.wiley.com/doi/epdf/10.1002/1529-0131%28199904%2942%3A3%3C415%3A%3AAID-ANR4%3E3.0.CO%3B2-Z) | | | N/A | [Ni;sson et al. (2021)](https://www.tandfonline.com/doi/epdf/10.2147/OARRR.S306378?needAccess=true) | | [Chiu et al. (2021)](https://pubmed.ncbi.nlm.nih.gov/33295139/) | | [Yoshizawa et al. (1990)](https://pubmed.ncbi.nlm.nih.gov/2287966/) | | [ICER et al. (2019)](https://icer.org/wp-content/uploads/2020/10/ICER_RA_Final_Evidence_Report_and_Meeting_Summary_010820.pdf) | [Helmick et al. (2008)](https://onlinelibrary.wiley.com/doi/epdf/10.1002/art.23177) | | [FDA (2019)](https://www.accessdata.fda.gov/scripts/cder/daf/index.cfm?event=overview.process&varApplNo=211675) | |
| Endocrinology/Metabolism/Bone | Achondroplasia | Yes | 0 | 49% | 10% | | 66.4 | 66.4 | 0.70 | 5.0 | Yes | [Hoover-Fong et al. (2021)](https://www.sciencedirect.com/science/article/pii/S875632822100034X) | | | [Broder et al. (2022)](https://becarispublishing.com/doi/10.2217/cer-2021-0250#:~:text=In%202017%2C%20there%20were%201985,bed%20size%20(Table%202).) | [Broder et al. (2022)](https://becarispublishing.com/doi/10.2217/cer-2021-0250#:~:text=In%202017%2C%20there%20were%201985,bed%20size%20(Table%202).) | | [Pauli (2019)](https://ojrd.biomedcentral.com/articles/10.1186/s13023-018-0972-6) | | [Pauli (2019)](https://ojrd.biomedcentral.com/articles/10.1186/s13023-018-0972-6) | | [Murton et al. (2023)](https://www.ncbi.nlm.nih.gov/pmc/articles/PMC10427595/#:~:text=In%20addition%20to%20the%20high,achondroplasia%20has%20been%20largely%20symptomatic.) | [Bacino (2023)](https://www.uptodate.com/contents/achondroplasia#:~:text=Achondroplasia%20is%20the%20most%20common,receptor%203%20(FGFR3)%20gene.) | | [FDA (2023)](https://www.accessdata.fda.gov/scripts/cder/daf/index.cfm?event=overview.process&varApplNo=214938) | |
| Hematology/Coagulation | Cold Agglutinin Disease | No | 65 | 0% | 50% | | 83.9 | 6.8 | 0.70 | 4804.80 | Yes | [NORD (2023)](https://rarediseases.org/rare-diseases/cold-agglutinin-disease/#affected) | | | [Hansen et al. (2021)](https://air.unimi.it/retrieve/dfa8b9a9-d4b0-748b-e053-3a05fe0a3a96/American%20J%20Hematol%20-%202021%20-%20Hansen%20-%20Seasonal%20variation%20in%20the%20incidence%20of%20cold%20agglutinin%20disease%20in%20Norway%20%20Denmark%20%20and.pdf) | [NORD (2023)](https://rarediseases.org/rare-diseases/cold-agglutinin-disease/#affected) | | [Cleveland Clinic (2022)](https://my.clevelandclinic.org/health/diseases/23178-cold-agglutinin-disease) | | [Ito et al. (2023)](https://ashpublications.org/blood/article/142/Supplement%201/2316/502263/Cost-Effectiveness-of-Sutimlimab-Versus-Standard) | | [Roth et al. (2022)](https://www.ncbi.nlm.nih.gov/pmc/articles/PMC9463238/) | [NORD (2023)](https://rarediseases.org/rare-diseases/cold-agglutinin-disease/#affected) | | [FDA (2024)](https://www.accessdata.fda.gov/scripts/cder/daf/index.cfm?event=overview.process&varApplNo=761164) | |
| Endocrinology/Metabolism/Bone | Hypoparathyroidism | No | 35 | 3% | 37% | | 79.3 | 44.3 | 0.70 | 21.0 | Yes | [Mitchell et al. (2012)](https://pmc.ncbi.nlm.nih.gov/articles/PMC3513540/) | | | [Powers et al. (2013)](https://onlinelibrary.wiley.com/doi/10.1002/jbmr.2004) | [Powers et al. (2013)](https://onlinelibrary.wiley.com/doi/10.1002/jbmr.2004) | | [Parathyroid UK](https://parathyroiduk.org/recently-been-diagnosed-with-hypopara/) | | Calculation | | [Siggelkow et al. (2019)](https://pmc.ncbi.nlm.nih.gov/articles/PMC7027891/#cen14128-sec-0015) | [NORD (2024)](https://rarediseases.org/rare-diseases/hypoparathyroidism/#affected) | | FDA | |
| Psychiatry | Bipolar I Disorder | No | 25 | 3% | 25% | | 65.6 | 40.6 | 0.69 | 1000.0 | No | [NAMI (2017)](https://www.nami.org/About-Mental-Illness/Mental-Health-Conditions/Bipolar-Disorder#:~:text=The%20average%20age%2Dof%2Donset,of%20cases%20classified%20as%20severe.) | | | [BCH](https://www.childrenshospital.org/conditions/bipolar-disorder#:~:text=While%20not%20nearly%20as%20common,hospitalized%20for%20inpatient%20psychiatric%20treatment.)) | [Ljubic et al. (2021)](https://annals-general-psychiatry.biomedcentral.com/articles/10.1186/s12991-021-00367-x) | | [Miller (2022)](https://www.psychiatrictimes.com/view/premature-mortality-in-bipolar-disorder) | | [Miller (2022)](https://www.psychiatrictimes.com/view/premature-mortality-in-bipolar-disorder) | | [Dembeck et al. (2023)](https://annals-general-psychiatry.biomedcentral.com/articles/10.1186/s12991-023-00440-7) | [Pettigrew (2012)](https://www.sciencedirect.com/topics/nursing-and-health-professions/bipolar-i-disorder#:~:text=The%20prevalence%20of%20BPI%20in,%E2%80%936%25%20of%20the%20population.&text=The%20majority%20of%20adult%20patients,pediatric%20onset%2C%20including%20prepubertal%20episodes.) | | [FDA (2023)](https://www.accessdata.fda.gov/scripts/cder/daf/index.cfm?event=overview.process&varApplNo=213378) | |
| Neurology | Obstructive Sleep Apnea (OSA) | No | 36 | 5% | 20% | | 62.9 | 26.9 | 0.69 | 11711.7 | Yes | [Kales et al. (1985)](https://pubmed.ncbi.nlm.nih.gov/3998056/) | | | [Bseikri, Lo, and Guilleminault (2015)](https://link.springer.com/article/10.1007/s41030-015-0005-8) | [Cleveland Clinic (2022)](https://my.clevelandclinic.org/health/diseases/24443-obstructive-sleep-apnea-osa) | | [Pai (2023)](https://www.thecpapshop.com/blog/untreated-sleep-apnea-life-expectancy/) | | Calculation | | [Stepnowsky et al. (2019)](https://jcsm.aasm.org/doi/10.5664/jcsm.7624#d1e427) | [Ling (2023)](https://www.ncoa.org/adviser/sleep/sleep-apnea-statistics/#:~:text=Approximately%2039%20million%20U.S.%20adults,up%20to%2094%25%20of%20patients.) | | [FDA (2023)](https://www.accessdata.fda.gov/scripts/cder/daf/index.cfm?event=overview.process&varApplNo=211230) | |
| Endocrinology/Metabolism/Bone | Alagille Syndrome | No | 0 | N/A | N/A | | 62.3 | 62.3 | 0.69 | 3.3 | Yes | [Saleh, Kamath, & Chitayat (2016)](https://www.tandfonline.com/doi/full/10.2147/TACG.S86420) | | | N/A | N/A | | [Kharmach, Borahma, and Ajana (2023)](https://eglj.springeropen.com/articles/10.1186/s43066-023-00298-0) | | [Kharmach, Borahma, and Ajana (2023)](https://eglj.springeropen.com/articles/10.1186/s43066-023-00298-0) | | [Quadrado et al. (2022)](https://www.ispor.org/docs/default-source/euro2022/quadrado-l-2022-ispor-patient-and-caregiver-burden-associated-with-caring-for-a-child-with-algs-pdf.pdf?sfvrsn=7b342bf1_0) | [ALF (2023)](https://liverfoundation.org/liver-diseases/pediatric-liver-information-center/pediatric-liver-disease/alagille-syndrome/) | | [FDA (2023)](https://www.accessdata.fda.gov/scripts/cder/daf/index.cfm?event=overview.process&varApplNo=214662) | |
| Endocrinology/Metabolism/Bone | Tenosynovial Giant Cell Tumor (TGCT) | No | 38 | 5% | 36% | | 76.4 | 38.9 | 0.68 | 4.4 | Yes | [NORD (2023)](https://rarediseases.org/rare-diseases/tenosynovial-giant-cell-tumor/#affected) | | | [Mastboom et al. (2017)](https://www.ncbi.nlm.nih.gov/pmc/articles/PMC5694816/) | [Ehrenstein et al. (2017)](https://www.jrheum.org/content/jrheum/early/2017/07/26/jrheum.160816.full.pdf) | | [Stacchiotti et al. (2022)](https://www.cancertreatmentreviews.com/article/S0305-7372(22)00160-8/fulltext) | | N/A | | [Palmerini et al. (2023)](https://academic.oup.com/oncolo/article/28/6/e425/7069289) | [Delveinsight (2023)](https://www.delveinsight.com/report-store/tenosynovial-giant-cell-tumors-tsgcts-epidemiology-forecast) | | [FDA (2023)](https://www.accessdata.fda.gov/scripts/cder/daf/index.cfm?event=overview.process&varApplNo=211810) | |
| Ophthalmology | Wet Age-Related Macular Degeneration (AMD) | No | 55 | 0% | 93% | | 74.2 | 9.9 | 0.67 | 447.4 | No | [Zimlich (2023)](https://www.healthline.com/health/eye-health/macular-degeneration-age-of-onset) | | | [Prall (2023)](https://emedicine.medscape.com/article/1226030-overview#a7) | [Korva-Gurung et al. (2023)](https://www.ncbi.nlm.nih.gov/pmc/articles/PMC10262804/#:~:text=49%25%20of%20the%20nAMD%20patients,under%2074%20years%20of%20age.) | | [Bandello, Lafuma, and Berdeaux (2007)](https://iovs.arvojournals.org/article.aspx?articleid=2163913) | | [Stein et al. (2013)](https://www.ncbi.nlm.nih.gov/pmc/articles/PMC3797829/#:~:text=Over%2020%20years%2C%20the%20expected,receiving%20these%20treatments%20were%206.66%2C) | | [Hernandez et al. (2018)](https://www.jmcp.org/doi/10.18553/jmcp.2018.24.7.608) | [Rein et al. (2022)](https://jamanetwork.com/journals/jamaophthalmology/fullarticle/2797921?guestAccessKey=a59b3a27-b0ca-4203-bc5a-87c884b78a5c&utm_source=For_The_Media&utm_medium=referral&utm_campaign=ftm_links&utm_content=tfl&utm_term=110322) | | [FDA (2023)](https://www.accessdata.fda.gov/scripts/cder/daf/index.cfm?event=overview.process&varApplNo=761235) | |
| Hematology/Coagulation | Hemophilia B | No | 1 | 46% | 6% | | 70.4 | 69.4 | 0.67 | 3.7 | Yes | [Kulkarni et al. (2009)](https://pubmed.ncbi.nlm.nih.gov/19637999/) | | | [CDC (2023)](https://www.cdc.gov/ncbddd/hemophilia/communitycounts/registry-report-males/age.html) | [CDC (2023)](https://www.cdc.gov/ncbddd/hemophilia/communitycounts/registry-report-males/age.html) | | [Hassan et al. (2021)](https://www.ncbi.nlm.nih.gov/pmc/articles/PMC7986360/pdf/JTH-19-645.pdf) | | Calculation | | [Carroll et al. (2019)](https://www.dovepress.com/getfile.php?fileID=50536) | [Soucie et al. (2020)](https://www.ncbi.nlm.nih.gov/pmc/articles/PMC8117262/) | | [FDA (2022)](https://www.fda.gov/media/163466/download?attachment) | |
| Infectious Disease (non viral) | Community-acquired Bacterial Pneumonia (CABP) | No | 46 | 13% | 31% | | 66.5 | 20.8 | 0.66 | 248.0 | No | [Divino et al. (2020)](https://becarispublishing.com/doi/10.2217/cer-2019-0151?url_ver=Z39.88-2003&rfr_id=ori%3Arid%3Acrossref.org&rfr_dat=cr_pub++0pubmed) | | | [Yun et al. (2022)](https://journals.lww.com/pidj/abstract/2022/05000/clinical_characteristics_and_etiology_of.4.aspx) | [Sato et al. (2013)](https://link.springer.com/article/10.1007/s40258-013-0026-0) | | [Ajayi et al. (2017)](https://www.amjmedsci.com/article/S0002-9629(17)30127-1/fulltext) | | Calculation | | [Edwards et al. (2012)](https://pubmed.ncbi.nlm.nih.gov/21243514/) | [Regunath et al. (2022)](https://www.ncbi.nlm.nih.gov/books/NBK430749/#:~:text=In%20the%20United%20States%2C%20the,to%20the%20intensive%20care%20unit.) | | [FDA (2019)](https://www.accessdata.fda.gov/scripts/cder/daf/index.cfm?event=overview.process&varApplNo=211672) | |
| Infectious Disease (non viral) | Hospital-Acquired Bacterial Pneumonia | No | 58 | 9% | 26% | | 66.5 | 8.3 | 0.66 | 1600.0 | No | [Tabek et al. (2019)](https://pubmed.ncbi.nlm.nih.gov/31228511/) | | | [Giuliano et al. (2023)](https://www.ajicjournal.org/action/showPdf?pii=S0196-6553%2822%2900499-0) | [Giuliano et al. (2023)](https://www.ajicjournal.org/action/showPdf?pii=S0196-6553%2822%2900499-0) | | [Ajayi et al. (2017)](https://www.amjmedsci.com/article/S0002-9629(17)30127-1/fulltext) | | Calculation | | [Edwards et al. (2012)](https://pubmed.ncbi.nlm.nih.gov/21243514/) | [Giuliano et al. (2018)](https://www.ajicjournal.org/article/S0196-6553(17)31056-8/pdf) | | [FDA (2023)](https://www.accessdata.fda.gov/scripts/cder/daf/index.cfm?event=overview.process&varApplNo=216974) | |
| Cardiovascular Disease | Heart Failure | No | 69 | 0% | 69% | | 74.4 | 5.5 | 0.66 | 1861.9 | No | [Tromp et al. (2021)](https://www.bmj.com/content/372/bmj.n461) | | | [Das (2020)](https://www.ncbi.nlm.nih.gov/pmc/articles/PMC6069285/#:~:text=Incidence%20and%20Prevalence&text=Heart%20failure%2Drelated%20hospitalization%20occurs,children%20annually%20in%20the%20US.) | [Tromp et al. (2021)](https://www.bmj.com/content/372/bmj.n461) | | [Alter et al. (2012)](https://www.ncbi.nlm.nih.gov/pmc/articles/PMC3515002/) | | Calculation | | [Luo et al. (2011)](https://www.sciencedirect.com/science/article/abs/pii/S1877131911000279) | [CDC (2023)](https://www.cdc.gov/heartdisease/heart_failure.htm) | | [FDA (2023)](https://www.accessdata.fda.gov/scripts/cder/daf/index.cfm?event=overview.process&varApplNo=216203) | |
| Dermatology | Plaque Psoriasis | No | 31 | 11% | 16% | | 70.0 | 39.5 | 0.66 | 99.9 | No | [Fatema et al. (2021)](https://www.ncbi.nlm.nih.gov/pmc/articles/PMC8906303/) | | | [Alakbarov et al. (2021)](https://jag.journalagent.com/turkderm/pdfs/TURKDERM_55_4_193_198.pdf) | [Alakbarov et al. (2021)](https://jag.journalagent.com/turkderm/pdfs/TURKDERM_55_4_193_198.pdf) | | [Chiu et al. (2023)](https://www.ncbi.nlm.nih.gov/pmc/articles/PMC10155019/) | | Calculation | | [ICER et al. (2018)](https://icer.org/wp-content/uploads/2020/10/ICER_Psoriasis_Update_Final_Evidence_Report_10042018.pdf) | [Armstrong et al. (2021)](https://watermark.silverchair.com/jamadermatology_armstrong_2021_oi_210025_1628612398.46283.pdf?token=AQECAHi208BE49Ooan9kkhW_Ercy7Dm3ZL_9Cf3qfKAc485ysgAAA3cwggNzBgkqhkiG9w0BBwagggNkMIIDYAIBADCCA1kGCSqGSIb3DQEHATAeBglghkgBZQMEAS4wEQQMa4Jm_RVrJH6ncjPpAgEQgIIDKpepue_0MFpclIv5dUzwsS8F4hi4wWCrXgE2o36ig1Nb9ftn52jNGxN3lzwLPu1oFzKuYXYqaIUwPTaMNgt2NAVuvOeUUwZFLp5FtnLLUA2sRdDU5sO3hxo6qmHLSsN_Lq7Zt2wfwBrGPYQ_wDbxlgiVcaeVG4-CSoqy5TvPWae5EwKlGxepWyNlBpCnatnEjo4nt45vhxF7qpk_276TTMI1OG5kP5HYGcuKxyyWOfS-YCX_n9EexcSTVzwPc9zfCO56m17JpQHYGJ5zQqyLvRbaK28Mfejp5HZx2Z5R1JN2xDEUAGhqxmRAL8sJvNjyIOhxXm_sj7Vp61_Q4n59qDa7yYP_oNtA2YQK5-A-EHmbhBQVBbiavtnnPgNGByZRcjZ78U0pujJ9ddLiPwt7zcSwnCgle516FNSNmgmcm5VwVmKqVbBJwqXOXLPRYMCmPnrh31hyR1uYS0PjquVqIkGNBUw1CP2oXXVBuknbT7R2Lz115SNq9ZY8mE5Y1ckJhFj5y3ASx6NWxfuzskA6EbbqJUQ6Em2E8jlLloDu4sIgBFjeLbxAzgqv7wZ5zoracJ97lS9nAr3GnCjZXj1ON2a93YsEYRKseRYdwxiBtwtiTsmhuVnZhFhaFg24yzOF80qftfYMAbAANulA7_4kqUHjgRM9mMs6U1ITdKhx0_kXzO8YUDJSPliMpq4qiX8HRNEF6IeofsLjZEOt84c2JSJI9uhbKx_sPrqZSSH41XCn-YGzhTrp5KuV6abHsCvrEwrm9teb9uucgOZcUybgZxcGPqsq6U-N56pC1OOF-XqYQcJ5E4PZf7TZezQt-kOjXEtmZoJM3eUG8XmNGKlG0eqkivVirILuaJ-rMp8vuBMP1D1QFKM4Xtq8YX9Mo9cdpLp_v7n2UjXUsFhDP9wIHWpJ_g8cgA8k-lK84Z3-_GeltmxkWlqHbRQsWWiL6aA3jcGQhtIpqa07tf_3bQZdsvMsIxafrnqMiUh7H8gBdQTjhbmZdcUs9pygDTpo9OFwnSO27ngP8HTlhUJQ30FJrcP2y9eZ9Z4gBk3ULr5j5e-p7qAGQRZERK0Cxg) | | [FDA (2023)](https://www.accessdata.fda.gov/scripts/cder/daf/index.cfm?event=overview.process&varApplNo=761151) | |
| Endocrinology/Metabolism/Bone | Severe Hypoglycemia | No | 54 | 7% | 76% | | 57.6 | 3.6 | 0.66 | 276.8 | No | [Bajpai et al. (2021)](https://pubmed.ncbi.nlm.nih.gov/33645242/) | | | [Bajpai et al. (2021)](https://pubmed.ncbi.nlm.nih.gov/33645242/) | [Lee et al. (2021)](https://www.sciencedirect.com/science/article/pii/S0168822721003065#t0005) | | [Klein (2023)](https://www.ajmc.com/view/severe-hypoglycemia-requiring-hospitalization-increases-premature-death-risk-in-people-with-t1d) | | Calculation | | [Shao et al. (2019)](https://www.ncbi.nlm.nih.gov/pmc/articles/PMC7220804/) | [Bajpai et al. (2021)](https://pubmed.ncbi.nlm.nih.gov/33645242/) | | [FDA (2021)](https://www.accessdata.fda.gov/scripts/cder/daf/index.cfm?event=overview.process&varApplNo=214231) | |
| Hematology/Coagulation | Neutropenia | No | 45 | 3% | 46% | | 65.8 | 20.6 | 0.66 | 10660.7 | No | [Tice et al. (2022)](https://icer.org/wp-content/uploads/2021/08/ICER_Neutropenia_Evidence-Report_033122.pdf) | | | [Flanigan et al. (2023)](https://www.researchsquare.com/article/rs-3706613/v1) | [Flanigan et al. (2023)](https://www.researchsquare.com/article/rs-3706613/v1) | | [Shitara et al. (2010)](https://www.annalsofoncology.org/article/S0923-7534(19)39254-3/fulltext) | | [Akpo et al. (2017)](https://www.frontiersin.org/articles/10.3389/fphar.2017.00614/full) | | [Akpo et al. (2017)](https://www.frontiersin.org/articles/10.3389/fphar.2017.00614/full) | [Zhou et al. (2023)](https://www.ncbi.nlm.nih.gov/pmc/articles/PMC10308693/#:~:text=The%20estimated%20prevalence%20of%20neutropenia,individuals%20in%20the%20United%20States.) | | [FDA (2023)](https://www.accessdata.fda.gov/scripts/cder/daf/index.cfm?event=overview.process&varApplNo=761134) | |
| Hematology | Methemoglobinemia | Yes, verified | 33 | 36% | 16% | | 79.1 | 46.1 | 0.66 | 0.9 | Yes | [Gao, Basri, and Tran (2022)](https://www.sciencedirect.com/science/article/abs/pii/S1473050221002998) | | | [Belzer and Krasowski (2024)](https://www.sciencedirect.com/science/article/pii/S2214750024000283?via%3Dihub#sec0030) | [Ash-Bernal, Wise, and Wright (2004)](https://journals.lww.com/md-journal/fulltext/2004/09000/acquired_methemoglobinemia__a_retrospective_series.1.aspx) | | [Khanapara et al. (2023)](https://emedicine.medscape.com/article/204178-overview#showall) | | Calculation | | [Panepinto (2012)](https://ashpublications.org/hematology/article/2012/1/284/83767/Health-related-quality-of-life-in-patients-with) | [Chowdry et al. (2013)](https://jamanetwork.com/journals/jamainternalmedicine/fullarticle/1673755) | | FDA | |
| Infectious Disease (viral) | Post-transplant Cytomegalovirus (CMV) | No | 55 | 1% | 23% | | 59.7 | 4.7 | 0.65 | 5.6 | Yes | [Cleveland Clinic (2022)](https://my.clevelandclinic.org/health/treatments/22537-kidney-transplant) | | | [Seale et al. (2008)](https://journals.sagepub.com/doi/full/10.4137/VRT.S920) | [Seale et al. (2008)](https://journals.sagepub.com/doi/full/10.4137/VRT.S920) | | [Sepassi et al. (2023)](https://www.ncbi.nlm.nih.gov/pmc/articles/PMC10169956/pdf/41669_2023_Article_398.pdf) | | [Sepassi et al. (2023)](https://www.ncbi.nlm.nih.gov/pmc/articles/PMC10169956/pdf/41669_2023_Article_398.pdf) | | [Sepassi et al. (2023)](https://www.ncbi.nlm.nih.gov/pmc/articles/PMC10169956/pdf/41669_2023_Article_398.pdf) | [Roche (2024)](https://www.roche.com/stories/cmv-in-organ-transplantation#:~:text=Between%2020%25%20and%2060%25%20of,may%20be%20difficult%20to%20control.) | | [FDA (2023)](https://www.accessdata.fda.gov/scripts/cder/daf/index.cfm?event=overview.process&varApplNo=215596) | |
| Hematology/Coagulation | Active Anti-neutrophil Cytoplasmic Autoantibody-associated Vasculitis (Granulomatosis with Polyangiitis) | No | 45 | 4% | 27% | | 58.0 | 9.4 | 0.65 | 3.0 | Yes | [Pace et al. (2020)](https://www.ncbi.nlm.nih.gov/pmc/articles/PMC6849427/) | | | [Panupattanapong et al. (2018)](https://acrjournals.onlinelibrary.wiley.com/doi/abs/10.1002/art.40577) | [Pearce et al. (2017)](https://academic.oup.com/rheumatology/article/56/4/589/2738663) | | [Tufan & Tekkarismaz (2021)](https://archivesofrheumatology.org/full-text/1222) | | [Macia et al. (2024)](https://www.tandfonline.com/doi/pdf/10.1080/14737167.2023.2297790) | | [Macia et al. (2024)](https://www.tandfonline.com/doi/pdf/10.1080/14737167.2023.2297790) | [Tracy et al. (2023)](https://emedicine.medscape.com/article/332622-overview#a6) | | [FDA (2022)](https://www.accessdata.fda.gov/scripts/cder/daf/index.cfm?event=overview.process&varApplNo=214487) | |
| Hematology/Coagulation | Active Anti-neutrophil Cytoplasmic Autoantibody-associated Vasculitis (Microscopic Polyangiitis) | No | 50 | N/A | N/A | | 59.4 | 9.4 | 0.65 | 1.6 | Yes | [Hashmi, Jain, & Tiwari (2023)](https://www.ncbi.nlm.nih.gov/books/NBK531484/) | | | N/A | N/A | | [Macia et al. (2024)](https://www.tandfonline.com/doi/pdf/10.1080/14737167.2023.2297790) | | [Macia et al. (2024)](https://www.tandfonline.com/doi/pdf/10.1080/14737167.2023.2297790) | | [Macia et al. (2024)](https://www.tandfonline.com/doi/pdf/10.1080/14737167.2023.2297790) | [Berti et al. (2017)](https://www.ncbi.nlm.nih.gov/pmc/articles/PMC5711593/) | | [FDA (2022)](https://www.accessdata.fda.gov/scripts/cder/daf/index.cfm?event=overview.process&varApplNo=214487) | |
| Immunomodulators | Lupus Nephritis (LN) | No | 29 | 11% | 26% | | 63.0 | 33.9 | 0.65 | 30.9 | No | [Feldman et al. (2018)](https://www.ncbi.nlm.nih.gov/pmc/articles/PMC5826885/) | | | [Hocaoglu et al. (2023)](https://pubmed.ncbi.nlm.nih.gov/36227575/) | [Gandhi et al. (2013)](https://acrabstracts.org/abstract/prevalence-of-systemic-lupus-erythematosus-and-lupus-nephritis-in-the-united-states-analysis-of-commercial-and-public-insurance-billing-data/) | | [Lerang et al. (2014)](https://journals.sagepub.com/doi/10.1177/0961203314551083) | | Calculation | | [ICER et al. (2021)](https://icer.org/wp-content/uploads/2020/11/ICER_Lupus-Nephritis_Final-Evidence-Report_05182022.pdf) | [Pryor et al. (2020)](https://www.ncbi.nlm.nih.gov/pmc/articles/PMC8171807/pdf/nihms-1629452.pdf) | | [FDA (2021)](https://www.accessdata.fda.gov/scripts/cder/daf/index.cfm?event=overview.process&varApplNo=213716) | |
| Immunomodulators | Severe Systemic Lupus Erythematosus (SLE) | No | 39 | 18% | 6% | | 60.0 | 17.5 | 0.65 | 72.8 | No | [Somers et al. (2014)](https://acrjournals.onlinelibrary.wiley.com/doi/epdf/10.1002/art.38238) | | | [Massias et al. (2019)](https://journals.sagepub.com/doi/full/10.1177/0961203320909156) | [Furst et al. (2012)](https://journals.sagepub.com/doi/abs/10.1177/0961203312463110) | | [Falasinnu et al. (2017)](https://www.ncbi.nlm.nih.gov/pmc/articles/PMC5695737/pdf/jwh.2017.6334.pdf) | | [ICER et al. (2021)](https://icer.org/wp-content/uploads/2020/11/ICER_Lupus-Nephritis_Final-Evidence-Report_05182022.pdf) | | [ICER et al. (2021)](https://icer.org/wp-content/uploads/2020/11/ICER_Lupus-Nephritis_Final-Evidence-Report_05182022.pdf) | [Izmirly et al. (2021)](https://www.ncbi.nlm.nih.gov/pmc/articles/PMC8169527/pdf/nihms-1657922.pdf) | | [FDA (2021)](https://www.accessdata.fda.gov/scripts/cder/daf/index.cfm?event=BasicSearch.process) | |
| Infectious Disease (non viral) | Chagas Disease | Yes | 37 | 0% | 68% | | 71.8 | 34.8 | [0.65](https://www.sciencedirect.com/science/article/pii/S2212109921001369) | 86.5 | Yes | [Abbott et al. (2022)](https://www.cdc.gov/mmwr/volumes/71/wr/mm7110a2.htm) | | | [Navarro et al. (2022)](https://www.sciencedirect.com/science/article/pii/S1477893922000308) | [Irish et al. (2022)](https://www.ncbi.nlm.nih.gov/pmc/articles/PMC9239882/) | | [EHJ (2009)](https://academic.oup.com/eurheartj/article-pdf/30/17/2063/17890228/ehp277.pdf) | | [Calculated](https://www.thelancet.com/pdfs/journals/langlo/PIIS2214-109X(17)30073-6.pdf) | | [Lins, Tura, and Kasal (2021)](https://www.sciencedirect.com/science/article/pii/S2212109921001369) | [Irish et al. (2022)](https://www.ncbi.nlm.nih.gov/pmc/articles/PMC9239882/#:~:text=Our%20estimates%20indicate%20that%20288%2C000,108%20congenital%20infections%20occur%20annually.) | | [FDA (2023)](https://www.accessdata.fda.gov/scripts/cder/daf/index.cfm?event=overview.process&varApplNo=213464) | |
| Endocrinology/Metabolism/Bone | Cushing's Disease | No | 42 | 5% | 32% | | 61.3 | 5.0 | 0.65 | 62.0 | Yes | [Burton et al. (2016)](https://www.ncbi.nlm.nih.gov/pmc/articles/PMC4799236/pdf/11102_2015_Article_695.pdf) | | | [Burton et al. (2016)](https://www.ncbi.nlm.nih.gov/pmc/articles/PMC4799236/pdf/11102_2015_Article_695.pdf) | [Akirov et al. (2022)](https://link.springer.com/article/10.1007/s11102-022-01292-2) | | [Lambert et al. (2013)](https://www.ncbi.nlm.nih.gov/pmc/articles/PMC3590483/) | | [Lambert et al. (2013)](https://www.ncbi.nlm.nih.gov/pmc/articles/PMC3590483/) | | [Schmidt et al. (2023)](https://www.endocrine-abstracts.org/ea/0090/ea0090ep68) | [Broder et al. (2014)](https://link.springer.com/article/10.1007/s11102-014-0569-6) | | [FDA (2020)](https://www.accessdata.fda.gov/scripts/cder/daf/index.cfm?event=overview.process&varApplNo=212801) | |
| Infectious Disease (viral) | HIV-1 Infection | No | 40 | 16% | 5% | | 75.6 | 35.2 | 0.65 | 10.6 | No | [Asher et al. (2016)](https://www.ncbi.nlm.nih.gov/pmc/articles/PMC4706254/#:~:text=The%20mean%20age%20of%20the,years%3B%20P%20%3D%200.01).) | | | [CDC (2019)](https://www.cdc.gov/hiv/pdf/library/reports/surveillance/cdc-hiv-surveillance-report-2018-updated-vol-32.pdf) | [CDC (2019)](https://www.cdc.gov/hiv/pdf/library/reports/surveillance/cdc-hiv-surveillance-report-2018-updated-vol-32.pdf) | | [Trickey et al. (2023)](https://www.thelancet.com/action/showPdf?pii=S2352-3018%2823%2900028-0) | | Calculation | | [Whitham et al. (2020)](https://www.ncbi.nlm.nih.gov/pmc/articles/PMC7432967/pdf/10.1177_2381468320936219.pdf) | [CDC (2019)](https://www.cdc.gov/hiv/pdf/library/reports/surveillance/cdc-hiv-surveillance-report-2018-updated-vol-32.pdf) | | [FDA (2022)](https://www.accessdata.fda.gov/scripts/cder/daf/index.cfm?event=overview.process&ApplNo=215974) | |
| Neurology | Childhood cerebral adrenoleukodystrophy (CCALD) | Yes | 7 | 100% | 0% | | 12.3 | 5.3 | 0.64 | 0.2 | Yes | [ALDConnect](https://aldconnect.org/what-is-ald/cerebral-ald/) | | | Assumption | Assumption | | [Sevin et al. (2023)](https://pmc.ncbi.nlm.nih.gov/articles/PMC10416383/#Sec3) | | Calculation | | [Paramore et al. (2022)](https://www.valueinhealthjournal.com/article/S1098-3015(21)02962-4/fulltext) | [BCH](https://www.childrenshospital.org/conditions/adrenoleukodystrophy-ald#:~:text=It%20occurs%20in%20about%201,highly%20unlikely%20to%20develop%20CALD.) | | [FDA OPD Listing](https://www.accessdata.fda.gov/scripts/opdlisting/oopd/) | |
| Neurology | Lambert-Eaton myasthenic syndrome (LEMS) | No | 54 | 0% | 61% | | 59.6 | 5.8 | 0.64 | 0.1 | Yes | [Jayarangaiah, Lui, and Kariyanna (2023)](https://www.ncbi.nlm.nih.gov/books/NBK507891/) | | | [NORD (2023)](https://rarediseases.org/rare-diseases/lambert-eaton-myasthenic-syndrome/#complete-report) | [Lehnerer et al. (2024)](https://link.springer.com/article/10.1007/s00415-024-12206-6#Sec19) | | Calculation | | [Lipka et al. (2020)](https://pmc.ncbi.nlm.nih.gov/articles/PMC7080283/) | | [Lipka et al. (2020)](https://pmc.ncbi.nlm.nih.gov/articles/PMC7080283/) | [NORD (2023)](https://rarediseases.org/rare-diseases/lambert-eaton-myasthenic-syndrome/#complete-report) | | FDA | |
| Dermatology | Pustular Psoriasis | No | 48 | 2% | 49% | | 70.0 | 22.2 | 0.63 | 9.0 | Yes | [Zheng et al. (2022)](https://www.ncbi.nlm.nih.gov/pmc/articles/PMC8801400/pdf/40257_2021_Article_664.pdf) | | | [Feldman et al. (2021)](https://www.avancesenppg.com/arxius/imatgesbutlleti/Feldman-SR-WPPAC-2021-Poster-35209.pdf) | [Feldman et al. (2021)](https://www.avancesenppg.com/arxius/imatgesbutlleti/Feldman-SR-WPPAC-2021-Poster-35209.pdf) | | [Chiu et al. (2023)](https://www.ncbi.nlm.nih.gov/pmc/articles/PMC10155019/) | | Calculation | | [Lebwohl et al. (2022)](https://journals.sagepub.com/doi/epub/10.1177/24755303221079814?src=getftr) | [Feldman et al. (2021)](https://www.avancesenppg.com/arxius/imatgesbutlleti/Feldman-SR-WPPAC-2021-Poster-35209.pdf) | | [FDA (2022)](https://www.accessdata.fda.gov/scripts/cder/daf/index.cfm?event=overview.process&varApplNo=761244) | |
| Urologic | Uncomplicated Urinary Tract Infections (uUTI) | No | 26 | 10% | 32% | | 78.6 | 52.6 | 0.63 | 5555.0 | No | [Bono et al (2025)](https://www.ncbi.nlm.nih.gov/books/NBK470195/#:~:text=Approximately%2010%25%20of%20women%20experience,.%5B8%5D%5B9%5D) | | | [Urology Care Foundation](https://www.urologyhealth.org/healthy-living/urologyhealth-extra/magazine-archives/summer-2016/understanding-utis-across-the-lifespan) | [Medina et al (2019)](https://pmc.ncbi.nlm.nih.gov/articles/PMC6502976/#:~:text=UTI%20epidemiology-,Uncomplicated%20UTIs,4) | | Assumption | | Calculation | | [Thompson et al (2023)](https://pmc.ncbi.nlm.nih.gov/articles/PMC9891499/) | [Medina et al (2019)](https://pmc.ncbi.nlm.nih.gov/articles/PMC6502976/#:~:text=UTI%20epidemiology-,Uncomplicated%20UTIs,4) | | FDA OPD Listing | |
| Hematology/Coagulation | Beta Thalassemia | No | 1 | 45% | 0% | | 37.0 | 22.1 | 0.62 | 1.0 | Yes | [Langer (2023)](https://www.ncbi.nlm.nih.gov/books/NBK1426/) | | | [Beaudoin et al. (2022)](https://icer.org/wp-content/uploads/2021/11/ICER_Beta-Thalassemia_Final-Report_071922.pdf) | Assumption due to LE set at 37 | | [Beaudoin et al. (2022)](https://icer.org/wp-content/uploads/2023/09/ICER_Beta-Thalassemia_Final-Report_12-Month_Update.pdf) | | [Beaudoin et al. (2022)](https://icer.org/wp-content/uploads/2023/09/ICER_Beta-Thalassemia_Final-Report_12-Month_Update.pdf) | | [Beaudoin et al. (2022)](https://icer.org/wp-content/uploads/2023/09/ICER_Beta-Thalassemia_Final-Report_12-Month_Update.pdf) | [NORD (2023)](https://rarediseases.org/rare-diseases/thalassemia-major/#affected) | | [FDA (2022)](https://www.fda.gov/media/160994/download?attachment) | |
| Dermatology | Atopic Dermatitis | No | 0 | 37% | 11% | | 77.5 | 77.1 | 0.61 | 4955.0 | No | [Avena-Woods (2017)](https://www.ajmc.com/view/overview-of-atopic-dermatitis-article) | | | [NEA](https://nationaleczema.org/research/eczema-facts/) | [Elsawi et al. (2022)](https://jamanetwork.com/journals/jamadermatology/fullarticle/2793426#:~:text=Results%20Among%201065%20survey%20respondents,881%20(83%25)%20were%20women.) | | [Fasseeh et al. (2022)](https://www.ncbi.nlm.nih.gov/pmc/articles/PMC9674816/) | | [Atlas et al. (2021)](https://icer.org/wp-content/uploads/2023/02/Atopic-Dermatitis_Final-Evidence-Report_Unmasked_02272023.pdf) | | [Atlas et al. (2021)](https://icer.org/wp-content/uploads/2023/02/Atopic-Dermatitis_Final-Evidence-Report_Unmasked_02272023.pdf) | [AAFA (2018)](https://aafa.org/wp-content/uploads/2022/08/Atopic-Dermatitis-in-America-Study-Overview.pdf) | | [FDA (2023)](https://www.accessdata.fda.gov/scripts/cder/daf/index.cfm?event=overview.process&varApplNo=213871) | |
| Psychiatry | Major Depressive Disorder (MDD) | No | 33 | 4% | 14% | | 66.8 | 20.6 | 0.60 | 210.0 | No | [The Recovery Village (2023)](https://www.therecoveryvillage.com/mental-health/major-depressive-disorder/major-depression-statistics/) | | | [CDC (2023)](https://www.cdc.gov/childrensmentalhealth/features/anxiety-depression-children.html) | [Lee et al. (2023)](https://www.cdc.gov/mmwr/volumes/72/wr/mm7224a1.htm#T1_down) | | [Blue Cross (2018)](https://www.bcbs.com/sites/default/files/file-attachments/health-of-america-report/HoA_Major_Depression_Report.pdf) | | [ICER et al. (2019)](https://icer.org/wp-content/uploads/2020/10/ICER_TRD_Final_Evidence_Report_062019.pdf) | | [ICER et al. (2019)](https://icer.org/wp-content/uploads/2020/10/ICER_TRD_Final_Evidence_Report_062019.pdf) | [NIH (2023)](https://www.nimh.nih.gov/health/statistics/major-depression) | | [FDA (2023)](https://www.accessdata.fda.gov/scripts/cder/daf/index.cfm?event=overview.process&varApplNo=021164) | |
| Infectious Disease (non viral) | Candidemia/Invasive Candidiasis | No | 59 | 5% | 40% | | 66.1 | 4.5 | 0.60 | 9.0 | Yes | [Toda et al. (2019)](https://www.cdc.gov/mmwr/volumes/68/ss/pdfs/ss6808a1-H.pdf) | | | [Ricotta et al. (2021)](https://www.ncbi.nlm.nih.gov/pmc/articles/PMC8030726/) | [Ricotta et al. (2021)](https://www.ncbi.nlm.nih.gov/pmc/articles/PMC8030726/) | | [Masterson et al. (2013)](https://www.tandfonline.com/doi/full/10.3111/13696998.2013.839948) | | [Chen et al. (2018)](https://www.tandfonline.com/doi/full/10.1080/13696998.2017.1417312) | | [Chen et al. (2018)](https://www.tandfonline.com/doi/full/10.1080/13696998.2017.1417312) | [CDC (2023)](https://www.cdc.gov/fungal/diseases/candidiasis/invasive/statistics.html#:~:text=Invasive%20candidiasis%20outbreaks-,How%20common%20is%20invasive%20candidiasis%3F,infections%20in%20the%20United%20States.&text=During%202013%E2%80%932017%2C%20the%20average,geographic%20location%20and%20patient%20population.) | | [FDA (2023)](https://www.accessdata.fda.gov/scripts/cder/daf/index.cfm?event=overview.process&varApplNo=217417) | |
| Hematology/Coagulation | Sickle Cell Disease | Yes | 0 | 100% | 0% | | 52.6 | 52.2 | 0.60 | 30.0 | Yes | [CDC (2023)](https://www.cdc.gov/ncbddd/sicklecell/features/scd-related-death-age.html) | | | [CDC (2023)](https://www.cdc.gov/ncbddd/sicklecell/facts.html#print) | [CDC (2023)](https://www.cdc.gov/ncbddd/sicklecell/facts.html#print) | | [Jiao et al. (2023)](https://ashpublications.org/bloodadvances/article/7/13/3276/494890/Long-term-survival-with-sickle-cell-disease-a) | | [Beaudoin et al. (2023)](https://icer.org/wp-content/uploads/2023/08/ICER_SCD_Final_Report_FOR_PUBLICATION_082123.pdf) | | [Beaudoin et al. (2023)](https://icer.org/wp-content/uploads/2023/08/ICER_SCD_Final_Report_FOR_PUBLICATION_082123.pdf) | [CDC (2023)](https://www.cdc.gov/ncbddd/sicklecell/data.html#:~:text=In%20the%20United%20States&text=It%20is%20estimated%20that%3A,every%2016%2C300%20Hispanic%2DAmerican%20births.) | | [FDA (2023)](https://www.accessdata.fda.gov/scripts/cder/daf/index.cfm?event=overview.process&ApplNo=213137) | |
| Neurology | Friedrich's Ataxia | No | 13 | 75% | 0% | | 36.5 | 23.5 | 0.59 | 2.0 | Yes | [Indelicato et al. (2020)](https://www.ncbi.nlm.nih.gov/pmc/articles/PMC7397644/pdf/13023_2020_Article_1475.pdf) | | | [Williams et al. (2023)](https://www.ncbi.nlm.nih.gov/books/NBK563199/#:~:text=Overall%2C%20the%20prognosis%20of%20FA,most%20frequent%20cause%20of%20death.) | Author's assumption | | [Williams et al. (2023)](https://www.ncbi.nlm.nih.gov/books/NBK563199/#:~:text=Overall%2C%20the%20prognosis%20of%20FA,most%20frequent%20cause%20of%20death.) | | Calculation | | [Buesch et al. (2022)](https://www.valueinhealthjournal.com/article/S1098-3015(21)02917-X/fulltext) | [Williams et al. (2023)](https://www.ncbi.nlm.nih.gov/books/NBK563199/#:~:text=FA%20has%20an%20autosomal%20recessive,people%20of%20Western%20European%20descent.) | | [FDA (2023)](https://www.accessdata.fda.gov/scripts/cder/daf/index.cfm?event=overview.process&varApplNo=216718) | |
| Pulmonary | Mycobacterium Avium Complex (MAC) Lung Disease | Yes | 60 | #REF! | 36% | | 70.0 | 10.0 | 0.58 | 206.6 | Yes | [UTHealth](https://www.maclungdisease.org/frequently-asked-questions/#:~:text=MAC%20pulmonary%20(lung)%20disease',and%20often%2Dexcessive%20alcohol%20consumption.) | | | [Donohue (2021)](https://bmcinfectdis.biomedcentral.com/articles/10.1186/s12879-021-05925-y) | [Kim et al. (2022)](https://www.nature.com/articles/s41598-022-06022-z/tables/1) | | Calculation | | [Marras et al. (2014)](https://pmc.ncbi.nlm.nih.gov/articles/PMC3972981/#:~:text=There%20was%20agreement%20between%20nonexperts,important%20areas%20for%20targeted%20education.) | | [Murata et al. (2023)](https://www.tandfonline.com/doi/full/10.1080/13696998.2023.2189858?rfr_dat=cr_pub++0pubmed&url_ver=Z39.88-2003&rfr_id=ori%3Arid%3Acrossref.org#d1e1721) | [Cleveland Clinic (2021)](https://my.clevelandclinic.org/health/diseases/22256-mac-lung-disease) | | FDA | |
| Infectious Disease (viral) | COVID-19 | No | 38 | 18% | 31% | | 69.6 | 31.6 | 0.58 | 6618.5 | No | [CDC (2020)](https://www.cdc.gov/mmwr/volumes/69/wr/mm6939e1.htm#:~:text=As%20a%20result%2C%20the%20median,age%20shifts%20were%20observed%20nationwide.) | | | [AAP (2023)](https://www.aap.org/en/pages/2019-novel-coronavirus-covid-19-infections/children-and-covid-19-state-level-data-report/) | [Abul et al. (2023)](https://www.ncbi.nlm.nih.gov/pmc/articles/PMC9633621/pdf/main.pdf) | | [ICER et al. (2022)](https://icer.org/wp-content/uploads/2021/08/ICER_COVID_19_Final_Evidence_Report_051022.pdf) | | Calculation | | [ICER et al. (2020)](https://icer.org/wp-content/uploads/2020/10/ICER-COVID_Revised_Report_20200624.pdf) | [KFF (2022)](https://www.kff.org/racial-equity-and-health-policy/issue-brief/covid-19-cases-and-deaths-vaccinations-and-treatments-by-race-ethnicity-as-of-fall-2022/) | | [FDA (2023)](https://www.accessdata.fda.gov/scripts/cder/daf/index.cfm?event=overview.process&varApplNo=217188) | |
| Dermatology | Prurigo Nodularis | No | 53 | 5% | 48% | | 81.4 | 28.4 | 0.58 | 52.9 | No | [Taghados et al (2024)](https://pmc.ncbi.nlm.nih.gov/articles/PMC11015701/) | | | [Ständer et al (2020)](https://pmc.ncbi.nlm.nih.gov/articles/PMC8362303/) | [Ständer et al (2020)](https://pmc.ncbi.nlm.nih.gov/articles/PMC8362303/) | | [Whang et al (2022)](https://www.jaad.org/article/S0190-9622(21)01028-8/abstract) | | Calculation | | [Stander et al (2024)](https://onlinelibrary.wiley.com/doi/10.1111/jdv.19800#:~:text=NRS%20(%C2%B12.37)%20and-,17.49,-for%20DLQI%20(%C2%B17.01) | [Kwatra et al (2025)](https://www.sciencedirect.com/science/article/pii/S2666328724000415#:~:text=It%20was%20found%20that%20PN,in%20the%20Medicare%2Dinsured%20population.) | | [FDA OPD Listing](https://www.accessdata.fda.gov/scripts/opdlisting/oopd/) | |
| Endocrinology/Metabolism/Bone | Osteoporosis | No | 66 | N/A | 27% | | 72.2 | 5.9 | 0.57 | 102.0 | No | [Tomasevic-Todorovic et al. (2018)](https://www.ncbi.nlm.nih.gov/pmc/articles/PMC5985792/) | | | [N/A](https://pdf.sciencedirectassets.com/280305/1-s2.0-S1110116421X00075/1-s2.0-S111011642200059X/main.pdf?X-Amz-Security-Token=IQoJb3JpZ2luX2VjEJr%2F%2F%2F%2F%2F%2F%2F%2F%2F%2FwEaCXVzLWVhc3QtMSJHMEUCIQDfgbAIfFfYqR22nCyHpxUhClswF3qItbJ1nF2123NhpwIge4yEVy1UvOpsuf0YMkjXRV9SMUQrswouzLqkCE0s0LMqswUIYhAFGgwwNTkwMDM1NDY4NjUiDHF6sBvIuMj%2Bm5yv5SqQBbDsi%2B6Mf66nipnOncuJrjdcnMc9qJf70ggMuqLrZw%2BmihSHTw50WdGPgzgR9GNAaNNDj%2FnzY50f2u0LXaUpDUW9TgcmfWGisf03H3H5%2BrZnrwGC8mQGWBGRC92vZ6WuxOtt9Yq6C0x9R1aQR98DLf4XnaPsFQGENitq04TN%2B518eUDVf97pNq4UcfaLfQykA6eEIHyjttQ7NbzSc1Jkf4Y5%2B%2FCHARTTPETVrJtaYIEMNZh3L%2FnSKX9rvXjrrmd05V6HKtbwj5ynA%2Bz%2BzRAPYbcFGclS6FzJxR7oN07CofxrHZ%2FQltyn3Y9OSykYIQ1O7tXh03%2F7fyAS3z8cbiCBuC0P3cfyYMAVF8ttusSDJ9oIkFqGH%2FOLZzEE9Pksp1GGySzJmGl4PiL7oGzwioFB2yc2Ew5K5h65hYyXf1u8if7UyNzvU5%2B8G9aeE3luEcArrX6%2FdkTpdCSUmosv9T2btk9dtDR%2BjFVyGWPr%2BSEM9aDkePxUTYPh92ms4KVsZkp8KTUBvi9YMZsQ5BQFVDGnVF5Wt52aCS6RgcCR1GgdIgXXoPOhcCdMw03F%2Fs33tzvnciWn6uyFWHLkmIK0nkddPTM2ZK7x3QDqMVJSgNuATBTXg%2BX8aw7DwAjhNJhUgXOvmWSFfsTU2TlgajarUn4WDvf8VdD073SMjC6k1WsysKpe8gs8woTxMytASb%2FY4o2e07AtlNM6mDrteG7TxiPE1%2FBU%2BGHD1MKtxVGgXIRud1pV9yscyqpi7ZkpNUKPkzKiN7tT7OdJS9euoebSRqs3i8QVI6IFZ5zLaUa0Q7J05lf%2BgprdQ%2FAsQSX%2Fk8H%2BYT2oKgFvky1GB4brS%2FhkeJOwqe6XwrfLMEh%2BK%2F40WtBY42I8MPPI9K0GOrEBwCocMu62wHvVo5Vv%2BS8zBT%2FPp8J10oGmhDAyQtJpT3O7q%2B%2F%2BqHE4s0FqwLjnZlA%2BKCn4TuB%2FzchAOddeBTfgYIfhsy8Le9g8wN39Azrenry0paFLX6DZwUzbW5nKJYLDm71uAsHdhQcHYYFvWAw8SdRdLoWL1mONW4NZG8SUvkXRZpF0Y9GXyZ3CTi6WqZp25mI7OxfNJWLubQ%2FWUAeyvSKWc2lHe9PMvfV%2BDQ7i7uZC&X-Amz-Algorithm=AWS4-HMAC-SHA256&X-Amz-Date=20240202T182242Z&X-Amz-SignedHeaders=host&X-Amz-Expires=300&X-Amz-Credential=ASIAQ3PHCVTYT4ZZLCJV%2F20240202%2Fus-east-1%2Fs3%2Faws4_request&X-Amz-Signature=bcc5da49f32dbd1f260ad2a267bdbe1dfb60d9e26a1d46bb79cdad48b3062dc2&hash=ecc6dc8023866edb60a261693bbefee4924eca822657d81f09f4951eb4515ed8&host=68042c943591013ac2b2430a89b270f6af2c76d8dfd086a07176afe7c76c2c61&pii=S111011642200059X&tid=spdf-55681be0-6cf4-45ca-af07-c1f7d37068ff&sid=845a4fcf493ff9470c5ab3a421d342b35f74gxrqa&type=client&tsoh=d3d3LnNja) | [Sarafrazi et al. (2021)](https://www.cdc.gov/nchs/data/databriefs/db405-H.pdf) | | [Abarahamsen et al. (2015)](https://pubmed.ncbi.nlm.nih.gov/25663501/) | | Calculation | | [Rizzo et al (2022)](https://pmc.ncbi.nlm.nih.gov/articles/PMC9568431/) | [Sarafrazi et al. (2021)](https://www.cdc.gov/nchs/data/databriefs/db405-H.pdf) | | [FDA (2019)](https://www.accessdata.fda.gov/scripts/opdlisting/oopd/) | |
| Hematology/Coagulation | Hemophilia A | No | 0 | 49% | 4% | | 70.4 | 70.2 | 0.57 | 12.0 | Yes | [Kulkarni et al. (2009)](https://pubmed.ncbi.nlm.nih.gov/19637999/) | | | [CDC (2023)](https://www.cdc.gov/ncbddd/hemophilia/communitycounts/registry-report-males/age.html) | [CDC (2023)](https://www.cdc.gov/ncbddd/hemophilia/communitycounts/registry-report-males/age.html) | | [Hassan et al. (2021)](https://www.ncbi.nlm.nih.gov/pmc/articles/PMC7986360/pdf/JTH-19-645.pdf) | | Calculation | | [ICER et al. (2020)](https://icer.org/wp-content/uploads/2020/10/ICER_Hemophilia-A_Final-Report_112020.pdf) | [Soucie et al. (2020)](https://www.ncbi.nlm.nih.gov/pmc/articles/PMC8117262/) | | [FDA (2023)](https://www.accessdata.fda.gov/scripts/opdlisting/oopd/) | |
| Psychiatry | Postpartum Depression (PPD) | No | 27 | 4% | 0% | | 78.7 | 51.7 | 0.55 | 9700.0 | No | [Schaeffer and Aragao (2023)](https://www.pewresearch.org/short-reads/2023/05/09/facts-about-u-s-mothers/) | | | [Silverman et al. (2017)](https://www.ncbi.nlm.nih.gov/pmc/articles/PMC5462547/) | Assumption | | Assumption | | Calculation | | [Eldar-Lissai et al. (2020)](https://pubmed.ncbi.nlm.nih.gov/32191592/) | [Wilkinson, Anderson, and Wheeler (2017)](https://pubmed.ncbi.nlm.nih.gov/27832444/) | | [FDA (2023)](https://www.accessdata.fda.gov/scripts/opdlisting/oopd/) | |
| Endocrinology/Metabolism/Bone | Alpha-mannosidosis | No | 2.5 | 100% | 0% | | 40.3 | 22.4 | 0.53 | 0.2 | Yes | [Adam et al. (2019)](https://www.ncbi.nlm.nih.gov/pmc/articles/PMC6557729/#:~:text=It%20was%20noted%20that%20initial,enlarged%20head%2C%20spine%20curvature%2C%20hearing) | | | Author's Assumption | Author's Assumption | | [Hennermann et al. (2022)](https://www.ncbi.nlm.nih.gov/pmc/articles/PMC9308362/) | | [Antanaskovića et al. (2023)](https://www.tandfonline.com/doi/full/10.1080/13102818.2023.2271574) | | [Adam et al. (2019)](https://www.ncbi.nlm.nih.gov/pmc/articles/PMC6557729/#:~:text=It%20was%20noted%20that%20initial,enlarged%20head%2C%20spine%20curvature%2C%20hearing) | [NORD (2023)](https://rarediseases.org/rare-diseases/alpha-mannosidosis/#affected) | | [FDA (2023)](https://www.accessdata.fda.gov/scripts/opdlisting/oopd/) | |
| Neurology | Hereditary Transthyretin-Mediated Amyloidosis | No | 68 | 0% | 32% | | 72.8 | 4.7 | 0.53 | 1.0 | Yes | [Gertz (2017)](https://www.ajmc.com/view/hereditary-attr-amyloidosis-burden-of-illness-and-diagnostic-challenges-article) | | | [Guthrie et al. (2019)](https://www.pharllc.com/wp-content/uploads/2019/06/hATTR-Amyloidosis-PNS-Epi-poster.pdf) | [Guthrie et al. (2019)](https://www.pharllc.com/wp-content/uploads/2019/06/hATTR-Amyloidosis-PNS-Epi-poster.pdf) | | [Swiecicki et al. (2015)](https://www.tandfonline.com/doi/abs/10.3109/13506129.2015.1019610) | | Calculation | | [Lasser et al. (2018)](https://icer.org/wp-content/uploads/2020/10/ICER_Amyloidosis_Final_Evidence_Report_101718.pdf) | [Lasser et al. (2018)](https://icer.org/wp-content/uploads/2020/10/ICER_Amyloidosis_Final_Evidence_Report_101718.pdf) | | [FDA (2023)](https://www.accessdata.fda.gov/scripts/opdlisting/oopd/) | |
| Endocrinology/Metabolism/Bone | Acid Sphingomyelinase Deficiency | No | 5.18407 | 89% | 0% | | 21.3 | 16.1 | 0.52 | 0.4 | Yes | [McGovern et al. (2021)](https://www.ncbi.nlm.nih.gov/pmc/articles/PMC8111900/) | | | [Pulikottil-Jacob et al. (2023)](https://www.sciencedirect.com/science/article/pii/S2214426923000861) | Author's Assumption | | [Pulikottil-Jacob et al. (2023)](https://www.ncbi.nlm.nih.gov/pmc/articles/PMC10767269/) | | Calculation | | [Gemeinsamer (2023)](https://www.g-ba.de/downloads/91-1455-882/2023-03-16_Resolution_Olipudase_alfa_D-869_EN.pdf) | [NORD (2022)](https://rarediseases.org/rare-diseases/acid-sphingomyelinase-deficiency/) | | [FDA (2023)](https://www.accessdata.fda.gov/scripts/opdlisting/oopd/) | |
| Neurology | Alzheimer's Disease | Yes | 65 | 0% | 97% | | 70.8 | 5.8 | 0.52 | 2012.0 | No | [NIH (2022)](https://www.nia.nih.gov/health/alzheimers-symptoms-and-diagnosis/what-are-signs-alzheimers-disease#:~:text=For%20most%20people%20with%20Alzheimer's,30s%2C%20although%20this%20is%20rare.) | | | Author's Assumption | [CDC (2019)](https://www.cdc.gov/aging/publications/features/Alz-Greater-Risk.html) | | [NIH (2022)](https://www.nia.nih.gov/health/alzheimers-symptoms-and-diagnosis/what-are-signs-alzheimers-disease#:~:text=For%20most%20people%20with%20Alzheimer's,30s%2C%20although%20this%20is%20rare.) | | [Lin et al. (2023)](https://icer.org/wp-content/uploads/2023/04/ICER_Alzheimers-Disease_Final-Report_For-Publication_04172023.pdf) | | [Lin et al. (2023)](https://icer.org/wp-content/uploads/2023/04/ICER_Alzheimers-Disease_Final-Report_For-Publication_04172023.pdf) | [Alzheimer's Association (2023)](https://www.alz.org/alzheimers-dementia/facts-figures#:~:text=More%20than%206%20million%20Americans%20of%20all%20ages%20have%20Alzheimer's,older%20(10.7%25)%20has%20Alzheimer's.) | | [FDA (2023)](https://www.accessdata.fda.gov/scripts/cder/daf/index.cfm?event=BasicSearch.process) | |
| Hematology/Coagulation | Chronic graft-versus-host disease (cGVHD) | No | 49 | 6% | 29% | | 66.3 | 17.0 | 0.51 | 4.2 | Yes | [DeFelipp et al (2021)](https://ashpublications.org/bloodadvances/article/5/20/4278/476901/Nonrelapse-mortality-among-patients-diagnosed-with) | | | [Baird et al (2011)](https://pmc.ncbi.nlm.nih.gov/articles/PMC2872081/#:~:text=Chronic%20Graft%2DVersus%2DHost%20Disease,life%20and%20decreased%20overall%20survival.) | [Baird et al (2011)](https://pmc.ncbi.nlm.nih.gov/articles/PMC2872081/#:~:text=Chronic%20Graft%2DVersus%2DHost%20Disease,life%20and%20decreased%20overall%20survival.) | | Calculation | | [DeFelipp et al (2021), Takahashi et al (2021)](https://ashpublications.org/bloodadvances/article/5/20/4278/476901/Nonrelapse-mortality-among-patients-diagnosed-with) | | [Kurosawa et al (2017)](https://www.astctjournal.org/article/S1083-8791(17)30520-7/fulltext#t0020) | [Wingard et al (](https://linkinghub.elsevier.com/retrieve/pii/S2666-6367(20)30101-9) | | [FDA OPD Listing](https://www.accessdata.fda.gov/scripts/cder/daf/index.cfm?event=BasicSearch.process) | |
| Neurology | Rett Syndrome | No | 3 | 52% | 4% | | 50.0 | 47.3 | 0.51 | 3.2 | Yes | [Tarquinio et al. (2015)](https://www.ncbi.nlm.nih.gov/pmc/articles/PMC4442062/pdf/nihms664890.pdf) | | | [May et al. (2023)](https://www.ncbi.nlm.nih.gov/pmc/articles/PMC10071755/) | [May et al. (2023)](https://www.ncbi.nlm.nih.gov/pmc/articles/PMC10071755/) | | [Tarquinio et al. (2015)](https://www.rettsyndrome.org/wp-content/uploads/Changing-face-of-survival-in-RS-2015.pdf) | | Calculation | | [Rozensztrauch et al. (2021)](https://www.ncbi.nlm.nih.gov/pmc/articles/PMC8111277/pdf/10.1177_03000605211007714.pdf) | [May et al. (2023)](https://www.ncbi.nlm.nih.gov/pmc/articles/PMC10071755/) | | [FDA (2023)](https://www.accessdata.fda.gov/scripts/cder/daf/index.cfm?event=BasicSearch.process) | |
| Cardiovascular Disease | Pulmonary Arterial Hypertension | No | 50 | 8% | 17% | | 56.3 | 6.2 | 0.51 | 1.5 | Yes | [McGoon et al (2012)](https://pmc.ncbi.nlm.nih.gov/articles/PMC9487467/) | | | [McGoon et al (2012)](https://pmc.ncbi.nlm.nih.gov/articles/PMC9487467/) | [Rothbard et al (2020)](https://pmc.ncbi.nlm.nih.gov/articles/PMC8016023/) | | Calculation | | [Cedars Sinai](https://www.cedars-sinai.org/newsroom/unique-cell-based-approach-for-pulmonary-arterial-hypertension-shown-to-be-safe/) | | [Borgese et al (2021)](https://publications.ersnet.org/content/erj/57/2/2000414) | [Beshay et al (2020)](https://www.sciencedirect.com/science/article/pii/S0954611120302390) | | [FDA OPD Listing](https://www.accessdata.fda.gov/scripts/cder/daf/index.cfm?event=BasicSearch.process) | |
| Neurology | Myasthenia Gravis | No | 48 | 10% | 63% | | 80.7 | 32.7 | 0.51 | 37.0 | Yes | [Fustes, Rodriguez, and Fustes (2020)](https://www.ncbi.nlm.nih.gov/pmc/articles/PMC6991148/#:~:text=Myasthenia%20gravis%20is%20encountered%20at,dominance%20%5B6%2D7%5D.) | | | [CHOP](https://www.chop.edu/conditions-diseases/myasthenia-gravis#:~:text=Myasthenia%20gravis%20is%20rare%3A%20about,%2C%20ethnicities%2C%20and%20both%20genders.) | [Estevez et al. (2023)](https://www.sciencedirect.com/science/article/pii/S217358082200013X) | | [NIH (2023)](https://www.ninds.nih.gov/health-information/disorders/myasthenia-gravis) | | Calculation | | [Tice et al. (2021)](https://icer.org/wp-content/uploads/2021/03/ICER_Myasthenia-Gravis_Final-Report_Unmasked_Data.pdf) | [Rodrigues et al. (2023)](https://onlinelibrary.wiley.com/doi/10.1002/mus.28006) | | [FD A(2023)](https://www.accessdata.fda.gov/scripts/cder/daf/index.cfm?event=overview.process&varApplNo=761195) | |
| Infectious Disease (non viral) | Severe Malaria | No | 37 | 15% | 1% | | 79.5 | 42.5 | 0.49 | 0.0 | Yes | [Park et al. (2023)](https://academic.oup.com/jtm/article/30/3/taad013/7015603?login=false) | | | [Park et al. (2023)](https://academic.oup.com/jtm/article/30/3/taad013/7015603?login=false) | [Park et al. (2023)](https://academic.oup.com/jtm/article/30/3/taad013/7015603?login=false) | | [Walter et al. (2022)](https://jamanetwork.com/journals/jama/fullarticle/2788780) | | Calculation | | [Makatita et al. (2019)](https://www.jcdr.net/ReadXMLFile.aspx?id=13010) | [CDC (2023)](https://www.cdc.gov/malaria/about/index.html#:~:text=The%20risk%20of%20malaria%20in,cases%20of%20malaria%20each%20year.) | | [FDA (2020)](https://www.accessdata.fda.gov/scripts/cder/daf/index.cfm?event=overview.process&varApplNo=213036) | |
| Neurology | Duchenne Muscular Dystrophy (DMD) | Yes, verified | 3 | 65% | 0% | | 22.0 | 15.1 | 0.46 | 6.5 | Yes | [Ciafaloni et al. (2018)](https://www.ncbi.nlm.nih.gov/pmc/articles/PMC5868738/pdf/nihms951659.pdf) | | | [Giegerich et al. (2019)](https://www.valueinhealthjournal.com/article/S1098-3015(19)31332-4/pdf) | Author's assumption | | [Broomfield et al. (2021)](https://www.neurology.org/doi/pdf/10.1212/WNL.0000000000012910) | | ICER et al. (2019) | | ICER et al. (2019) | [Giegerich et al. (2022)](https://www.ispor.org/docs/default-source/euro2019/giegerich-eispor-eu-2019-poster26october2019-pdf.pdf?sfvrsn=93517960_0) | | [FDA (2023)](https://www.fda.gov/media/169715/download?attachment) | |
| Renal Disease | Hyperoxaluria Type 1 | No | 6 | 41% | 39% | | 47.2 | 24.3 | 0.45 | 0.3 | Yes | [Soliman et al. (2017)](https://www.ncbi.nlm.nih.gov/pmc/articles/PMC5921832/) | | | [Wang et al. (2021)](https://www.ncbi.nlm.nih.gov/pmc/articles/PMC8488346/) | [Mucha et al. (2022)](https://www.jmcp.org/doi/epdf/10.18553/jmcp.2022.28.3.316) | | [Metry et al. (2023)](https://www.kireports.org/article/S2468-0249(23)01404-3/fulltext) | | [CADH (2023)](https://www.ncbi.nlm.nih.gov/books/NBK596631/pdf/Bookshelf_NBK596631.pdf) | | [Hayley et al. (2023)](https://www.tandfonline.com/doi/epdf/10.1080/13696998.2023.2176678?needAccess=true) | [Shah et al. (2022)](https://www.ncbi.nlm.nih.gov/books/NBK558987/) | | [FDA (2023)](https://www.accessdata.fda.gov/scripts/cder/daf/index.cfm?event=overview.process&varApplNo=215842) | |
| Hematology/Coagulation | Niemann-Pick Disease | No | 4 | 43% | 7% | | 22.3 | 17.9 | 0.44 | 0.1 | Yes | [Baxter et al (2022)](https://www.nature.com/articles/s41598-022-06112-y/tables/1) | | | [Burton et al (2021)](https://www.sciencedirect.com/science/article/pii/S1096719221007411#:~:text=Background-,Niemann%2DPick%20Disease%20Type%20C%20(NPC)%20is%20an%20ultra,and%2025%20years%20of%20age.) | [Burton et al (2021)](https://www.sciencedirect.com/science/article/pii/S1096719221007411#:~:text=Background-,Niemann%2DPick%20Disease%20Type%20C%20(NPC)%20is%20an%20ultra,and%2025%20years%20of%20age.) | | [Burton et al (2021)](https://www.sciencedirect.com/science/article/pii/S1096719221007411#:~:text=Background-,Niemann%2DPick%20Disease%20Type%20C%20(NPC)%20is%20an%20ultra,and%2025%20years%20of%20age.) | | Calculation | | [Mengel et al (2021)](https://ojrd.biomedcentral.com/articles/10.1186/s13023-021-02105-8?) | [Burton et al (2021)](https://pubmed.ncbi.nlm.nih.gov/34304992/#:~:text=NPC%20prevalence%20of%200.95%20per%20million%20people) | | [FDA OPD Listing](https://www.accessdata.fda.gov/scripts/opdlisting/oopd/) | |
| Ophthalmology | Thyroid Eye Disease | No | 43 | 5% | 10% | | 80.1 | 37.1 | 0.44 | 2.9 | Yes | [Cleveland Clinic (2022)](https://my.clevelandclinic.org/health/diseases/17558-thyroid-eye-disease) | | | [Bartley et al. (1994)](https://www.ncbi.nlm.nih.gov/pmc/articles/PMC1298522/pdf/taos00007-0494.pdf) | [Medghalchi et al. (2018)](https://www.ncbi.nlm.nih.gov/pmc/articles/PMC6276727/) | | [Yale Medicine (2023)](https://www.yalemedicine.org/conditions/graves-disease) | | Calculation | | [Smith et al. (2023)](https://jamanetwork.com/journals/jamaophthalmology/fullarticle/2800106) | [NORD (2020)](https://rarediseases.org/rare-diseases/thyroid-eye-disease/) | | [FDA (2020)](https://www.accessdata.fda.gov/scripts/cder/daf/index.cfm?event=overview.process&varApplNo=211723) | |
| Endocrinology/Metabolism/Bone | Pompe Disease | No | 16 | 58% | 4% | | 42.8 | 25.1 | 0.42 | 3.6 | Yes | [Lewin Group (2023) and Cleveland Clinic 2023)](https://everylifefoundation.org/wp-content/uploads/2023/09/EveryLife-Cost-of-Delayed-Diagnosis-in-Rare-Disease_Final-Full-Study-Report_0914223.pdf) | | | [Hagemans et al. (2005)](https://oup.silverchair-cdn.com/oup/backfile/Content_public/Journal/brain/128/3/10.1093/brain/awh384/2/awh384.pdf?Expires=1709929997&Signature=MbKtXwDcSSH5V618VCBldBcYeqbyu60C-URoQGnrgoU4YPMNaLHEh7iGwkhJH1TuNNkXHdDLxSvrV3~8mAlcgBPnnpzzSKKsRM9a5Ha2rR2FpDG9wgSZ3eIsR1mjRR3GGVmiqLD7CL8Y38qz4mBhgTgY-81RUVx8mITz103es82yjvkemkn3WASIeW1sy5hV29hsJ3NEh4hZlHMmKCs6~VCbCh3DspKG112VFNYiiNntsgIcZQm4JN4o602d3yq5ybTmKXWs0deEmju~1U3SBlbJnXCm6ga4SLavEEdQVUFHFYMukavQWmBw~RMRId3a7oOiQDC8nHfc-yFq~QIPYQ__&Key-Pair-Id=APKAIE5G5CRDK6RD3PGA) | [Gungor et al. (2011)](https://www.ncbi.nlm.nih.gov/pmc/articles/PMC3135500/) | | [Gungor et al. (2011)](https://www.sciencedirect.com/science/article/abs/pii/S0149291811003067) | | [Kanters et al. (2017)](https://ojrd.biomedcentral.com/articles/10.1186/s13023-017-0731-0) | | [Kanters et al. (2017)](https://ojrd.biomedcentral.com/articles/10.1186/s13023-017-0731-0) | [Dasouki et al. (2014)](https://www.ncbi.nlm.nih.gov/pmc/articles/PMC4311397/) | | [FDA (2023)](https://www.accessdata.fda.gov/scripts/cder/daf/index.cfm?event=overview.process&varApplNo=761204) | |
| Neurology | Parkinson's Disease (PD) | No | 69 | N/A | 83% | | 73.8 | 3.8 | 0.39 | 9.3 | No | [Keener et al. (2018)](https://www.ncbi.nlm.nih.gov/pmc/articles/PMC6924612/pdf/nihms-1025523.pdf) | | | N/A | [Yang et al. (2020)](https://www.ncbi.nlm.nih.gov/pmc/articles/PMC7347582/) | | [Ishihara et al. (2007)](https://www.ncbi.nlm.nih.gov/pmc/articles/PMC2095626/pdf/1304.pdf) | | [Pietzsch et al. (2016)](https://www.neuromodulationjournal.org/article/S1094-7159(21)06030-X/fulltext) | | [Pietzsch et al. (2016)](https://www.neuromodulationjournal.org/article/S1094-7159(21)06030-X/fulltext) | [Marras et al. (2018)](https://www.nature.com/articles/s41531-018-0058-0) | | [FDA (2020)](https://www.accessdata.fda.gov/scripts/cder/daf/index.cfm?event=BasicSearch.process) | |
| Neurology | Spinal Muscular Atrophy | No | 0 | 65% | 0% | | 18.5 | 16.6 | 0.35 | 10.0 | Yes | [Sproule et al. (2012)](https://journals.sagepub.com/doi/10.1177/0883073811415680) | | | [Belter et al. (2018)](https://www.ncbi.nlm.nih.gov/pmc/articles/PMC6004903/) | [Belter et al. (2018)](https://www.ncbi.nlm.nih.gov/pmc/articles/PMC6004903/) | | [Ellis et al. (2019)](https://icer.org/wp-content/uploads/2020/10/ICER_SMA_Final_Evidence_Report_110220.pdf) | | [Ellis et al. (2019)](https://icer.org/wp-content/uploads/2020/10/ICER_SMA_Final_Evidence_Report_110220.pdf) | | [Ellis et al. (2019)](https://icer.org/wp-content/uploads/2020/10/ICER_SMA_Final_Evidence_Report_110220.pdf) | [CDC (2023)](https://www.cdc.gov/nceh/dls/nsmbb_sma.html#:~:text=Spinal%20Muscular%20Atrophy%20(SMA)%20is,out%20of%20every%2010%2C000%20people.) | | [FDA (2023)](https://www.accessdata.fda.gov/scripts/cder/daf/index.cfm?event=BasicSearch.process) | |
| Neurology | Amyotrophic Lateral Sclerosis (ALS) | Yes | 59 | 4% | 53% | | 62.5 | 2.6 | 0.34 | 7.7 | Yes | [Talbott, Malek, & Lacomis (2016)](https://www.sciencedirect.com/science/article/abs/pii/B9780128029732000136) | | | [GARD (2024)](https://rarediseases.info.nih.gov/diseases/11901/juvenile-amyotrophic-lateral-sclerosis) | [Yamakawa et al. (2022)](https://www.ncbi.nlm.nih.gov/pmc/articles/PMC8849587/) | | [Talbott, Malek, & Lacomis (2016)](https://www.sciencedirect.com/science/article/abs/pii/B9780128029732000136) | | [Makam et al. (2022)](https://icer.org/wp-content/uploads/2022/02/ICER_ALS-Final-Evidence-Report_12MonthUpdate.pdf) | | [Makam et al. (2022)](https://icer.org/wp-content/uploads/2022/02/ICER_ALS-Final-Evidence-Report_12MonthUpdate.pdf) | [Mehta et al. (2023)](https://pubmed.ncbi.nlm.nih.gov/35422180/) | | [FDA (2022)](https://www.accessdata.fda.gov/scripts/cder/daf/index.cfm?event=BasicSearch.process) | |
| Hematology/Coagulation | Primary Hemophagocytic Lymphohistiocytosis (pHLH) | No | 0 | 88% | 0% | | 5.5 | 5.0 | 0.32 | 2.0 | Yes | [Bohm et al. (2024)](https://ashpublications.org/blood/article/143/10/872/498853/Survival-in-primary-hemophagocytic) | | | [Abbasi et al. (2023)](https://pmc.ncbi.nlm.nih.gov/articles/PMC10615402/#:~:text=The%20overall%20survival%20in%20primary,was%2015%20%C2%B1%204.8%20months.) | [Abbasi et al. (2023)](https://pmc.ncbi.nlm.nih.gov/articles/PMC10615402/#:~:text=The%20overall%20survival%20in%20primary,was%2015%20%C2%B1%204.8%20months.) | | Calculation | | [Bohm et al. (2024)](https://ashpublications.org/blood/article/143/10/872/498853/Survival-in-primary-hemophagocytic) | | [Nafees, Lloyd, and Dewilde (2021)](https://jpro.springeropen.com/articles/10.1186/s41687-020-00276-9/tables/3) | [CHOP](https://www.chop.edu/conditions-diseases/hemophagocytic-lymphohistiocytosis-hlh) | | [FDA](https://www.accessdata.fda.gov/scripts/cder/daf/index.cfm?event=BasicSearch.process) | |
| Endocrinology/Metabolism/Bone | Aromatic L-amino acid decarboxylase (AADC) deficiency | Yes | 0 | 100% | 0% | | 15.7 | 15.5 | 0.25 | 0.3 | Yes | [Orphanet (2020)](https://www.orpha.net/en/disease/detail/35708) | | | [Williams et al. (2021)](https://www.tandfonline.com/doi/full/10.1080/03007995.2021.1932449#d1e294) | [Williams et al. (2021)](https://www.tandfonline.com/doi/full/10.1080/03007995.2021.1932449#d1e294) | | Calculation | | [Simons et al. (2023)](https://pmc.ncbi.nlm.nih.gov/articles/PMC10611606/#Sec12) | | [Monteleone et al. (2025)](https://hqlo.biomedcentral.com/articles/10.1186/s12955-025-02335-5#Sec8) | [DiBacco et al. (2023)](https://onlinelibrary.wiley.com/doi/full/10.1002/cns3.20010#:~:text=Recent%20calculations%20indicate%20a%20birth,number%20of%20currently%20diagnosed%20patients.&text=Metabolic%20pathways%20of%20biogenic%20monoamine%20neurotransmitters.) | | FDA | |
| Neurology | Multiple Sclerosis (MS) | No | 25 | 3% | 2% | | 69.3 | 44.3 | 0.19 | 309.2 | No | [McGInley et al. (2021)](https://watermark.silverchair.com/jama_mcginley_2021_rv_200019_1622223670.83305.pdf?token=AQECAHi208BE49Ooan9kkhW_Ercy7Dm3ZL_9Cf3qfKAc485ysgAAA2owggNmBgkqhkiG9w0BBwagggNXMIIDUwIBADCCA0wGCSqGSIb3DQEHATAeBglghkgBZQMEAS4wEQQMv_jVcTxr864vZEkiAgEQgIIDHeRkLrfsh4vDfmaO4MDSe-c3ZybDfzEY_0QyWKynm44yN2cMf2Ne8coT-H7P3uvQSu_jN1soq-WgnAUXHkNjrFWy13EuTDBfXqXGmEGyUhLuE30Tdx2hJZlbUrlaal9NGI6ixRHukGcKDDWUTn8Z7FLV4dVEgwt_HgJ2fMGFuBUkB56evZnN73WUb6pHf2Ak4LKfG9upGYwK-nmZbgcEqc4HF40RfnvhTRb3sgRr0P_8ux-0cJW_Mjwhy2S_7PdCIFF-yxiiK5soRKFoBbJ1LEgQHE9y1yK2LabD2olaKnQswv4xdFaCGPxTsWo79K7taffnQpm6E-HuzJaxXGtc74sbW9qPbsAQUKoaRgUIwEyZaHFNwfu2cTEPL7orW4k7a2A3qRuNaKWy8CnCZCGVPQyhmqGpXd3v-6Gy4Tx78WdiDvDl66R9V726jNVWEAOLbR2XJdVrdiZ_Yot58fI5zporP4VJUugXAHliyUp0ieq85etz26L4nHit_Yy9PONL28sl7jzFnHfoecjAS8sKpeMySyFP0ySAPsG81JEOv4Y44Z0K6u205Wh09xTQlGgt7uyIw1XCtXr3AwG54r99qUMCK3xRR_ZzL8yOGpdipME6o-qXQHgU8LMoVGuxR6bJ3cV4LsgI9KPbZZC2V8pYPE8n6u5-_5R2P8M3vvHtg4z8-0yI77AkS0ongOVt4QCyA0JzhMP8xHYrGr5ygcWqyoQxqhnrGpqVxMg2L0PoeSOP5Ybh6fxPaFFtVGU9CcWPhWbkbeoMP9-oMdtEVv1EhcS-7jYK0ZmcdJnef5qWGwhy5hglV7fwbb3-v1Bn7qiXXBSxuUPKgfHggvFTkWiFMb65uLd00KdiWi0rydsEb5izw8iRQAFGGseFaAx_r3tGbRl9hDnIIxnMwSXzxDLxf9yF0lj20MR0PMzlYYADSKKKrph38bwaeBVuf-IJYzjRL9m8FjJmRTq9IVjPnCk2c2T2J6xIrmrERkaBGvDn5fdQcRmvYiV0YZK_Vf9BqNxyNaBuG30tWGUgEwOnP43BB2YxRK7j2GGoQYAtQDMZ) | | | [Chitnis et al. (2009)](https://journals.sagepub.com/doi/abs/10.1177/1352458508101933) | [Dilokthornsakul et al. (2016)](https://www.ncbi.nlm.nih.gov/pmc/articles/PMC4799713/pdf/NEUROLOGY2015682146.pdf) | | [Lunde et al. (2017)](https://jnnp.bmj.com/content/jnnp/88/8/621.full.pdf) | | Calculation | | [ICER et al. (2019)](https://icer.org/wp-content/uploads/2020/10/ICER_MS_Final_Evidence_Report_062019.pdf) | [Walin et al. (2019)](https://www.neurology.org/doi/10.1212/wnl.0000000000007035) | | [FDA (2022)](https://www.accessdata.fda.gov/scripts/cder/daf/index.cfm?event=overview.process&varApplNo=761238) | |

S7: Productivity loss among AA and non-AA diseases

| **Time Use Activity** | **AA** | **Matched General Population** | **Hours of Productivity Loss Per Year** | **Non-AA** | **Matched General Population** | **Hours of Productivity Loss Per Year** |
| --- | --- | --- | --- | --- | --- | --- |
| Caring for Household Members/Services | 987 | 1,071 | 84 | 1,103 | 1,181 | 79 |
| Caring for Non-Household Members | 98 | 95 | -3 | 107 | 105 | -3 |
| Education | 172 | 479 | 307 | 47 | 67 | 21 |
| Labor | 867 | 1,196 | 329 | 1,154 | 1,526 | 373 |
| Medical Care | 45 | 21 | -24 | 39 | 22 | -17 |
| Volunteer Activity | 78 | 117 | 39 | 91 | 123 | 32 |
| **Total** | **2,246** | **2,979** | **732** | **2,540** | **3,025** | **485** |
| **Cost** | **$54,734** | **$72,579** | **$17,845** | **$61,892** | **$73,706** | **$11,814** |
| **Difference** |  |  | **$6,031** |  |  |  |

S8: Jiao-Basu productivity estimates by disease

| **High Level Disease** | **AA** | **Difference (Productivity Loss)** | | | | | |  |
| --- | --- | --- | --- | --- | --- | --- | --- | --- |
|  |  | **Caring for Household Members/Services** | **Caring for Non-Household Members** | **Education** | **Labor** | **Medical Care** | **Volunteer Activity** | **TOTAL** |
| Achondroplasia | Yes | 53 | -2 | 70 | 384 | -8 | 26 | **523** |
| Acid Sphingomyelinase Deficiency | No |  |  |  |  |  |  |  |
| Acquired thrombotic thrombocytopenic purpura (aTTP) | No |  |  |  |  |  |  |  |
| Actinic Keratosis | No |  |  |  |  |  |  |  |
| Activated phosphoinositide 3-kinase delta syndrome (APDS) | No |  |  |  |  |  |  |  |
| Active anti-neutrophil cytoplasmic autoantibody-associated vasculitis (Granulomatosis with Polyangiitis) | No |  |  |  |  |  |  |  |
| Active anti-neutrophil cytoplasmic autoantibody-associated vasculitis (Microscopic Polyangiitis) | No |  |  |  |  |  |  |  |
| Acute Hepatic Porphyria (AHP) | No |  |  |  |  |  |  |  |
| Alagille Syndrome | No |  |  |  |  |  |  |  |
| Alpha-mannosidosis | No |  |  |  |  |  |  |  |
| Alzheimer's disease | Yes | 105 | -6 | 16 | 305 | -48 | 79 | **451** |
| Amyotrophic Lateral Sclerosis (ALS) | Yes | 177 | -10 | 26 | 725 | -89 | 94 | **923** |
| Atopic Dermatitis | No |  |  |  |  |  |  |  |
| Attention Deficit Hyperactivity Disorder (ADHD) -- adults | No | 71 | -2 | 41 | 446 | -6 | 23 | **574** |
| Attention Deficit Hyperactivity Disorder (ADHD) -- children/adolescents | No | 60 | -1 | 16 | 354 | -4 | 16 | **442** |
| Beta Thalassemia | No |  |  |  |  |  |  |  |
| Bipolar I Disorder | No | 102 | -3 | 29 | 627 | -12 | 36 | **780** |
| CD55-deficient protein-losing enteropathy (PLE) | No |  |  |  |  |  |  |  |
| COVID-19 | No | 155 | -5 | 27 | 797 | -28 | 61 | **1007** |
| Candidemia/Invasive Candidiasis | No | 143 | -4 | 17 | 506 | -31 | 65 | **697** |
| Chagas disease | Yes | 127 | -4 | 21 | 642 | -19 | 50 | **817** |
| Chronic Idiopathic Constipation (CIC) | No | 11 | 1 | -6 | 2 | 2 | -10 | **-1** |
| Cold Agglutinin Disease | No |  |  |  |  |  |  |  |
| Community-acquired bacterial pneumonia (CABP) | No | 102 | -3 | 18 | 514 | -17 | 46 | **659** |
| Complicated Urinary Tract Infections (cUTI) | No | 31 | 1 | -4 | 67 | 0 | -2 | **92** |
| Complicated intra-abdominal infections (cIAI) | No |  |  |  |  |  |  |  |
| Cushing's Disease | No |  |  |  |  |  |  |  |
| Cystic Fibrosis | No |  |  |  |  |  |  |  |
| Demodex Blepharitis | No |  |  |  |  |  |  |  |
| Diabetic Macular Edema | No | 44 | -1 | 5 | 238 | -3 | 13 | **296** |
| Dry Eye Disease | No | 21 | 0 | 0 | 40 | -1 | 5 | **64** |
| Duchenne Muscular Dystrophy (DMD) | Yes | 107 | -5 | 1272 | 302 | -35 | 52 | **1693** |
| Fabry Disease | Yes | 44 | -1 | 19 | 279 | -4 | 15 | **352** |
| Fascioliasis | No |  |  |  |  |  |  |  |
| Friedrich's Ataxia | No |  |  |  |  |  |  |  |
| Growth Hormone Deficiency | No |  |  |  |  |  |  |  |
| HIV-1 infection | No |  |  |  |  |  |  |  |
| Heart Failure | No | 64 | -3 | 10 | 140 | -23 | 54 | **242** |
| Helicobacter Pylori Infection | No |  |  |  |  |  |  |  |
| Hemophilia A | No |  |  |  |  |  |  |  |
| Hemophilia B | No |  |  |  |  |  |  |  |
| Hereditary Transthyretin-Mediated Amyloidosis | No |  |  |  |  |  |  |  |
| Hereditary angioedema (HAE) | No |  |  |  |  |  |  |  |
| Hospital-Acquired Bacterial Pneumonia | No | 99 | -3 | 13 | 377 | -20 | 50 | **518** |
| Human African trypanosomiasis (HAT) | No |  |  |  |  |  |  |  |
| Hutchinson-Gilford Progeria Syndrome and premature aging | No |  |  |  |  |  |  |  |
| Hypercholesterolemia | No | 19 | 1 | -7 | 50 | 1 | -5 | **58** |
| Hyperoxaluria type 1 | No |  |  |  |  |  |  |  |
| Hypoactive sexual desire disorder (HSDD) | No |  |  |  |  |  |  |  |
| Insomnia | No | 63 | -1 | 9 | 345 | -5 | 21 | **431** |
| Iron deficiency anemia | No | 24 | 2 | -24 | -17 | 5 | -27 | **-37** |
| Irritable bowel syndrome with constipation (IBS-C) | No | 51 | 0 | -5 | 231 | -1 | 6 | **281** |
| Long-chain fatty acid oxidation disorders (LC-FAOD) | No |  |  |  |  |  |  |  |
| Lupus nephritis (LN) | No | 101 | -4 | 34 | 652 | -15 | 42 | **811** |
| Major Depressive Disorder (MDD) | No | 131 | -5 | 32 | 776 | -22 | 53 | **965** |
| Migraine | No | 36 | 2 | -38 | 21 | 4 | -24 | **1** |
| Multiple Sclerosis (MS) | No | 233 | -14 | 58 | 1388 | -120 | 89 | **1635** |
| Myasthenia Gravis | No |  |  |  |  |  |  |  |
| Narcolepsy | No | 35 | 1 | -11 | 135 | 0 | -1 | **158** |
| Neurofibromatosis type 1 (NF1) | No |  |  |  |  |  |  |  |
| Neuromyelitis Optica Spectrum Disorder | No |  |  |  |  |  |  |  |
| Neutropenia | No |  |  |  |  |  |  |  |
| Obstructive Hypertrophic Cardiomyopathy | No |  |  |  |  |  |  |  |
| Obstructive Sleep Apnea (OSA) | No | 87 | -3 | 22 | 526 | -12 | 36 | **657** |
| Osteoporosis | No | 94 | -5 | 14 | 246 | -39 | 73 | **383** |
| Overactive bladder (OAB) | No | 24 | 0 | 0 | 57 | -1 | 5 | **84** |
| Parkinson's disease (PD) | No | 147 | -8 | 18 | 292 | -95 | 104 | **457** |
| Paroxysmal Nocturnal Hemoglobinuria (PNH) | No |  |  |  |  |  |  |  |
| Partial onset seizures (epilepsy) | No | 58 | -2 | 10 | 310 | -7 | 25 | **394** |
| Plaque psoriasis | No | 101 | -3 | 25 | 601 | -15 | 43 | **750** |
| Pompe disease | No |  |  |  |  |  |  |  |
| Post-transplant cytomegalovirus (CMV) | No |  |  |  |  |  |  |  |
| Postpartum Depression (PPD) | No |  |  |  |  |  |  |  |
| Pustular Psoriasis | No | 107 | -4 | 17 | 490 | -21 | 53 | **642** |
| Rett Syndrome | No | 91 | -6 | 255 | 596 | -25 | 49 | **960** |
| Rheumatoid arthritis | No |  |  |  |  |  |  |  |
| Schizophrenia | No | 48 | -2 | 19 | 328 | -7 | 24 | **410** |
| Severe Hypoglycemia | No |  |  |  |  |  |  |  |
| Severe Malaria | No |  |  |  |  |  |  |  |
| Severe systemic lupus erythematosus (SLE) | No | 102 | -4 | 26 | 620 | -16 | 44 | **773** |
| Sickle Cell Disease | Yes | 114 | -4 | 222 | 647 | -18 | 43 | **1004** |
| Spinal muscular atrophy | No |  |  |  |  |  |  |  |
| Tenosynovial giant cell tumor (TGCT) | No | 108 | -3 | 15 | 503 | -15 | 44 | **651** |
| Thermal Burns | No |  |  |  |  |  |  |  |
| Thyroid eye disease | No | 161 | -8 | 23 | 633 | -61 | 85 | **834** |
| Type 1 Diabetes | No |  |  |  |  |  |  |  |
| Ulcerative Colitis | No |  |  |  |  |  |  |  |
| Wet Age-Related Macular Degeneration (AMD) | No | 60 | -3 | 11 | 232 | -17 | 45 | **328** |
| Niemann-Pick Disease | No |  |  |  |  |  |  |  |
| WHIM Syndrome | No |  |  |  |  |  |  |  |
| Supraventricular Tachycardia | No |  |  |  |  |  |  |  |
| Pulmonary Arterial Hypertension | No |  |  |  |  |  |  |  |
| Prurigo Nodularis | No |  |  |  |  |  |  |  |
| Primary Biliary Cholangitis (PBC) | Yes | 80 | -2 | 12 | 402 | -9 | 31 | **514** |
| Familial Chylomicronemia Syndrome (FCS) | No | 39 | -1 | 91 | 202 | -4 | 14 | **341** |
| Transthyretin Mediated Amyloidosis - Cardiomyopathy (ATTR-CM) | No |  |  |  |  |  |  |  |
| Alopecia Areata (AA) | No |  |  |  |  |  |  |  |
| Noncirrhotic Non-Alcoholic Steatohepatitis (NASH) | Yes | 38 | 1 | -5 | 76 | 0 | -2 | **108** |
| Hypertension | No | 51 | -1 | 5 | 216 | -6 | 21 | **286** |
| Classic Congenital Adrenal Hyperplasia (CAH) | No |  |  |  |  |  |  |  |
| Chronic Obstructive Pulmonary Disease (COPD) | No | 13 | 0 | -1 | 23 | 0 | 0 | **35** |
| Molluscum Contagiosum | No |  |  |  |  |  |  |  |
| Uncomplicated Urinary Tract Infections (uUTI) | No | 104 | -4 | 25 | 607 | -19 | 49 | **762** |
| Chronic graft-versus-host disease (cGVHD) | No |  |  |  |  |  |  |  |
| Hypoparathyroidism | No |  |  |  |  |  |  |  |
| Methemoglobinemia | Yes | 92 | -3 | 18 | 479 | -17 | 45 | **613** |
| Aromatic L-amino acid decarboxylase (AADC) deficiency | Yes | 101 | -7 | 2242 | 205 | -77 | 61 | **2526** |
| Mycobacterium Avium Complex (MAC) Lung Disease | Yes | 109 | -5 | 16 | 367 | -33 | 67 | **521** |
| Childhood Cerebral adrenoleukodystrophy (CCALD) | Yes | 59 | -2 | 1006 | 145 | -13 | 32 | **1227** |
| Primary Immunoglobulin A nephropathy (IgAN) | Yes | 20 | 1 | -19 | 36 | 2 | -9 | **31** |
| Uncontrolled Bleeding | Yes | 34 | 0 | -1 | 44 | -3 | 9 | **84** |
| Primary Hemophagocytic Lymphohistiocytosis (pHLH) | No |  |  |  |  |  |  |  |
| Travelers' Diarrhea | No |  |  |  |  |  |  |  |
| Lambert-Eaton Myasthenic Syndrome (LEMS) | No |  |  |  |  |  |  |  |
| Primary Axillary Hyperhidrosis | No |  |  |  |  |  |  |  |
| Light Chain Amyloidosis | Yes | 82 | -1 | 6 | 224 | -11 | 33 | **332** |

S9: Sensitivity analysis for Jiao-Basu productivity estimates

| **Sensitivity Analysis** | **Output** | **AA** | **Matched General Population** | **Hours of Productivity Loss Per Year** | **Non-AA** | **Matched General Population** | **Hours of Productivity Loss Per Year** |
| --- | --- | --- | --- | --- | --- | --- | --- |
| **“Top 1”** | Hours | 2246 | 2979 | 732 | 2540 | 3025 | 485 |
|  | Cost | $54,734 | $72,579 | $17,845 | $61,892 | $73,706 | $11,814 |
|  | Difference |  |  | **$6,031** |  |  |  |
| **“Top 3”** | Hours | 2238 | 2979 | 741 | 2540 | 3025 | 485 |
|  | Cost | $54,522 | $72,579 | $18,058 | $61,892 | $73,706 | $11,814 |
|  | Difference |  |  | **$6,244** |  |  |  |
| **“Top 1” Sensitivity** | Hours | 2004 | 2979 | 975 | 2540 | 3025 | 485 |
|  | Cost | $48,833 | $72,579 | $23,746 | $61,892 | $73,706 | $11,814 |
|  | Difference |  |  | **$11,932** |  |  |  |
| **“Top 3” Sensitivity** | Hours | 1984 | 2979 | 995 | 2540 | 3025 | 485 |
|  | Cost | $48,340 | $72,579 | $24,239 | $61,892 | $73,706 | $11,814 |
|  | Difference |  |  | **$12,426** |  |  |  |

S10: Economic burden of AA and non-AA diseases scenarios (AA = 16)

S11: Sensitivity analysis for economic burden (AA, N=18)

| **Scenario** | **Category** | **Average Direct Non-Medical Costs** | **Productivity Costs** | **Caregiver Burden** | **Average Direct Medical Costs (Excl Rx)** |
| --- | --- | --- | --- | --- | --- |
| **Original (AA=18)** | AAs | $16,184 | $21,852 | $20,268 | $53,447 |
|  | Non-AAs (100) | $8,342 | $9,620 | $8,294 | $29,729 |
|  | Difference | $7,842 | $12,232 | $11,974 | $23,718 |
|  | % Difference | 94.00% | 127.20% | 144.40% | 79.80% |
| **Conservative**  **(AA=18)** | AAs | $2,856 | $15,425 | $9,538 | $53,447 |
|  | Non-AAs (100) | $1,422 | $4,810 | $2,073 | $29,729 |
|  | Difference | $1,434 | $10,615 | $7,464 | $23,718 |
|  | % Difference | 100.90% | 220.70% | 360.00% | 79.80% |
| **Less Conservative**  **(AA=18)** | AAs | $6,088 | $19,106 | $15,031 | $53,447 |
|  | Non-AAs (100) | $4,006 | $10,655 | $6,818 | $29,729 |
|  | Difference | $2,082 | $8,451 | $8,213 | $23,718 |
|  | % Difference | 52.00% | 79.30% | 120.50% | 79.80% |

S12: Sensitivity Analysis: Correlation test for disease costs and disease characteristics (AA, N=18)

|  |  | **AA=18** | |
| --- | --- | --- | --- |
| **Characteristic** | **Category** | **Mean** | **P Value (U Test)** |
| **Total Societal Costs** | AA | $95,684 | 0.003 |
|  | Non-AA | $65,468 | 0.003 |
| **Productivity Costs** | AA | $21,852 | 0.063 |
|  | Non-AA | $9,620 | 0.063 |
| **Caregiver Burden** | AA | $20,268 | 0.092 |
|  | Non-AA | $8,294 | 0.092 |
| **Age of Onset** | AA | 32.1 | 0.895 |
|  | Non-AA | 31.5 | 0.895 |
| **% Under 18** | AA | 29.40% | 0.851 |
|  | Non-AA | 24.20% | 0.851 |
| **% Over 65** | AA | 27.50% | 0.606 |
|  | Non-AA | 23.10% | 0.606 |
| **Life Expectancy** | AA | 59.1 | 0.205 |
|  | Non-AA | 65.1 | 0.205 |
| **Life Years After Diagnosis** | AA | 25.9 | 0.299 |
|  | Non-AA | 31 | 0.299 |
| **QoL** | AA | 0.65 | 0.437 |
|  | Non-AA | 0.69 | 0.437 |
| **Prevalence** | AA | 277 | 0.17 |
|  | Non-AA | 2,250 | 0.17 |

S13: Sensitivity Analysis for Economic Burden (AA, N=16 vs N=18)

| **Scenario** | **Category** | **Average Societal Costs** | **Average Direct Medical Costs** | **Average Direct Non-Medical Costs** | **Productivity Costs** | **Caregiver Burden** | **Average Direct Medical Costs (Excl Rx)** |  |
| --- | --- | --- | --- | --- | --- | --- | --- | --- |
| **AA = 16** | AAs | $91,857 | $64,878 | $16,184 | $17,635 | $20,268 | $51,250 | |
|  | Non-AAs | $65,468 | $57,163 | $8,342 | $9,620 | $8,294 | $29,729 | |
|  | Difference | $26,389 | $7,715 | $7,842 | $8,015 | $11,974 | $21,521 | |
|  | % Difference | 40.30% | 13.50% | 94.00% | 83.30% | 144.40% | 72.40% | |
| **AA = 18** | AAs | $95,684 | $67,865 | $16,184 | $21,852 | $20,268 | $53,447 | |
|  | Non-AAs | $65,468 | $57,163 | $8,342 | $9,620 | $8,294 | $29,729 | |
|  | Difference | $30,216 | $10,702 | $7,842 | $12,232 | $11,974 | $23,718 | |
|  | % Difference | 46.20% | 18.70% | 94.00% | 127.20% | 144.40% | 79.80% | |

*S14:* *Identification of non-AA comparator diseases*

The non-AA comparator group was identified using a stepwise disease-level approach. First, eligible non-oncology FDA drug approvals during the study period (January 2015 to February 2025) were identified. Second, each approval was mapped to the corresponding disease based on the drug’s first FDA-approved indication. Because multiple therapies may be approved for the same disease, multiple approval records could map to the same disease. Third, diseases were classified according to whether they had any therapy approved through the AA pathway during the study period. Diseases with at least one AA-approved therapy were classified as AA-treated diseases, even if they also had therapies approved through the traditional FDA pathway. Diseases with no AA-approved therapies during the study period were classified as non-AA diseases. Fourth, eligible non-AA approvals were reviewed in reverse chronological order, and unique non-AA diseases were selected until 100 non-AA diseases had been identified. This approach was used to identify diseases associated with recent non-AA approvals while maintaining mutually exclusive AA and non-AA disease groups.

*S15: Prioritization of literature for disease burden and characteristics extraction*

To identify the source used to determine the economic burden when multiple publications were available, US-based studies and more recent studies were preferred. When US studies were not available, non-US studies were extrapolated to US healthcare costs, with a focus on studies that reported per-person, annual costs and all-payer populations. If a non-US economic burden study had to be used, the ratio of the average healthcare cost in the non-US country and the US was utilized to adjust the estimated burden to be on US terms. Peer-reviewed journal articles were prioritized, while conference papers and news articles were used when published journal papers were unavailable. National studies were also given priority over regional or single-payer studies. If all-payer studies were not available, the Medicare to Medicaid Fee Index^[1]^ and the Medicare to Commercial payment ratio^[2]^ from Kaiser Family Foundation (KFF) were used to estimate the costs for an all-payer population. If multiple studies were available for a given disease, the following factors were considered to select the best evidence: (1) more recent study/data year, (2) broader sample population, (3) more cost disaggregation, and (4) higher study credibility (i.e., citation frequency and journal impact factor).

*S16: Jiao-Basu Approach*

Jiao-Basu approach also allows for a comparison between literature-based values and estimates generated through a modeling framework. To accomplish this, average patient age and QoL estimates were derived from the economic burden literature described above. Disability days, defined as the number of days of work missed because of illness, injury, or mental or emotional problems, were estimated based on Medical Expenditure Panel Survey (MEPS) data.^[3]^ For diseases available in the data, disability days were estimated based on the matching MEPS disease category (3-digit ICD-10-CM code). As AA-treated diseases were not included in the MEPS data, these diseases were mapped to proxy disease categories to estimate disability days; non-AA-treated diseases that were unavailable in MEPS were excluded from the analysis results. AA proxy diseases were identified by asking ChatGPT (Version 4o, May 6-20, 2025) to identify the top three disease categories available in MEPS that were most clinically, epidemiologically, and symptomatically similar to each AA-treated disease. Selections were then verified based on the clinical relevance and sample size of data available in MEPS, with priority given to those with the closest clinical similarity to the AA-treated disease and a larger MEPS sample size. Based on the chosen proxies, four proxy scenarios for AAs were created: (i) “Top 1” estimated AA-treated disease disability based on the top proxy recommendation, (ii) “Top 3” estimated disability as the average disability days of the top three proxy recommendations, (iii, iv) “Sensitivity” used two times the original proxy disability estimates (“Top 1”, “Top 3”) to account for AA-treated diseases potentially being on the more severe end of the recommended disease categories. Using these estimates, the Jiao-Basu approach was then applied to calculate the hours lost across various time-use activities for both AA-treated diseases and non-AA-treated diseases. Estimates for the monetary value of hours lost was estimated by multiplying the number of hours lost by the US median hourly wage rate and inflating to 2025 USD.^[4]^ These results were then compared to the hours lost and monetary value of losses in a matched general population, which calculated losses using the same age of each matched disease, no disability, and general population health-related QoL (0.82)^[5]^, to determine the average incremental effect of AAs and non-AAs on caregiving and productivity losses.

*S17: Sensitivity Analysis (AA, N=18 vs N=16)*

The analysis using 18 AA-treated diseases revealed that AA-treated diseases tend to affect a greater proportion of the population aged <18 (29% vs 24%) and ≥65 (28% vs 23%) years, while having a lower prevalence (277 vs 2,250 per 100,000) compared to non-AA-treated diseases (Supplementary Table S12). On the other hand, using the initial AA definition (n=16) broadens differences between AA-treated diseases and non-AA-treated diseases for age of onset (5% vs. 2%), percentage of population < 18 (32% vs. 21%), percentage of the population ≥65 (24% vs. 19%), LY (-19%, -17%), QoL when alive (-7%, -5%), prevalence (-86%, -88%), and disease duration (-14%, -8%) while the gap in life expectancy is similar (Supplementary Table S14). When comparing the initial 16 AA-treated diseases to the expanded set of 18 AA-treated diseases, the gap in economic burden narrowed slightly when using the initial AA definition, particularly with regards to social costs (40.3% vs 46.2%) (Supplemental Figure S16 - S17).

*S18:* *Economic Burden of Diseases with US-only sources*

Among the diseases with economic burden collected from US only sources, the overall societal costs for AA-treated diseases are slightly lower ($85,097 vs $91,857) compared to all (US and Non-US studies). The lower bound scenarios demonstrate more differences in the US-only data, with higher percentage differences in non-medical costs (374.6% vs 127.6%), productivity costs (252.2% vs 168.9%), and caregiver burden (481.0% vs 421.3%). The upper bound scenarios show more moderate differences, with the US-only data revealing lower societal cost disparities (30.1% vs 31.3%) but higher differences in direct medical costs excluding prescriptions (98.9% vs 72.4%).

1. Medicaid-to-Medicare Fee Index, Kaiser Family Foundation, 2024: https://www.kff.org/medicaid/state-indicator/medicaid-to-medicare-fee-index/
2. Eric Lopez, Tricia Neuman, Gretchen Jacobson, and Larry Levitt (2020). How Much More Than Medicare Do Private Insurers Pay? A Review of the Literature, Kaiser Family Foundation.
3. Medical Expenditure Panel Survey, Agency for Healthcare Research and Quality: https://meps.ahrq.gov/mepsweb/survey_comp/survey_results_ques_sections.jsp?Section=DD&Year1=2021&Submit1=Search.
4. Occupational Employment and Wage Statistics, U.S. Bureau of Labor Statistics, May 2024; U.S. Bureau of Labor Statistics, Consumer Price Index for All Urban Consumers: All Items in U.S. City Average [CPIAUCSL], retrieved from FRED, Federal Reserve Bank of St. Louis.
5. Author’s Assumption
